# Simple Topological Features Reflect Dynamics and Modularity in Protein Interaction Networks

**DOI:** 10.1371/journal.pcbi.1003243

**Published:** 2013-10-10

**Authors:** Yuri Pritykin, Mona Singh

**Affiliations:** 1 Department of Computer Science, Princeton University, Princeton, New Jersey, United States of America; 2 Lewis–Sigler Institute for Integrative Genomics, Princeton University, Princeton, New Jersey, United States of America; University of Chicago, United States of America

## Abstract

The availability of large-scale protein-protein interaction networks for numerous organisms provides an opportunity to comprehensively analyze whether simple properties of proteins are predictive of the roles they play in the functional organization of the cell. We begin by re-examining an influential but controversial characterization of the dynamic modularity of the *S. cerevisiae* interactome that incorporated gene expression data into network analysis. We analyse the protein-protein interaction networks of five organisms, *S. cerevisiae*, *H. sapiens*, *D. melanogaster*, *A. thaliana*, and *E. coli*, and confirm significant and consistent functional and structural differences between hub proteins that are co-expressed with their interacting partners and those that are not, and support the view that the former tend to be intramodular whereas the latter tend to be intermodular. However, we also demonstrate that in each of these organisms, simple topological measures are significantly correlated with the average co-expression of a hub with its partners, independent of any classification, and therefore also reflect protein intra- and inter- modularity. Further, cross-interactomic analysis demonstrates that these simple topological characteristics of hub proteins tend to be conserved across organisms. Overall, we give evidence that purely topological features of static interaction networks reflect aspects of the dynamics and modularity of interactomes as well as previous measures incorporating expression data, and are a powerful means for understanding the dynamic roles of hubs in interactomes.

## Author Summary

A better understanding of protein interaction networks would be a great aid in furthering our knowledge of the molecular biology of the cell. Towards this end, large-scale protein-protein physical interaction data have been determined for organisms across the evolutionary spectrum. However, the resulting networks give a static view of interactomes, and our knowledge about protein interactions is rarely time or context specific. A previous prominent but controversial attempt to characterize the dynamic modularity of the interactome was based on integrating physical interaction data with gene activity measurements from transcript expression data. This analysis distinguished between proteins that are co-expressed with their interacting partners and those that are not, and argued that the former are intramodular and the latter are intermodular. By analyzing the interactomes of five organisms, we largely confirm the biological significance of this characterization through a variety of statistical tests and computational experiments. Surprisingly, however, we find that similar results can be obtained using just network information without additionally integrating expression data, suggesting that purely topological characteristics of interaction networks strongly reflect certain aspects of the dynamics and modularity of interactomes.

## Introduction

A better understanding of protein interaction networks would be a great aid in furthering our knowledge of the molecular biology of the cell. Towards this end, large-scale protein-protein interaction (PPI) networks have been determined for a diverse set of model organisms and for human [1]–[12]. Computational analyses of these networks have revealed many important aspects of cellular organization and functioning [13], [14], including a strong link between the topological characteristics of cellular networks and their underlying functioning. One fundamental finding is that PPI networks are modular: they tend to consist of groups of tightly interacting proteins corresponding to functional modules or protein complexes [15]–[20]. Further, from early on, it has been apparent that hubs—proteins participating in many interactions—have special roles in PPI networks: they tend to be more essential, more evolutionarily conserved, and in human are enriched in genes over-expressed or mutated in cancers [21]–[28]. It is naturally interesting to combine these two well-studied views of PPI networks and to ask how hubs are positioned with respect to the modular organization of the cell.

Specific contextual information about interactions would be of great help in understanding the connection between hubs and modularity. For most interactions of a protein in a network, however, we typically do not know whether these interactions occur at the same time or under different conditions. In order to understand the dynamic roles of hubs and their relationship to network modularity, a highly influential study integrated PPI data with gene expression data measured in numerous conditions, and classified hubs based on their average co-expression with their interacting partners [29]. Hubs that have high average co-expression with their partners were classified as “party,” as they are likely to interact with these other proteins at the same time. Conversely, hubs that have low average co-expression with their partners were classified as “date,” as they are likely to interact with their partners at different moments of time. In an analysis of the *S. cerevisiae* interactome, date and party hubs were shown to exhibit different biological properties that imply different roles in the PPI network. In particular, date hubs were found to have more diversity in their subcellular localizations, a more drastic effect on network connectivity when removed from the network, higher centralities in a network of genetic interactions, and higher evolutionary rates [29]–[31]. Further, it was argued that date hubs are global connectors of different modules whereas party hubs are more local and play specific roles in modules.

Though the classification of hubs into party and date has been generally accepted [6], [24], [30], [32]–[34], it has also generated significant controversy [35]–[37]. It has been proposed that the observed date/party hub distinction may be an artefact of biases in the datasets used or of the analysis methodology. Whereas the initial work [29] observed a bimodality in the distribution of average co-expression across hubs and used this to partition hubs into party or date, this bimodality has not been observed in subsequent studies [31], [35]–[37]. Further, it has been suggested that the difference in the effect on network connectivity of removing either all the date or party hubs is attributable to a difference in the total number of interactions of date and party hubs, that date and party hubs evolve at the same rate, and that there is no evidence of different centrality in the genetic network for date and party hubs [35], [36]. Finally, it has been argued that the observed differences in the topological properties between date and party hubs in the network may be attributable only to a small number of date hubs with extreme properties, while the remaining hubs are much more homogeneous [37].

The current availability of large-scale interaction networks for numerous organisms across the evolutionary spectrum provides us with an opportunity to systematically analyze whether properties of hubs are predictive of the roles they play in the functional organization of cellular networks. We use interaction networks for five organisms, *S. cerevisiae*, *H. sapiens*, *D. melanogaster*, *A. thaliana*, and *E. coli*, along with multiple mRNA expression datasets. In each of these organisms, we show that the average co-expression of a hub with its partners, independent of any categorization of hubs, reveals important properties of hubs: the average co-expression of a hub with its interacting partners is significantly positively correlated with its local clustering coefficient as well as its average biological process similarity with its interacting partners, and is significantly negatively correlated with its betweenness centrality and its participation coefficient (a topological measure that reflects the diversity of the intermodular interactions of a protein). Further, the average co-expression of a hub with its interacting partners is negatively correlated with its interaction degree in genetic networks, and positively correlated with protein essentiality. Importantly, the correlations uncovered between average co-expression and topological features—independent of any classification of hubs—imply that the topological features of hubs by themselves reflect important aspects of the dynamics and modularity of the interactome. For example, hubs with low betweenness or high clustering coefficient will tend to have high average co-expression with their partners and fewer genetic interactions, whereas proteins with high betweenness or low clustering coefficient tend to exhibit the opposite trends. Further, as part of our study, we revisit the date-party controversy. We consider a very simple criterion to classify hubs as either party or date, and confirm significant and consistent functional and topological differences in the properties of date and party hubs across organisms. Finally, in a cross-interactomic analysis, we demonstrate that these simple topological and co-expression properties of hub proteins tend to be conserved across organisms, giving further evidence that these features reflect important aspects of cellular functioning.

## Results

### Preliminaries

We begin by briefly describing our data and analysis framework (see **Materials and methods** for details). We analyze PPI networks for *H. sapiens*, *S. cerevisiae*, *D. melanogaster*, *A. thaliana*, and *E. coli* (denoted by **Human-all** , **Yeast-all** , **Fly** , **Athal**, and **Ecoli**, respectively). For human and yeast, we additionally consider networks consisting of high-confidence interactions only (denoted by **Human-hq** and **Yeast-hq**). We gather mRNA expression data for these organisms from GEO [38], and compute a co-expression score for each interaction using the Pearson correlation coefficient (PCC). We define hubs as all genes in the top 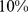 in each interactome by the number of interactions. For each hub, we calculate the average of the co-expression scores (avPCC) computed over all the interactions in which this hub participates. The size of each network, the number of interactions with expression data, and the number of hubs are listed in Table 1. In the main text, we focus on the human high confidence interaction network **Human-hq** unless otherwise specified, but results for all networks are given in the **Text S1**.

**Table 1:** Network sizes and the number of network hubs.

Network	Num genes	Num interactions	Num interactions with co-expression score	Hub threshold	Num hubs (%)	Num hubs with avPCC
**Human-hq**	4,750	13,102	11,781	12	481 (10.1%)	466
**Yeast-hq**	4,467	22,243	21,869	23	449 (10.1%)	445
**Fly**	8,218	36,569	36,525	23	865 (10.5%)	865
**Athal**	5,454	12,883	10,611	10	555 (10.2%)	506
**Ecoli**	3,115	17,788	17,697	23	319 (10.2%)	319
**Human-all**	10,229	80,651	66,102	39	1,033 (10.1%)	931
**Yeast-all**	5,641	59,930	59,658	49	570 (10.1%)	570

The number of vertices (genes) and edges (interactions) in each network, the number of interactions that were assigned a co-expression score, the degree threshold to be chosen as a hub, the number of hubs obtained using this threshold, and the number of hubs that were assigned an average co-expression score.

We use four measures to ascertain the functional, organizational and dynamic properties of proteins in the network: clustering coefficient, betweenness centrality, participation coefficient and functional similarity. Clustering coefficient is the density of the neighborhood of a protein in the network, and proteins with higher clustering coefficient have interactions with proteins that interact with each other. Betweenness centrality is a measure of the fraction of shortest paths passing through a node in the network, and nodes with higher betweenness are more globally central in the network. Participation coefficient shows how well interactions of a protein are distributed amongst clusters in the network, so that proteins with low participation are mostly interacting with proteins from the same cluster, whereas proteins with high participation have their interactions spread among many clusters. Functional similarity estimates to what extent a protein participates in the same biological process as its neighbors in the network. We note that three of these measures are purely topological and do not use any information other than interaction data, whereas functional similarity also uses Gene Ontology [39] annotations.

We classify hubs in the low range of avPCC as date and hubs in the high range of avPCC as party. Despite the previously observed differences between date and party hubs [6], [24], [29], [30], [32]–[34], the choice of a threshold in the avPCC range between the two classes of hubs has remained a topic of disagreement. As we have many networks to consider, we choose a simple threshold criterion and later demonstrate that this choice does not matter (see section **Hub characteristics**). In particular, we define party hubs as the one third of hubs with the largest avPCC, and call the remaining two thirds of hubs date. Since it has been argued that the originally observed differences between date and party hubs may be attributed only to a small number of date hubs with extreme network global centrality properties [37], we classify hubs with many interactions or with high betweenness centrality into a special group called extremal hubs (18 to 89 hubs depending on the network) and exclude them from the analysis of date and party hubs.

### Properties of date and party hubs are significantly distinct

**Figure 1:**
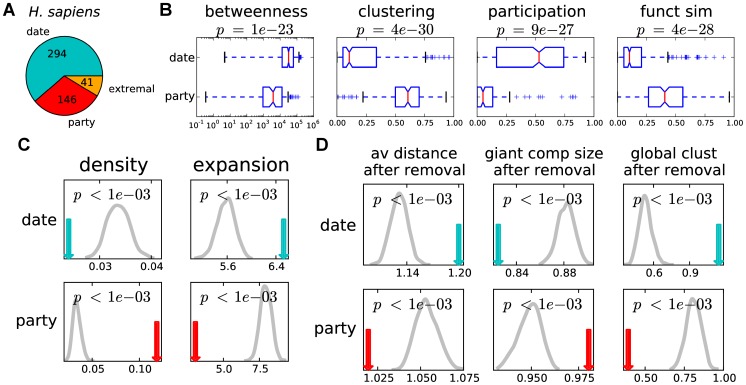
Date and party hubs have distinct functional and topological properties. Date and party hub classification analysis in the human high quality interaction network (**Human-hq**). (A) Number of hubs in each class. Party hubs in this network have avPCC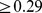; this threshold corresponds to the top third of avPCC values for all hubs categorized as either party or date. (B) Betweenness, clustering coefficient, participation coefficient and functional similarity for date and party hubs are significantly different; p-values are computed using the Mann–Whitney U. (C) Density and expansion of date and party hubs are significantly different. The gray curves in each panel show the distributions for 1000 independent random samples of the same number of hubs, and are used to compute empirical p-values. (D) Effect of hub removal is significantly different for party and date when considering the average path distance, the size of the largest connected component, and the global clustering coefficient. The gray curves show the distributions for 1000 independent random samples of the same number of hubs, and are used to compute empirical p-values. See **Materials and methods** for more details.

We first analyze the differences between party and date hubs on our seven networks (Fig. 1 and Fig. S1, Fig. S2, Fig. S3, Fig. S4, Fig. S5 and Fig. S6). We confirm that date hubs tend to be more globally central in the network and to have more diverse intermodular participation, as reflected by their significantly higher betweenness centrality and participation coefficient (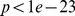 and 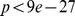 respectively, in the high confidence human network, Mann–Whitney U; Fig. 1B). Further, party hubs tend to have denser neighborhoods consisting of genes with more similar functions, as reflected by their significantly higher clustering coefficient and functional similarity (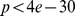 and 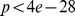, respectively, in the high confidence human network, Mann–Whitney U; Fig. 1B).

In addition to comparing the node-level features of date and party hubs, we also compare the positioning of the set of date and party hubs in the network with respect to each other in order to better understand global network organizational features. For either the set of date hubs or the set of party hubs, we measure how well connected they are to each other by calculating the density of the subnetwork induced by them, defined as the number of interactions amongst the set of proteins, normalized by the maximum possible number of such interactions. We also measure how well spread the interactions of these hubs are in the whole network by calculating the expansion of the set, defined as the number of proteins in the network that are connected with any hub in the set, but do not belong to the set, normalized by the size of the set. We observe that party hubs have a strong tendency to interact with other party hubs, and much less so with other proteins, as reflected by their high density and low expansion in the network as compared with sets of the same size consisting of randomly selected hubs (Fig. 1C). On the contrary, date hubs have significantly lower density and significantly higher expansion than random sets with the same number of hubs, suggesting that they are more sparsely distributed in the network than party hubs.

As a final test to compare the topological features of date and party hubs, we compare the effect of node removal on network structure for date and party hubs. For a set of hubs (either date or party), we remove all of them from the network at once and measure the change in three representative global network characteristics: average path length, size of the largest connected component, and global clustering coefficient. We compare the effect of such a removal with the effect of a removal of random sets of the same number of hubs. We observe that date hubs are more central in the network, as their removal affects connectivity of the network much more significantly than removal of random hubs, as reflected by the average path length of the network and the size of the largest connected component (Fig. 1D). At the same time, removal of party hubs makes the network much less clustered than the removal of random hubs, as reflected by the effect on the global clustering coefficient.

The results of these analyses have qualitatively the same trends across the five organisms (Fig. S1, Fig. S2, Fig. S3, Fig. S4, Fig. S5 and Fig. S6), if extremal hubs are included in the analysis (Fig. S7), or if all proteins with at least three interactions in the network are considered hubs (see Section S1.2 in **Text S1** and Fig. S8, Fig. S9 and Fig. S10). Taken together, our analysis over seven networks suggests significant and consistent differences between proteins characterized based on avPCC with respect to topological, intermodular and functional features.

### Hub characteristics capture functional and organizational properties of the interactome

**Figure 2:**
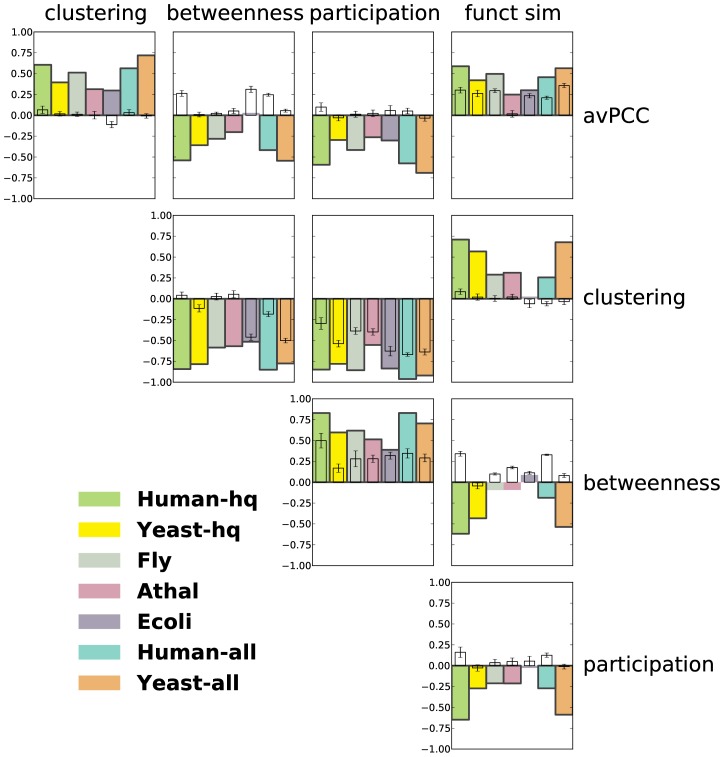
Functional and topological characteristics of hubs are significantly correlated with each other in a consistent manner in protein-protein interaction networks. Every colored bar represents a Spearman correlation between two characteristics of hubs in one of the networks. Bars of significant correlations (absolute value 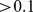, p-value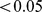) have black edges. See Tables S1, S2, S3 and S4 for exact values. Smaller uncolored bars show average correlations in 20 degree-preserving random networks (with error bars depicting standard deviations).

We next show that the avPCC measure is an interesting biological measure independent of any threshold one could use to define date and party hubs. That is, while there has been significant previous controversy concerning how an avPCC threshold should be chosen to categorize hubs into date and party [35], [36], we show here that the avPCC measure is itself correlated with other characteristics of hubs in the network. In particular, we compute the Spearman rank correlation (SRCC) between avPCC and our topological and functional measures (Fig. 2, top row, and Table S1). Across the organisms, we find consistent positive correlations of avPCC with clustering (SRCCs ranging from 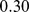 to 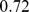 depending upon the network) and functional similarity (SRCCs from 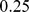 to 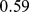) and negative correlations with betweenness (SRCCs from 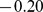 to 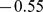, except **Ecoli** ) and participation (SRCCs from 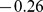 to 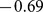). These correlations are consistent with the original claims [29] that hubs in the high avPCC range are more local and play more central roles within modules and complexes, and thus have higher clustering coefficients and higher average functional similarities with their interacting partners, whereas hubs in the low avPCC range play more global roles in organizing other proteins' functioning and thus are more globally central in the PPI network (as evidenced by higher betweenness centrality) and have more diverse participation in interactions with different processes and modules (as evidenced by higher participation coefficient).

Given the significant and consistent correlations between avPCC and clustering, betweenness, participation and functional similarity, we also compute the SRCCs amongst these measures. As expected from our above analysis, these measures are also correlated with each other in a consistent manner across the seven networks (Fig. 2 and Tables S2, S3 and S4). Comparing the three purely topological measures with each other, we find that betweenness is positively correlated with participation, while both are negatively correlated with clustering. We further note that because the functional similarity measure aggregates information from Gene Ontology, we can also use it as an independent means of assessing whether the topological measures based purely on interaction data reflect properties of protein functioning. We find that functional similarity is significantly positively correlated with clustering (SRCC from 0.26 to 0.71, except **Ecoli** ), while negatively correlated with betweenness (SRCC from 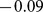 to 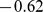, except **Ecoli** ) and participation (SRCC from 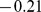 to 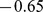, except **Ecoli** ). This suggests that measures based purely on the topology of the network can reflect interesting functional properties of proteins.

As a control to confirm that the information coming from protein-protein interactions is crucial, we randomize each network in a degree-preserving manner [40], and recompute the node-level topological and functional measures using the randomized interactions. Correlations between these measures aggregated over 20 random networks (Fig. 2) have substantially lower absolute values than for real interaction networks and sometimes show a completely opposite trend. We note, however, that these measures can still have significant and meaningful correlations in random networks. For example, a remarkably high correlation is found between avPCC and functional similarity in random networks, though it is still noticeably lower than in real networks. This is an indication of the strong signal in expression data itself that does not arise from physical interactions. Indeed, it is not surprising that even for arbitrary pairs of genes, not necessarily physically interacting, the more often they are expressed together, the more likely that they are functionally related.

Potentially confounding factors in our correlation analysis include the protein degree threshold used to identify hubs in the networks, correlations of hub features with degree, bias from extremal hubs, and study bias. To demonstrate that none of these significantly affect our results, we also perform this analysis when using different degree thresholds, when computing partial correlations with correction for degree, when excluding extremal hubs, and when focusing on high-throughput networks in yeast and human (see Sections S1.2 and S1.3 in **Text S1** and Fig. S11, Fig. S12, Fig. S13, Fig. S14 and Fig. S15).

### Distinct functions are enriched in hub classes

**Figure 3:**
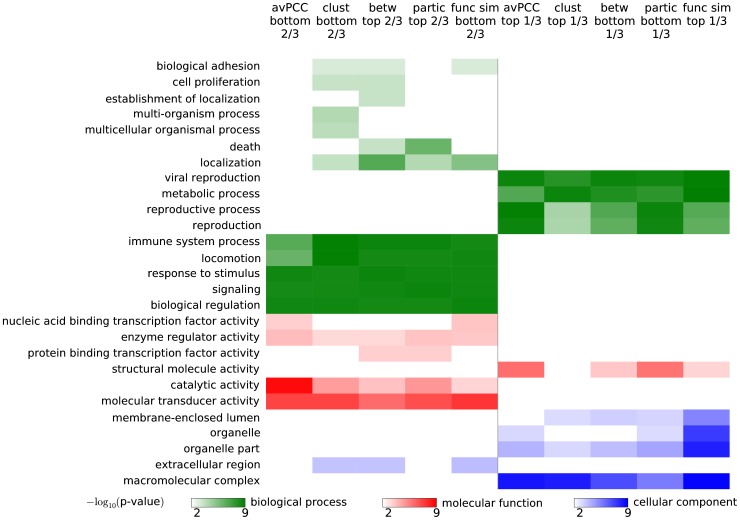
Different hub characteristics produce classifications of hubs with similar functional properties. Hubs are divided in a 2-to-1 proportion using either avPCC, clustering coefficient, betweenness centrality, participation coefficient or functional similarity scores in **Human-hq**. Broad GO terms that are enriched at Bonferroni-corrected significance threshold 0.05 are shown with colors indicating the ontology of the term and color intensity indicating the p-value of enrichment. Classifications of hubs based on different hub characteristics produce classes of hubs with similar functional annotations.

To determine whether party and date hubs (as well as hubs partitioned into groups based on topological measures) tend to participate in different biological functions, we performed GO enrichment analysis on each set of hubs in **Human-hq** using the most general terms in each of the three ontologies (i.e., the terms that are immediate children of roots of the ontologies), and used all annotated hubs to provide the background functional distribution. We found that terms enriched for date and party hubs are very different: date hubs are associated with global tasks such as “biological regulation” and “signaling,” while party hubs are enriched in local and module- and complex-specific terms such as “macromolecular complex” and “metabolic process” (Fig. 3).

Interestingly, very similar terms are enriched when, instead of a date-party classification based on avPCC, hubs are classified in a 2-to-1 proportion using clustering coefficient, betweenness centrality, participation coefficient or functional similarity (Fig. 3). That is, the same functional terms have similar enrichments when hubs are classified based purely on topological measures, suggesting that these topological properties can reflect the functional roles of hubs in the interactome as well as avPCC does. In general, we obtain similar results for the other networks (see Section S1.4 in **Text S1** and Fig. S16, Fig. S17, Fig. S18, Fig. S19, Fig. S20 and Fig. S21).

### Hubs that are more globally central in physical interaction networks have more genetic interactions

We next consider the relationship between various properties of hubs in physical interaction networks and their number of genetic interactions. In the initial publication on date and party hubs [29], it was observed that date hubs are involved in more genetic interactions than party hubs, and it was proposed that their phenotypic link to many proteins was due to their connecting different biological processes to each other [29]. As yeast remains the only organism with a sufficiently large number of known genetic interactions, our analysis on genetic interactions is limited to this organism. We note, however, that the current dataset aggregated in BioGRID [41] is two orders of magnitude larger than the one used previously by Han *et al.* [29].

**Figure 4:**
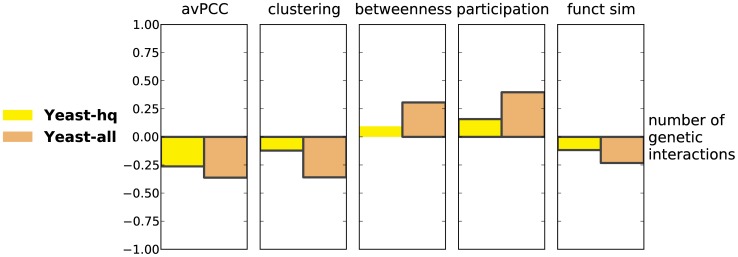
Characteristics of hubs in protein physical interaction networks are significantly correlated with their number of genetic interactions. Every bar represents a Spearman correlation between a hub characteristic in one of the protein physical interaction networks for yeast and degree in the yeast genetic interaction network. Bars of significant correlations (absolute value 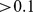, p-value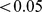) have black edges.

We compute SRCCs between the number of genetic interactions a hub has and its avPCC, its clustering coefficient, its betweenness centrality, its participation coefficient and its functional similarity in the physical interaction network. For both the **Yeast-all** and **Yeast-hq** networks, avPCC is significantly negatively correlated with genetic interaction degree (Fig. 4). Further, we find that the number of genetic interactions of a hub is positively correlated with betweenness and participation in both networks, while negatively correlated with clustering and functional similarity. We also confirm that these correlations are significant even when compared with those in random networks (see Section S1.8 in **Text S1** and Fig. S34). Finally, to directly compare with the original study [29], we also verify that date hubs are involved in many more genetic interactions than party hubs (Fig. S22AB).

These observations are also largely confirmed when negative and positive genetic interactions are considered separately (Fig. S23), as well as when computing partial correlations corrected for essentiality (see Section S1.5 in **Text S1** , Fig. S22CD and Fig. S24). As is the case when considering all genetic interactions together, the trends are stronger in **Yeast-all** than in **Yeast-hq**.

Overall, we find that not only avPCC, but also other hub characteristics, including those that are purely topological, are significantly correlated with centrality in the genetic interaction network. These results support the original observations that hubs with the role of global connectors and organizers of the interactome, as identified by avPCC or (as we show here) by other topological measures, are related in their effect on phenotype with many more genes than are local hubs from modules and complexes.

### Role of yeast two-hybrid and co-complex interactions

Physical protein-protein interactions obtained using different methods can differ in their characteristics [6], [42]. In particular, the two high-throughput methods that account for the largest number of interactions in our networks, yeast two-hybrid (Y2H) and affinity purification followed by mass spectrometry, tend to detect different types of interactions. The former are more likely to detect direct, transient binary interactions between proteins whereas the latter tend to detect more stable co-complex interactions that may or may not correspond to direct interactions.

It was previously observed that for a fixed avPCC threshold in the definition of date and party hubs, date hubs are much more prevalent in Y2H networks, while party hubs are more prevalent in co-complex networks [6]. Therefore it was suspected that the observed distinction between date and party hubs may be attributable to the fact that interaction networks are typically compiled of interactions of both types, and this may artificially imply the date/party distinction [37]. In order to rule out these concerns regarding our observations about topological features of hub proteins, we apply the same analysis to networks of only Y2H or of only co-complex interactions.

We find that correlations between different hub characteristics for networks formed by either only yeast two-hybrid or only co-complex interactions are qualitatively the same as in networks with interactions of all types combined (Fig. S25, as compared with Fig. 2). The date/party distinction for hubs in these networks separately is also qualitatively the same as in networks with interactions of both types combined (Fig. S26, Fig. S27, Fig. S28, Fig. S29, Fig. S30 and Fig. S31, compare with Fig. 1 and Fig. S1, Fig. S2, Fig. S3, Fig. S4, Fig. S5 and Fig. S6). Thus, simple hub characteristics consistently reflect principles of network structure and functioning even when applied to networks comprised of either Y2H or co-complex interactions.

**Figure 5:**
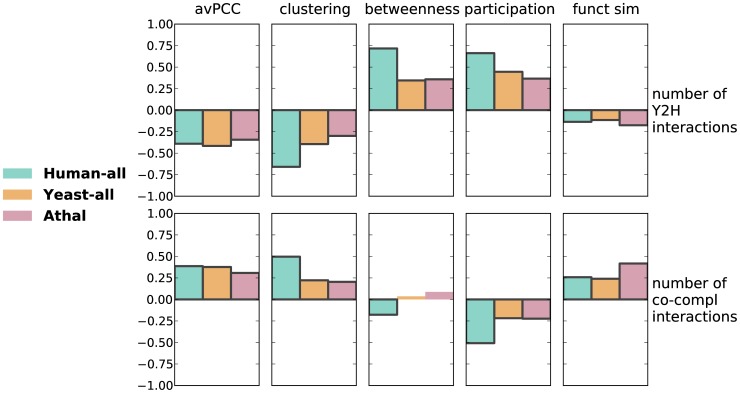
Hubs with different roles in interactomes are involved in interactions of different types. For hubs and their characteristics determined from the full networks **Human-all**, **Yeast-all** and **Athal**, we measured the Spearman correlation between hub characteristics and the number of interactions of the type yeast two-hybrid or co-complex. Bars of significant correlations (absolute value 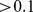, p-value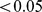) have black edges. Hubs with higher avPCC, clustering coefficient and functional similarity tend to have more co-complex interactions, while hubs with higher betweenness and participation coefficient tend to have more yeast two-hybrid interactions.

Despite the consistency in correlations amongst hub features between Y2H or co-complex networks with the network compiled of interactions of both types, when analyzing just the latter combined network, topological properties of hubs are consistently and oppositely correlated with the number of Y2H interactions as opposed to the number of co-complex interactions. The avPCC measure is negatively correlated with the number of Y2H interactions, while positively correlated with the number of co-complex interactions (Fig. 5). Accordingly, date hubs participate in more Y2H interactions, while party hubs participate in more co-complex interactions (p-value from 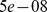 to 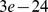, Mann–Whitney U; Fig. S32). Betweenness and participation are positively correlated with the number of Y2H interactions, while negatively correlated with the number of co-complex interactions, which suggests that these two measurements are indeed capturing the centrality of hubs and their tendency to interact one-to-one with other proteins. Clustering and functional similarity are negatively correlated with the number of Y2H interactions, while positively correlated with the number of co-complex interactions, which suggests that these two measurements are capturing the tendency of hubs to participate in complexes and functionally homogeneous modules. Thus, we find that more globally central hubs (as specified by either betweenness or participation) tend to have more yeast two-hybrid interactions whereas more module-specific hubs (as specified by either avPCC, clustering coefficient or functional similarity) tend to have more co-complex interactions.

### Hubs involved in modules and clusters are more likely to be essential

In the initial study [29], it was observed that in yeast, party hubs are more likely to be essential than date hubs (though the observed difference was not significant). We revisit this question with our newer and larger data set.

**Figure 6:**
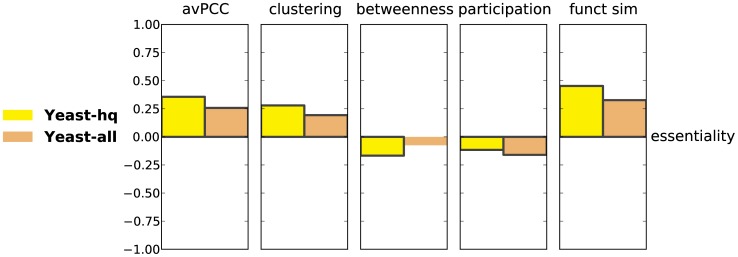
Hub characteristics in yeast protein physical interaction networks are correlated with protein essentiality. Each bar represents a Spearman correlation between a hub characteristic and hub essentiality in one of the yeast networks. Bars of significant correlations (absolute value 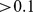, p-value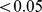) have black edges.

We compute the SRCC between essentiality represented as an indicator vector (i.e., 1 if a gene is essential and 0 otherwise) and other characteristics of hubs in the physical interaction network. For both the **Yeast-all** and **Yeast-hq** networks, avPCC is significantly positively correlated with essentiality (Fig. 6). To directly compare with the original study [29], we also compare date and party hubs and find a significantly larger fraction of essential genes in the set of party hubs than in the set of date hubs, as determined by the hypergeometric test (Fig. S33). We further show that the correlation of avPCC and essentiality is significantly high even when compared with that found in random networks (see Section S1.8 in **Text S1** and Fig. S35). We also find that essentiality is positively correlated with clustering and functional similarity, while negatively correlated with betweenness and participation (though this correlation is not significant for betweenness in **Yeast-all**). This is in agreement with recent evidence of the tight relationship of a protein's essentiality with modularity and its involvement in essential complexes [22], [23], as hubs with high avPCC, clustering, or functional similarity, and correspondingly low betweenness and participation, are likely to play key roles in modules and complexes.

### Hub roles in the interactome are evolutionary conserved

In order to compare hub characteristics for similar genes from different organisms, we obtain sets of orthologous proteins from P-POD [43] for all organisms under consideration. As genes from the *E. coli* network have only a few orthologs in P-POD in the other networks, we focus this analysis on the four eukaryotic species. For each pair of networks of different organisms, we calculate the SRCCs of hub characteristics (avPCC, clustering, betweenness, participation and functional similarity, computed as described above independently for each network) over all pairs of orthologous hubs. For **Human-all** and **Yeast-all** we observe highly significant positive correlations which range from 0.38 for functional similarity to 0.76 for participation (Table 2), and for **Human-hq** and **Yeast-hq** they range from 0.23 for avPCC to 0.62 for clustering (Table S5). We note that some proteins may be involved in many orthologous pairs and therefore we also validate the significance of the observed correlations by randomly permuting hubs, and find that these results remain significant (see **Materials and methods** for more details). These features are largely consistently positively correlated when comparing ortholog pairs between different pairs of networks (Tables S6, S7, S8, S9, S10), though at varying levels of statistical significance. Further, we observe that purely topological features such as clustering coefficient and betweenness centrality are much more consistently conserved between pairs of networks than avPCC (Tables 2, S5, S6, S7 and S8), which is additional evidence that these topological features can reflect hub roles in the interactome.

**Table 2:** Spearman correlation for characteristics of orthologous hubs in Yeast-all and Human-all.

Hub characteristic	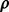	p-value	Empirical p-value
avPCC	**0.55**	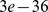	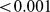
clustering	**0.72**	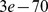	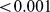
betweenness	**0.44**	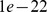	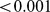
participation	**0.76**	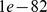	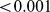
func. sim	**0.38**	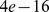	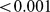

Five hub characteristics for all 437 orthologous pairs between 291 hubs in **Yeast-all** and 299 hubs in **Human-all** are significantly positively correlated, as measured by Spearman's rho (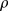) and the correspondingly determined p-values and empirical p-values for 1000 random permutations of hubs. See main text and **Materials and methods** for details.

**Figure 7:**
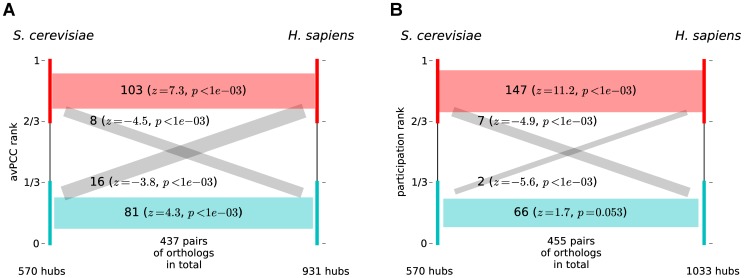
Characteristics of hubs are conserved across networks. The top (respectively, bottom) third of hubs in each of **Yeast-all** and **Human-all**, as determined by (A) avPCC and (B) participation coefficient, are enriched in the number of orthologs between them. The number of orthologs between each of the groups is given, along with a z-score and p-value derived empirically from random samples of proteins for each group. Red bars indicate orthologous relationships between proteins in the top third of hubs, blue bars indicate orthologous relationships between proteins in bottom third of hubs, and gray bars indicate orthologous relationships between proteins that are in opposing groups in the two organisms.

One may suspect that the observed high correlation of hub features between organisms may be explained by conservation of modules that correspond to higher values of avPCC, clustering and functional similarity and to lower values of betweenness and participation. To exclude this possibility, for a pair of organisms, we first determine in each the hubs with the highest and lowest third of scores, according to any given hub measure. Next, we determine how many ortholog pairs are found between each of the top and bottom groups in both organisms. We compare these numbers with the same values expected if these top and bottom sets of hubs were selected at random, rather than according to the hub score. We expect more ortholog pairs between the top thirds as well as between bottom thirds, and fewer orthologs between the top and bottom thirds. Indeed, this is what is observed for our hub measures (see, for example, avPCC when comparing the **Yeast-all** and **Human-hq** networks in Fig. 7A and participation for these networks in Fig. 7B).

## Discussion

We have confirmed in protein interaction networks across a range of organisms that if hubs are partitioned into two classes according to their tendency to be co-expressed with their interacting partners, they exhibit significantly different properties and roles in the interactome. In one class, hubs tend to have higher average co-expression with their interacting partners, higher clustering coefficients and higher functional similarities, but lower betweenness centralities and participation coefficients. These hubs are more often interacting with each other, and are enriched with co-complex interactions. Simulated removal of these hubs from the network does not greatly affect the connectivity of the network. These properties suggest that such hubs may act locally inside functional modules and protein complexes. In another class, hubs tend to have lower average co-expression, clustering, and functional similarity, but higher betweenness and participation. These hubs more often participate in genetic interactions, and are more often detected in yeast two-hybrid interactions, which are presumably enriched in binary transient interactions. These hubs tend to interact with each other less, and with other proteins more. After these hubs are removed, the network becomes more disconnected and clustered. These properties suggest that such hubs tend to be global connectors and coordinators of different modules in the interactome.

Initially, it was proposed that the distribution of the hubs' average co-expressions with their neighbors was bimodal, and it was argued that this naturally implied a categorization of all hubs into two classes with hypothetically different roles. Furthermore, Han *et al.* [29] proposed a view of the interactome with mostly non-intersecting independent modules, and certain proteins outside of these modules that connect and coordinate their functioning. This model, as well as the existence of the two classes of hubs with correspondingly different roles, has been the subject of some controversy. We argue that even though we believe the two classes of proteins in the interactome can be distinguished from each other and their roles can be recognized as different, it is not necessarily the case that all proteins or even just all hubs can be classified into one of the classes. Rather, the network is almost certainly more complex, with highly overlapping modules, multifunctional proteins, and proteins of mixed and not easily detectable roles that depend upon conditions and time. A better understanding of the structure and functioning of the interactome will require large-scale annotation of interactions and interacting proteins with information about concentrations and the strength, condition and timing of when, where and how these interactions occur. These annotations are currently not available at a large scale, but may be obtained experimentally in the future, or by developing new methods for analysis of existing data. With uncertainty about the exact role of each particular protein in the interactome, measuring and analyzing their properties on a continuous scale may be more appropriate than trying to extract firm classes.

A significant amount of computational research has been devoted to uncovering the dynamics of protein interactions via integration with other types of data [18], [44]–[47]. Currently, the most common approach to glean information about the dynamics of hubs and their interactions is by integrating interaction data with gene expression data, as is done here and previously using the measure of a protein's average co-expression with its interacting partners. However, we have shown that very similar information is reflected in the interaction data itself. Even though it is highly unlikely that just topological network features can describe all of the structure and dynamics of interactomes, analysis of topological characteristics in networks may be of great help in furthering our understanding of network dynamics.

As more large-scale protein interaction networks have become available, certain of their aspects and properties have been shown to be conserved across interactomes of different organisms [48]–[52]. Such conservation is strong evidence that a network feature reflects an important aspect of interactomes. We have shown that a protein's average co-expression over neighbors in its PPI network is conserved for orthologous hubs across different organisms, and have further confirmed that it is a biologically meaningful measure for understanding hub roles. At the same time, we have shown that hub characteristics that depend purely on network topology are conserved at least as well as average co-expression.

Following previous work, in our analysis we have focused almost exclusively on hubs, a small fraction of proteins within interactomes. However, we have also demonstrated, by reducing the number of interactions required to call a protein a hub, that our observations hold when we consider many more proteins in the network, so it may be possible to classify not just hubs based on topological features or co-expression properties, but also proteins in general. Moreover, we have shown that our analysis is robust to noise in interaction data, as the trends we report are consistent not only across networks of lower coverage where interactions are additionally selected for high quality, but also across larger networks without additional quality filtering that are likely to contain more noise but also have higher coverage.

We have shown that topological features of proteins in the network capture functional and structural properties of networks. Therefore, the distribution of these features also, to some extent, characterizes the whole interactome. In the future, depending upon the application, it may be desirable to take these features into account when building and analyzing models for protein interaction networks, and in particular, within algorithms that are used for generating random networks in order to compare them with real data. Existing approaches for randomizing protein interaction networks have preserved local properties such as degree and local clustering coefficient, small subgraphs and schemas, as well as some evolutionary constraints [52]–[57]. In addition to these features, in the future, randomization algorithms may try to also preserve measures such as betweenness centrality and participation coefficient, as we have demonstrated that these features capture additional information about network structure.

In sum, our observations provide a better understanding of the dynamic interactome of the cell. As more specific, high-quality and high-coverage protein-protein interaction data become available, we believe our approaches to analyze these data can reveal further details about the structure, function and evolution of interactomes.

## Materials and Methods

### Interaction networks

Seven interaction networks for five organisms are considered in our analysis. We briefly describe the networks below; further details can be found in Section S2.2 in the **Text S1**. In all networks, self-loops and duplicate interactions are deleted. The size of each network is shown in Table 1.

***S. cerevisiae***. The network **Yeast-all** consists of all yeast protein physical interactions from BioGRID [41] version 3.1.78. The high quality network **Yeast-hq** consists of all binary and co-complex interactions from HINT [58]. Yeast genetic interactions are obtained from BioGRID version 3.1.78 (123707 interactions).

***H. sapiens***. We use two human protein-protein physical interaction networks, both compiled by [59]. The first, **Human-all**, is their comprehensive network aggregated from numerous sources, and the second is their high quality subnetwork **Human-hq**.

***D. melanogaster***. **Fly** combines all interactions in DroID [60] version 2011_02 with those from DPiM [8].

***A. thaliana***. **Athal** consists of protein-protein interactions obtained from IntAct [61], BioGRID, and from the supporting material of [9].

***E. coli***. **Ecoli** consists of protein-protein physical interactions extracted via the PSICQUIC View application [62].

### Network topology analysis

We briefly describe the topological measures that we utilize in our study.

The **degree** of a vertex is the number of interactions the corresponding protein has. In each network, we consider hubs to be proteins in the top 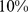 by degree, where the precise degree threshold to be called a hub is chosen such that at least 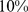 of vertices are hubs. These thresholds and the number of hubs for each network are shown in Table 1.

The **betweenness centrality** of a vertex 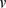 in a network is the number of shortest paths between all pairs of vertices in the network that pass through 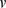, with the shortest paths between two genes 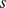 and 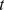 weighed inversely to the total number of distinct shortest paths between 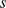 and 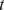.

The **clustering coefficient** of a node is defined as the ratio of the number of triangles containing that node to the number of triples centered on it; i.e., for a protein, this measures the number of interactions among its interactors, normalized by the maximum number of possible interactions.

The **participation coefficient** [37], [63] of a vertex with respect to a set of clusters in a network is defined as 
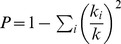
, where the summation is over all clusters, 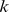 is the degree of the vertex, and 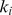 is the number of edges going from the vertex to vertices in cluster 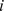. Note that 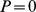 if all edges from a vertex go to a single cluster, and 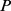 is closer to 1 if edges from the vertex are more uniformly distributed over clusters. To find clusters in the network, we used the SPICi clustering algorithm [64] with parameters optimized with a simple exhaustive search procedure to approximately maximize Newman's modularity measure [65]. See Section S2.4 in **Text S1** for details.

The **density** of a set of vertices 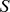 is the ratio of the actual number of edges between vertices in 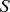 to the maximum possible such number 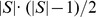. The neighborhood of a set of vertices 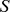 is the set of all vertices that are connected to some vertex from 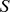 but are not themselves members of 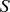. The **expansion** of a set of vertices 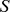 is the ratio 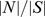, where 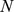 is the neighborhood of 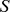. When measuring the density or expansion of a class of hubs, we compare it with the density or expansion of a random subset of the same number of background hubs as in the class in question. We consider 1000 independent samples, and report the empirical p-value of the actual value as compared to the distribution of random values.

The **average path length** for a network is measured as the average over all pairs of vertices of the lengths of the shortest paths between them. (For a disconnected network, only pairs of vertices connected by a path are considered.) The **relative size of the giant component** is calculated as the ratio of the size of the largest connected component in the network to the number of vertices in the network. The **global clustering coefficient** of a network measures the tendency of network vertices to cluster together. It is defined as thrice the number of triangles divided by the number of connected triples of vertices in the network.

In a **hub removal** experiment for a class of hubs, we remove all vertices of the class with their interactions from the network at once and measure the fold change of certain characteristics of the remaining network as compared with the initial network (e.g., if the average path length increased 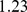 times, then the fold change is 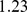). To compute an empirical p-value of this fold change value, we compare it with the distribution of the same values obtained after 1000 independent removals of random subsets of the same number of background hubs. We use the average path length, the size of the giant component, and the global clustering coefficient as global characteristics of network structure. By removing all hubs at once and comparing computed values with removals of random subsets of the same size, our hub removal experiment does not depend on the order in which hubs are removed or the size of the set of hubs considered, two issues which were raised previously [35], [36]. The results of these experiments can be compared for different classes of hubs, as in each case we compare the effect for a class of hubs relative to random subsets of the same size.

All topological measures are computed based on the python interface to the igraph library, version 0.5.4 (http://igraph.sourceforge.net/). We utilize degree-preserving network randomizations, as implemented in the igraph.Graph.Degree_Sequence( ) method with the “vl” option [40].

### Expression

Expression compendia for each organism consist of datasets collected from online databases and papers, as described in detail in Section S2.3 in the **Text S1** , and for each organism cover a wide range of conditions and/or tissue and cell types (where applicable). Each dataset is processed independently as follows: all replicates are merged (gene expression values averaged over replicates of the same experiment); genes with less than 50% known values are removed; the 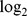-transformation is applied to all values if absolute signal values are given; for each matrix column corresponding to a single genome-wide experiment, the values of the column are transformed to z-scores.

For each organism, for each interacting pair of genes, we compute their co-expression via the Pearson correlation coefficient (PCC) of their expression profiles as follows. For genes with incomplete expression profiles within a dataset, only dimensions where values for both genes in the pair are known are used when computing the PCC of this pair. If the expression compendium for an organism consists of several datasets, the PCC is computed for each dataset independently, and then these PCC values are averaged with weights proportional to the number of expression datapoints that the dataset contributed to the compendium (in case of incomplete data, this is only over datasets where the PCC could be successfully computed), to obtain a final co-expression interaction score.

Some proteins in the networks are not included in any expression datasets. These proteins are not used to compute PCCs and avPCCs (see below), but may still contribute to degree or other topological properties of proteins. The number of interactions in the networks for which co-expression values are computed is shown in Table 1.

### Hub scores and classifications

For each hub, the average co-expression score (avPCC) is computed as the average of its co-expression interaction scores [29]. More precisely, the avPCC of a hub is the sum of all defined co-expression scores for interactions of the hub divided by hub degree (thus unknown edge scores are effectively assumed to be 0). Hubs are scored with avPCC only if they have at least 3 interactions with defined co-expression score.

Extremal hubs are defined as hubs in the top 5% by either degree or betweenness centrality amongst all hubs. For most networks, these two subsets of hubs are highly intersecting, so the union contains much less than 10% of all hubs. These hubs are excluded from the classification of hubs into date and party, and the corresponding analysis of this classification, but may still contribute to properties of other genes, particularly other hubs. Further, the background set of hubs, from which random sets of hubs are chosen to compute empirical p-values of several properties (as described above), does not include extremal hubs. Note, however, that extremal hubs are not excluded when doing correlation analysis of hub characteristics.

Party hubs are defined as the top one third by avPCC amongst all non-extremal hubs, and the remaining non-extremal hubs are defined as date hubs.

### Gene ontology analysis

For our functional analysis, we use Gene Ontology (GO) [39] terms and gene association data for each organism, not including associations with evidence codes IEA, RCA, IPI, ND or the qualifier NOT (downloaded from http://www.geneontology.org/ on July 25, 2011). The **functional similarity** of a pair of genes is computed as described in [37]. First, the information content of a term 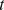 is defined as 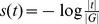, where 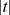 is the number of genes annotated with the term, and 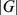 is the total number of genes in the organism annotated with at least one term. Then if 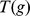 and 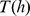 are the sets of terms annotating genes 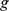 and 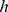 respectively, functional similarity is computed as 
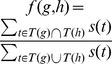
. For functional similarity, all GO Biological process terms of depth 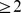 annotating at least 3 and at most 1000 genes are considered. The functional similarity of a vertex in a network is the average of functional similarity of this gene with all its interacting partners; proteins not annotated with one of the terms under consideration lead to functional similarities of 0.

We perform GO annotation enrichment test using the code of the project goatools (https://github.com/tanghaibao/goatools). We apply it to groups of hubs in the top and bottom one third or two thirds by avPCC, clustering, betweenness, participation and functional similarity in each network. For this analysis, we use broad functional terms that are direct children of roots of all three ontologies: biological process, 28 terms; cellular component, 13 terms; and molecular function, 20 terms. We use the set of all annotated hubs as the background population, and report terms with a Bonferroni-corrected p-value of less than 0.05. For each network, we test enrichment for each ontology (e.g., Biological process ontology) independently, and restrict the analysis only to the hub proteins that have at least some annotation with terms other than the root (e.g., Biological process) in this ontology.

### Essential genes

The 1222 essential genes for *S. cerevisiae* are obtained from the Saccharomyces Genome Deletion Project webpage (file http://www-sequence.stanford.edu/group/yeast_deletion_project/Essential_ORFs.txt).

### Orthologs

We use protein ortholog information from version 4 of the Princeton Protein Orthology Database (P-POD) [43] (ftp://gen-ftp.princeton.edu/ppod/). We consider two proteins in different networks to be orthologous if they are categorized in the same family by P-POD using either OrthoMCL or MultiParanoid.

For each pair of networks for two different organisms, we consider each pair of hubs 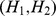 where 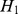 and 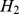 are non-extremal hubs in the networks of organisms 1 and 2, respectively, that are reported to be orthologous. A hub can appear in several pairs if it has more than one ortholog in another species. The Spearman correlation coefficient is computed over hub pairs for various network characteristics (avPCC, clustering coefficient, etc.).

Since a hub may contribute to several pairs of orthologs, in addition to using the standard computation of the p-value for the Spearman correlation coefficient, we also calculate an empirical p-value in the following way: the actual Spearman's rho is compared with the distribution of Spearman's rho values calculated in exactly the same manner as above, but for the characteristic (say, avPCC) among hubs randomly permuted in each of the two networks (as opposed to permuting vector components that contain repetitions). We report the empirical p-value of the actual correlation with respect to the distribution of correlations from 1000 instances of randomized data.

We test if the low range of a hub feature (say, avPCC) is evolutionary conserved as much as the high range. For two networks of different organisms, we extract hubs in the top one third and bottom one third as ranked by the feature computed in each network. We calculate for each pair of hub groups (top third from the first organism vs. top third from the second organism, top third from the first organism vs. bottom third from the second organism, etc.) how many ortholog pairs are observed between them. Then we compare this number with the number calculated in exactly the same manner, but for 1000 random samples of the same number of hubs in each network, and report the corresponding z-score and empirical p-value of the actual number of orthologs compared with the distribution of the numbers for random data.

## Supporting Information

Figure S1
**Date and party hub classification analysis in yeast high quality network (Yeast-hq).** (A) Number of hubs in each class. Party hubs in this network have avPCC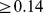; this threshold corresponds to the top third of avPCC values for all hubs categorized as either party or date. (B) Betweenness, clustering coefficient, participation coefficient and functional similarity for date and party hubs. (C) Density and expansion of date and party hubs. (D) Effect of hub removal for party and date when considering the average path distance, the size of the largest connected component, and the global clustering coefficient. See caption of Fig. 1 in the main text for details.(TIF)

Figure S2
**Date and party hub classification analysis in fly network of all physical interactions (Fly).** (A) Number of hubs in each class. Party hubs in this network have avPCC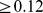; this threshold corresponds to the top third of avPCC values for all hubs categorized as either party or date. (B) Betweenness, clustering coefficient, participation coefficient and functional similarity for date and party hubs. (C) Density and expansion of date and party hubs. (D) Effect of hub removal for party and date when considering the average path distance, the size of the largest connected component, and the global clustering coefficient. See caption of Fig. 1 in the main text for details.(TIF)

Figure S3
**Date and party hub classification analysis in Arabidopsis network (Athal).** (A) Number of hubs in each class. Party hubs in this network have avPCC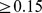; this threshold corresponds to the top third of avPCC values for all hubs categorized as either party or date. (B) Betweenness, clustering coefficient, participation coefficient and functional similarity for date and party hubs. (C) Density and expansion of date and party hubs. (D) Effect of hub removal for party and date when considering the average path distance, the size of the largest connected component, and the global clustering coefficient. See caption of Fig. 1 in the main text for details.(TIF)

Figure S4
**Date and party hub classification analysis in** ***E. coli*** **network ( Ecoli ).** (A) Number of hubs in each class. Party hubs in this network have avPCC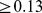; this threshold corresponds to the top third of avPCC values for all hubs categorized as either party or date. (B) Betweenness, clustering coefficient, participation coefficient and functional similarity for date and party hubs. (C) Density and expansion of date and party hubs. (D) Effect of hub removal for party and date when considering the average path distance, the size of the largest connected component, and the global clustering coefficient. See caption of Fig. 1 in the main text for details.(TIF)

Figure S5
**Date and party hub classification analysis in human network of all physical interactions (Human-all).** (A) Number of hubs in each class. Party hubs in this network have avPCC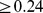; this threshold corresponds to the top third of avPCC values for all hubs categorized as either party or date. (B) Betweenness, clustering coefficient, participation coefficient and functional similarity for date and party hubs. (C) Density and expansion of date and party hubs. (D) Effect of hub removal for party and date when considering the average path distance, the size of the largest connected component, and the global clustering coefficient. See caption of Fig. 1 in the main text for details.(TIF)

Figure S6
**Date and party hub classification analysis in yeast network of all physical interactions (Yeast-all).** (A) Number of hubs in each class. Party hubs in this network have avPCC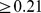; this threshold corresponds to the top third of avPCC values for all hubs categorized as either party or date. (B) Betweenness, clustering coefficient, participation coefficient and functional similarity for date and party hubs. (C) Density and expansion of date and party hubs. (D) Effect of hub removal for party and date when considering the average path distance, the size of the largest connected component, and the global clustering coefficient. See caption of Fig. 1 in the main text for details.(TIF)

Figure S7
**Date and party hub classification analysis in human high quality network (Human-hq) with extremal hubs included.** (A) Number of hubs in each class. Party hubs in this network have avPCC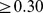; this threshold corresponds to the top third of avPCC values for all hubs categorized as either party or date. (B) Betweenness, clustering coefficient, participation coefficient and functional similarity for date and party hubs. (C) Density and expansion of date and party hubs. (D) Effect of hub removal for party and date when considering the average path distance, the size of the largest connected component, and the global clustering coefficient. See caption of Fig. 1 in the main text for details.(TIF)

Figure S8
**Date and party hub classification analysis in human network of all physical interactions ( Human-all ), with all genes of degree** 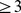 **as hubs.** (A) Number of hubs in each class. Party hubs in this network have avPCC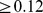; this threshold corresponds to the top third of avPCC values for all hubs categorized as either party or date. (B) Betweenness, clustering coefficient, participation coefficient and functional similarity for date and party hubs. (C) Density and expansion of date and party hubs. (D) Effect of hub removal for party and date when considering the average path distance, the size of the largest connected component, and the global clustering coefficient. See caption of Fig. 1 in the main text for details.(TIF)

Figure S9
**Date and party hub classification analysis in yeast network of all physical interactions ( Yeast-all ), with all genes of degree** 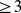 **as hubs.** (A) Number of hubs in each class. Party hubs in this network have avPCC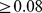; this threshold corresponds to the top third of avPCC values for all hubs categorized as either party or date. (B) Betweenness, clustering coefficient, participation coefficient and functional similarity for date and party hubs. (C) Density and expansion of date and party hubs. (D) Effect of hub removal for party and date when considering the average path distance, the size of the largest connected component, and the global clustering coefficient. See caption of Fig. 1 in the main text for details.(TIF)

Figure S10
**Date and party hub classification analysis in fly network of all physical interactions ( Fly ), with all genes of degree** 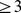 **as hubs.** (A) Number of hubs in each class. Party hubs in this network have avPCC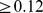; this threshold corresponds to the top third of avPCC values for all hubs categorized as either party or date. (B) Betweenness, clustering coefficient, participation coefficient and functional similarity for date and party hubs. (C) Density and expansion of date and party hubs. (D) Effect of hub removal for party and date when considering the average path distance, the size of the largest connected component, and the global clustering coefficient. See caption of Fig. 1 in the main text for details.(TIF)

Figure S11
**Spearman correlation of hub characteristics in interaction networks, with all genes of degree** 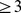 **as hubs.** Every bar represents a Spearman correlation between two characteristics of hubs in one of the networks. Bars of significant correlations (absolute value 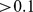, p-value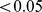) have black edges. Smaller uncolored bars show average correlation (with error bars depicting the standard deviations) in 20 random networks on the same genes with the same number of interactions for each.(TIF)

Figure S12
**Spearman correlation of hub characteristics in interaction networks, with all genes of degree** 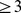 **as hubs and with correction for degree.** Every bar represents a partial Spearman correlation corrected for degree between two characteristics of hubs in one of the networks. Bars of significant correlations (absolute value 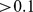, p-value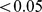) have black edges. Smaller uncolored bars show average correlation (with error bars depicting the standard deviations) in 20 random networks on the same genes with the same number of interactions for each.(TIF)

Figure S13
**Correlation with degree is not a confounding factor in the correlation analysis of hub characteristics.** (A) Every bar represents a Spearman correlation between a hub characteristic and degree (the number of interactions) for hubs in one of the networks. (B) Every bar represents a partial Spearman correlation corrected for degree between two characteristics of hubs in one of the networks. Bars of significant correlations (absolute value 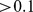, p-value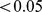) have black edges. Smaller uncolored bars show average correlation (with error bars for standard deviations) in 20 random networks on the same genes with the same number of interactions for each.(TIF)

Figure S14
**Hubs with extremal properties do not bias the correlation analysis of hub characteristics.** Every bar represents a Spearman correlation between two characteristics of non-extremal hubs in one of the networks. Bars of significant correlations (absolute value 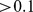, p-value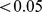) have black edges. Smaller uncolored bars show average correlation (with error bars for standard deviations) in 20 random networks on the same genes with the same number of interactions for each.(TIF)

Figure S15
**Spearman correlation of hub characteristics in high-throughput interaction networks for human and yeast.** Every bar represents a Spearman correlation between two characteristics of hubs in one of the networks. Bars of significant correlations (absolute value 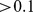, p-value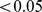) have black edges. Smaller uncolored bars show average correlation (with error bars for standard deviations) in 20 random networks on the same genes with the same number of interactions for each.(TIF)

Figure S16 **GO annotation enrichment analysis of hubs in Yeast-hq.** GO annotation enrichment analysis of hubs divided in a 2-to-1 proportion by avPCC, clustering, betweenness, participation and functional similarity scores in **Yeast-hq**. See Fig. 3 in the main text for details.
(TIF)

Figure S17 **GO annotation enrichment analysis of hubs in Human-all.** GO annotation enrichment analysis of hubs divided in a 2-to-1 proportion by avPCC, clustering, betweenness, participation and functional similarity scores in **Human-all**. See Fig. 3 in the main text for details.
(TIF)

Figure S18 **GO annotation enrichment analysis of hubs in Yeast-all.** GO annotation enrichment analysis of hubs divided in a 2-to-1 proportion by avPCC, clustering, betweenness, participation and functional similarity scores in **Yeast-all**. See Fig. 3 in the main text for details.
(TIF)

Figure S19 **GO annotation enrichment analysis of hubs in Fly.** GO annotation enrichment analysis of hubs divided in a 2-to-1 proportion by avPCC, clustering, betweenness, participation and functional similarity scores in **Fly**. See Fig. 3 in the main text for details.
(TIF)

Figure S20 **GO annotation enrichment analysis of hubs in Athal.** GO annotation enrichment analysis of hubs divided in a 2-to-1 proportion by avPCC, clustering, betweenness, participation and functional similarity scores in **Athal**. See Fig. 3 in the main text for details.
(TIF)

Figure S21 **GO annotation enrichment analysis of hubs in Ecoli.** GO annotation enrichment analysis of hubs divided in a 2-to-1 proportion by avPCC, clustering, betweenness, participation and functional similarity scores in **Ecoli**. See Fig. 3 in the main text for details.
(TIF)

Figure S22 **Genetic interactions for date and party hubs in yeast.** Date hubs participate in significantly larger number of genetic interactions than party hubs, when date and party hubs are defined from yeast networks (A) **Yeast-hq** (B) **Yeast-all** (Mann–Whitney U). Even when all essential genes are removed from consideration, the same trend is observed for both networks (C) **Yeast-hq** and (D) **Yeast-all**.
(TIF)

Figure S23
**Spearman correlation of hub characteristics with the number of negative and positive genetic interactions.** (A) Every bar represents a Spearman correlation between a hub characteristic and the number of negative genetic interactions for hubs in one of the physical interaction networks for yeast. (B) Every bar represents a Spearman correlation between a hub characteristic and the number of positive genetic interactions for hubs in one of the physical interaction networks for yeast. Bars of significant correlations (absolute value 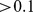, p-value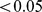) have black edges.(TIF)

Figure S24
**Essentiality is not a confounding factor in the correlation analysis of genetic degree with hub characteristics in yeast physical interaction networks.** (A) Every bar represents a Spearman correlation between essentiality (1 if essential, 0 otherwise) and the number of genetic interactions for hubs in one of the physical interaction networks for yeast. (B) Every bar represents a partial Spearman correlation between a hub characteristic and the number of genetic interactions corrected for essentiality for hubs in one of the physical interaction networks for yeast. Bars of significant correlations (absolute value 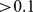, p-value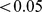) have black edges.(TIF)

Figure S25
**Spearman correlation of hub characteristics in yeast two-hybrid and co-complex interaction networks.** Every bar represents a Spearman correlation between two characteristics of hubs in one of the networks. Bars of significant correlations (absolute value 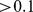, p-value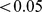) have black edges. Smaller uncolored bars show average correlation (with error bars for standard deviations) in 20 random networks on the same genes with the same number of interactions for each.(TIF)

Figure S26
**Date and party hub classification analysis in human network of all known interactions from yeast two-hybrid experiments (Human-all-y2h).** (A) Number of hubs in each class. Party hubs in this network have avPCC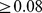; this threshold corresponds to the top third of avPCC values for all hubs categorized as either party or date. (B) Betweenness, clustering coefficient, participation coefficient and functional similarity for date and party hubs. (C) Density and expansion of date and party hubs. (D) Effect of hub removal for party and date when considering the average path distance, the size of the largest connected component, and the global clustering coefficient. See caption of Fig. 1 in the main text for details.(TIF)

Figure S27
**Date and party hub classification analysis in human network of all known interactions derived from complexes (Human-all-cocompl).** (A) Number of hubs in each class. Party hubs in this network have avPCC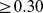; this threshold corresponds to the top third of avPCC values for all hubs categorized as either party or date. (B) Betweenness, clustering coefficient, participation coefficient and functional similarity for date and party hubs. (C) Density and expansion of date and party hubs. (D) Effect of hub removal for party and date when considering the average path distance, the size of the largest connected component, and the global clustering coefficient. See caption of Fig. 1 in the main text for details.(TIF)

Figure S28
**Date and party hub classification analysis in yeast network of all known interactions from yeast two-hybrid experiments (Yeast-all-y2h).** (A) Number of hubs in each class. Party hubs in this network have avPCC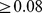; this threshold corresponds to the top third of avPCC values for all hubs categorized as either party or date. (B) Betweenness, clustering coefficient, participation coefficient and functional similarity for date and party hubs. (C) Density and expansion of date and party hubs. (D) Effect of hub removal for party and date when considering the average path distance, the size of the largest connected component, and the global clustering coefficient. See caption of Fig. 1 in the main text for details.(TIF)

Figure S29
**Date and party hub classification analysis in the yeast network of all known interactions derived from complexes (Yeast-all-cocompl).** (A) Number of hubs in each class. Party hubs in this network have avPCC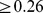; this threshold corresponds to the top third of avPCC values for all hubs categorized as either party or date. (B) Betweenness, clustering coefficient, participation coefficient and functional similarity for date and party hubs. (C) Density and expansion of date and party hubs. (D) Effect of hub removal for party and date when considering the average path distance, the size of the largest connected component, and the global clustering coefficient. See caption of Fig. 1 in the main text for details.(TIF)

Figure S30
**Date and party hub classification analysis in the Arabidopsis network of all known interactions from yeast two-hybrid experiments (Athal-y2h).** (A) Number of hubs in each class. Party hubs in this network have avPCC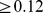; this threshold corresponds to the top third of avPCC values for all hubs categorized as either party or date. (B) Betweenness, clustering coefficient, participation coefficient and functional similarity for date and party hubs. (C) Density and expansion of date and party hubs. (D) Effect of hub removal for party and date when considering the average path distance, the size of the largest connected component, and the global clustering coefficient. See caption of Fig. 1 in the main text for details.(TIF)

Figure S31
**Date and party hub classification analysis in Arabidopsis network of all known interactions derived from complexes (Athal-cocompl).** (A) Number of hubs in each class. Party hubs in this network have avPCC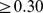; this threshold corresponds to the top third of avPCC values for all hubs categorized as either party or date. (B) Betweenness, clustering coefficient, participation coefficient and functional similarity for date and party hubs. (C) Density and expansion of date and party hubs. (D) Effect of hub removal for party and date when considering the average path distance, the size of the largest connected component, and the global clustering coefficient. See caption of Fig. 1 in the main text for details.(TIF)

Figure S32 **Yeast two-hybrid and co-complex interactions of date and party hubs.** Date hubs have significantly many more binary (yeast two-hybrid, y2h) interactions, while party hubs participate in significantly larger number of interactions derived from complexes (co-complex, cocompl) in networks (A) **Human-all** (B) **Yeast-all** (C) **Athal** (Mann–Whitney U).
(TIF)

Figure S33 **Party hubs are more likely to be essential than date hubs.** Fraction of date and party hubs that are essential in (A) **Yeast-hq** (B) **Yeast-all**. Party hubs are significantly enriched with essential genes (hypergeometric test). Spearman correlation (with p-value) of essentiality indicator vector (1 if essential, 0 otherwise) and avPCC shown on bottom is significantly positive.
(TIF)

Figure S34
**avPCC-rand is not a confounding factor in the correlation analysis of hub characteristics and genetic degree in yeast physical interaction networks.** (A) Every bar represents a Spearman correlation between a hub characteristic and the number of genetic interactions for hubs in one of the yeast networks. Bars of significant correlations (absolute value 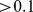, p-value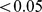) have black edges. (B) Every bar represents a partial Spearman correlation between a hub characteristic and the number of genetic interactions corrected for avPCC-rand for hubs in one of the yeast networks. Smaller uncolored bars show average correlation (with error bars for standard deviations) in 100 random networks on the same genes with the same number of interactions for each. Random networks used for the plot are different from those used for the calculation of avPCC-rand.(TIF)

Figure S35
**avPCC-rand is not a confounding factor in the correlation analysis of hub characteristics and essentiality in yeast physical interaction networks.** (A) Every bar represents a Spearman correlation between a hub characteristic and essentiality (1 if essential, 0 otherwise) for hubs in one of the yeast networks. (B) Every bar represents a partial Spearman correlation between a hub characteristic and essentiality (1 if essential, 0 otherwise) corrected for avPCC-rand for hubs in one of the yeast networks. Bars of significant correlations (absolute value 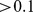, p-value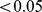) have black edges. Smaller uncolored bars show average correlation (with error bars for standard deviations) in 100 random networks on the same genes with the same number of interactions for each. Random networks used for the plot are different from those used for the calculation of avPCC-rand.(TIF)

Table S1 **Spearman correlation of avPCC with clustering, betweenness, participation and functional similarity of hubs in the network.**
(PDF)

Table S2 **Spearman correlation of clustering coefficient with betweenness, participation and functional similarity of hubs in the network.**
(PDF)

Table S3 **Spearman correlation of betweenness centrality with participation and functional similarity of hubs in the network.**
(PDF)

Table S4 **Spearman correlation of participation coefficient with functional similarity.**
(PDF)

Table S5 **Spearman correlation for characteristics of orthologous hubs in Yeast-hq and Human-hq .**
(PDF)

Table S6 **Spearman correlation of avPCC for orthologs between species.**
(PDF)

Table S7 **Spearman correlation of clustering coefficient for orthologs between species.**
(PDF)

Table S8 **Spearman correlation of betweenness centrality for orthologs between species.**
(PDF)

Table S9 **Spearman correlation of participation coefficient for orthologs between species.**
(PDF)

Table S10 **Spearman correlation of functional similarity for orthologs between species.**
(PDF)

Table S11 **Fraction of hubs annotated with GO terms in each network.**
(PDF)

Table S12 **Interaction evidence types from different sources used for interaction annotation.**
(PDF)

Table S13 **Datasets used in** ***S. cerevisiae*** **expression compendium.**
(PDF)

Table S14 **Datasets used in** ***D. melanogaster*** **expression compendium.**
(PDF)

Text S1 **Simple topological features reflect dynamics and modularity in protein interaction networks.**
(PDF)
