# Supplementary figures and images for: Simple Topological Features Reflect Dynamics and Modularity in Protein Interaction Networks
Source: PLoS Comput Biol. 2013 Oct 10;9(10):e1003243. doi: 10.1371/journal.pcbi.1003243 (PMC3794914; doi:10.1371/journal.pcbi.1003243)

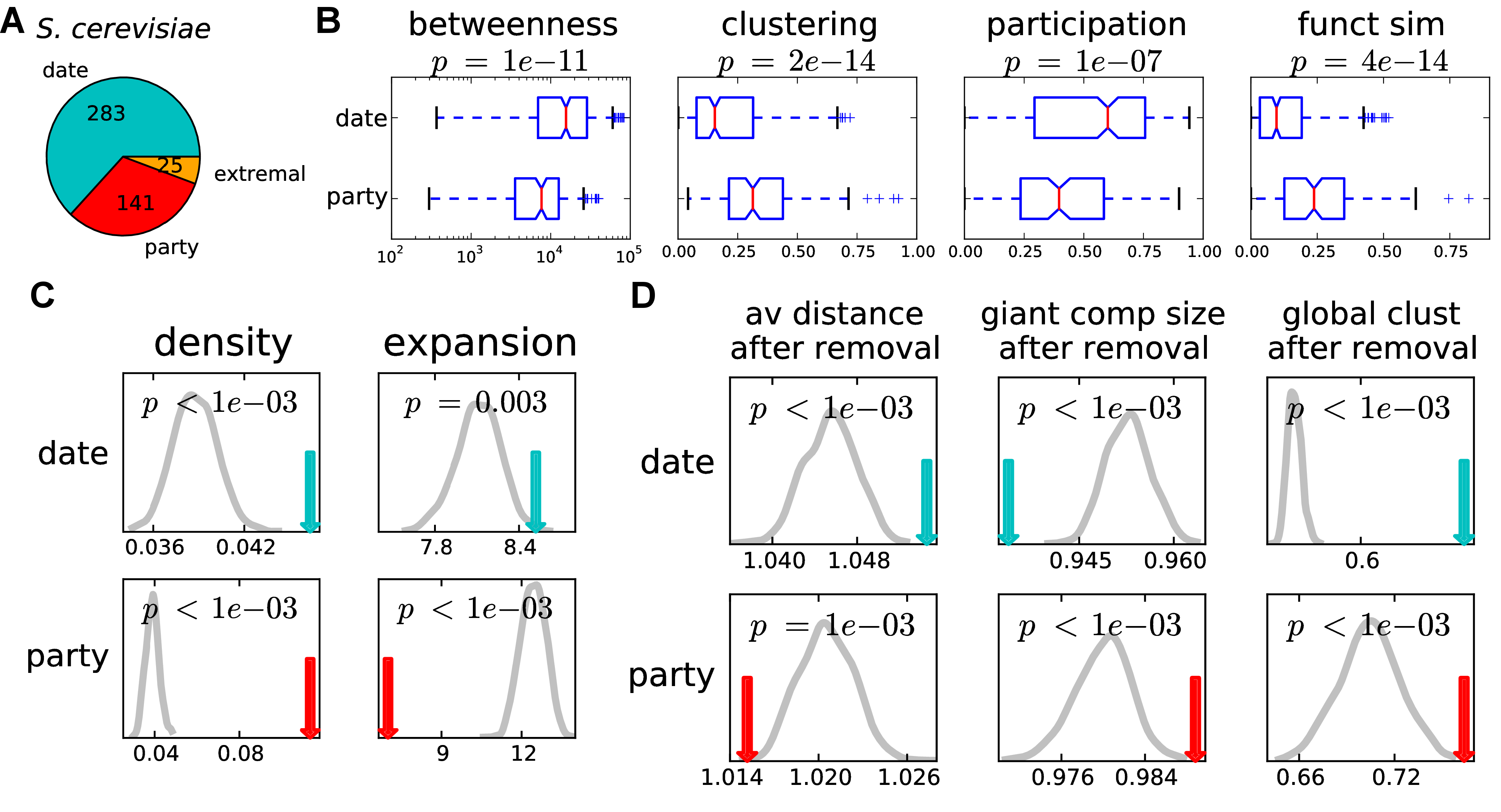

Supplement: Figure S1 — Date and party hub classification analysis in yeast high quality network (Yeast-hq). (A) Number of hubs in each class. Party hubs in this network have avPCC; this threshold corresponds to the top third of avPCC values for all hubs categorized as either party or date. (B) Betweenness, clustering coefficient, participation coefficient and functional similarity for date and party hubs. (C) Density and expansion of date and party hubs. (D) Effect of hub removal for party and date when considering the average path distance, the size of the largest connected component, and the global clustering coefficient. See caption of Fig. 1 in the main text for details. (TIF) [file pcbi.1003243.s001.tif]

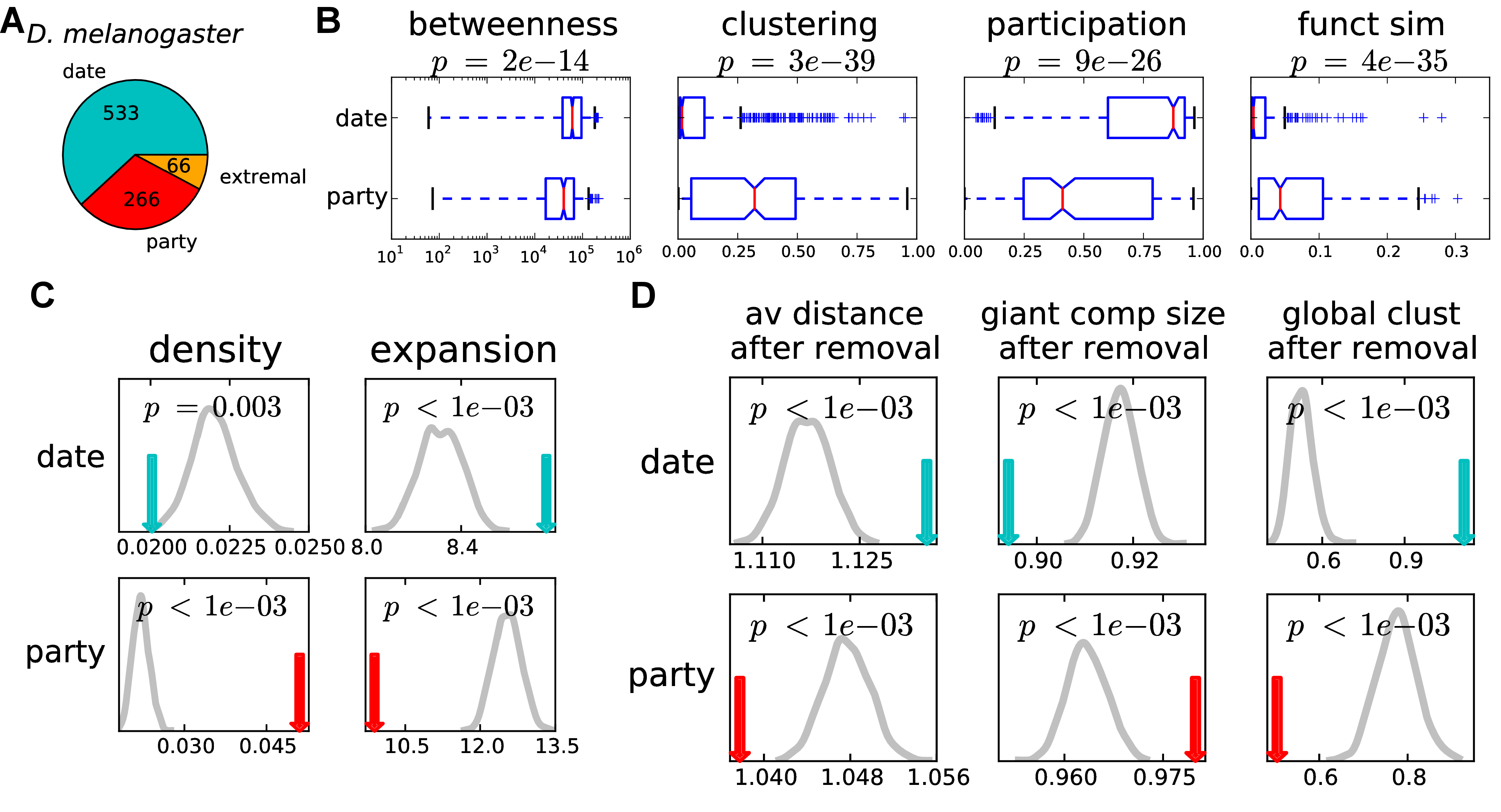

Supplement: Figure S2 — Date and party hub classification analysis in fly network of all physical interactions (Fly). (A) Number of hubs in each class. Party hubs in this network have avPCC; this threshold corresponds to the top third of avPCC values for all hubs categorized as either party or date. (B) Betweenness, clustering coefficient, participation coefficient and functional similarity for date and party hubs. (C) Density and expansion of date and party hubs. (D) Effect of hub removal for party and date when considering the average path distance, the size of the largest connected component, and the global clustering coefficient. See caption of Fig. 1 in the main text for details. (TIF) [file pcbi.1003243.s002.tif]

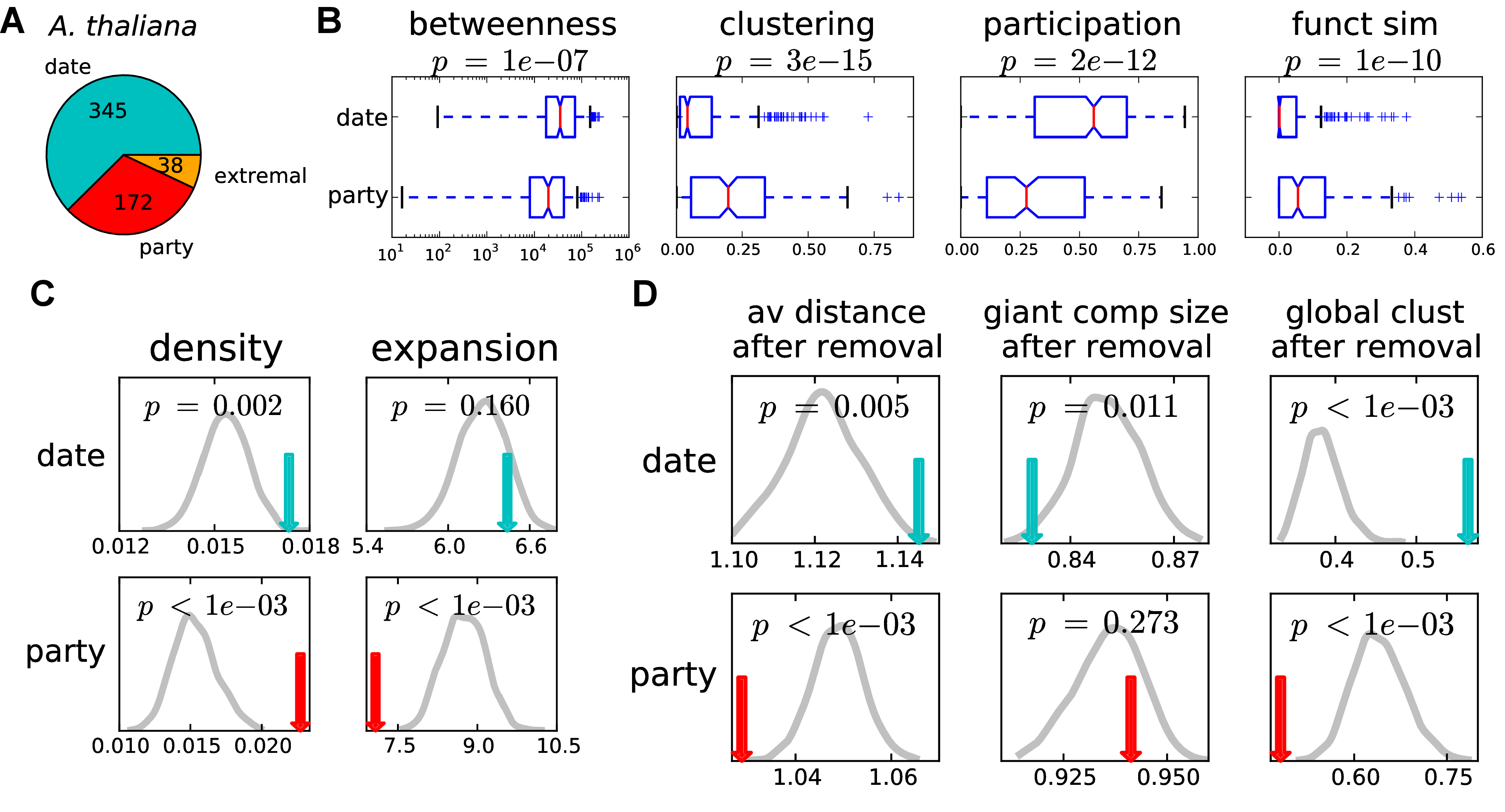

Supplement: Figure S3 — Date and party hub classification analysis in Arabidopsis network (Athal). (A) Number of hubs in each class. Party hubs in this network have avPCC; this threshold corresponds to the top third of avPCC values for all hubs categorized as either party or date. (B) Betweenness, clustering coefficient, participation coefficient and functional similarity for date and party hubs. (C) Density and expansion of date and party hubs. (D) Effect of hub removal for party and date when considering the average path distance, the size of the largest connected component, and the global clustering coefficient. See caption of Fig. 1 in the main text for details. (TIF) [file pcbi.1003243.s003.tif]

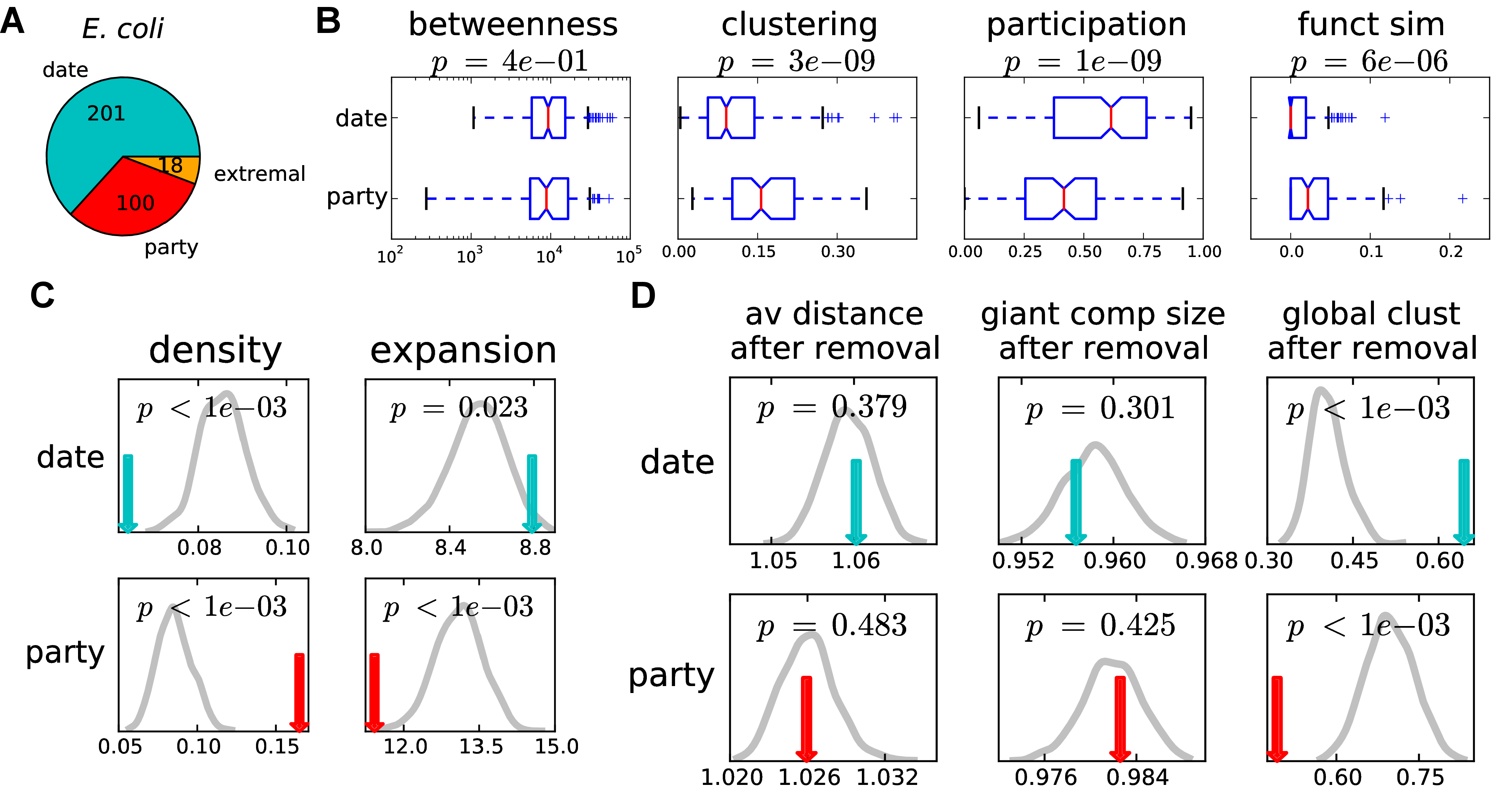

Supplement: Figure S4 — Date and party hub classification analysis in E. coli network ( Ecoli ). (A) Number of hubs in each class. Party hubs in this network have avPCC; this threshold corresponds to the top third of avPCC values for all hubs categorized as either party or date. (B) Betweenness, clustering coefficient, participation coefficient and functional similarity for date and party hubs. (C) Density and expansion of date and party hubs. (D) Effect of hub removal for party and date when considering the average path distance, the size of the largest connected component, and the global clustering coefficient. See caption of Fig. 1 in the main text for details. (TIF) [file pcbi.1003243.s004.tif]

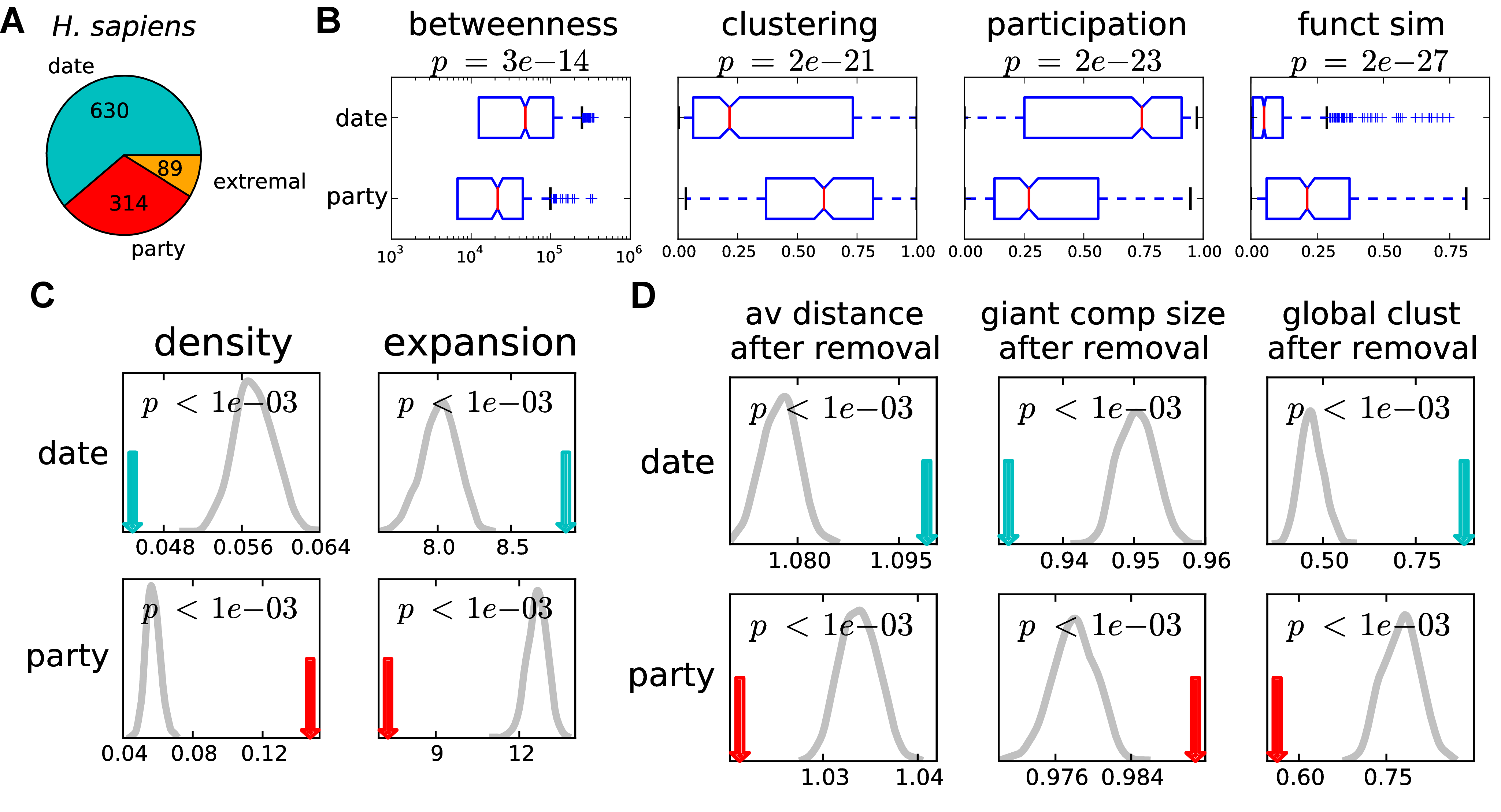

Supplement: Figure S5 — Date and party hub classification analysis in human network of all physical interactions (Human-all). (A) Number of hubs in each class. Party hubs in this network have avPCC; this threshold corresponds to the top third of avPCC values for all hubs categorized as either party or date. (B) Betweenness, clustering coefficient, participation coefficient and functional similarity for date and party hubs. (C) Density and expansion of date and party hubs. (D) Effect of hub removal for party and date when considering the average path distance, the size of the largest connected component, and the global clustering coefficient. See caption of Fig. 1 in the main text for details. (TIF) [file pcbi.1003243.s005.tif]

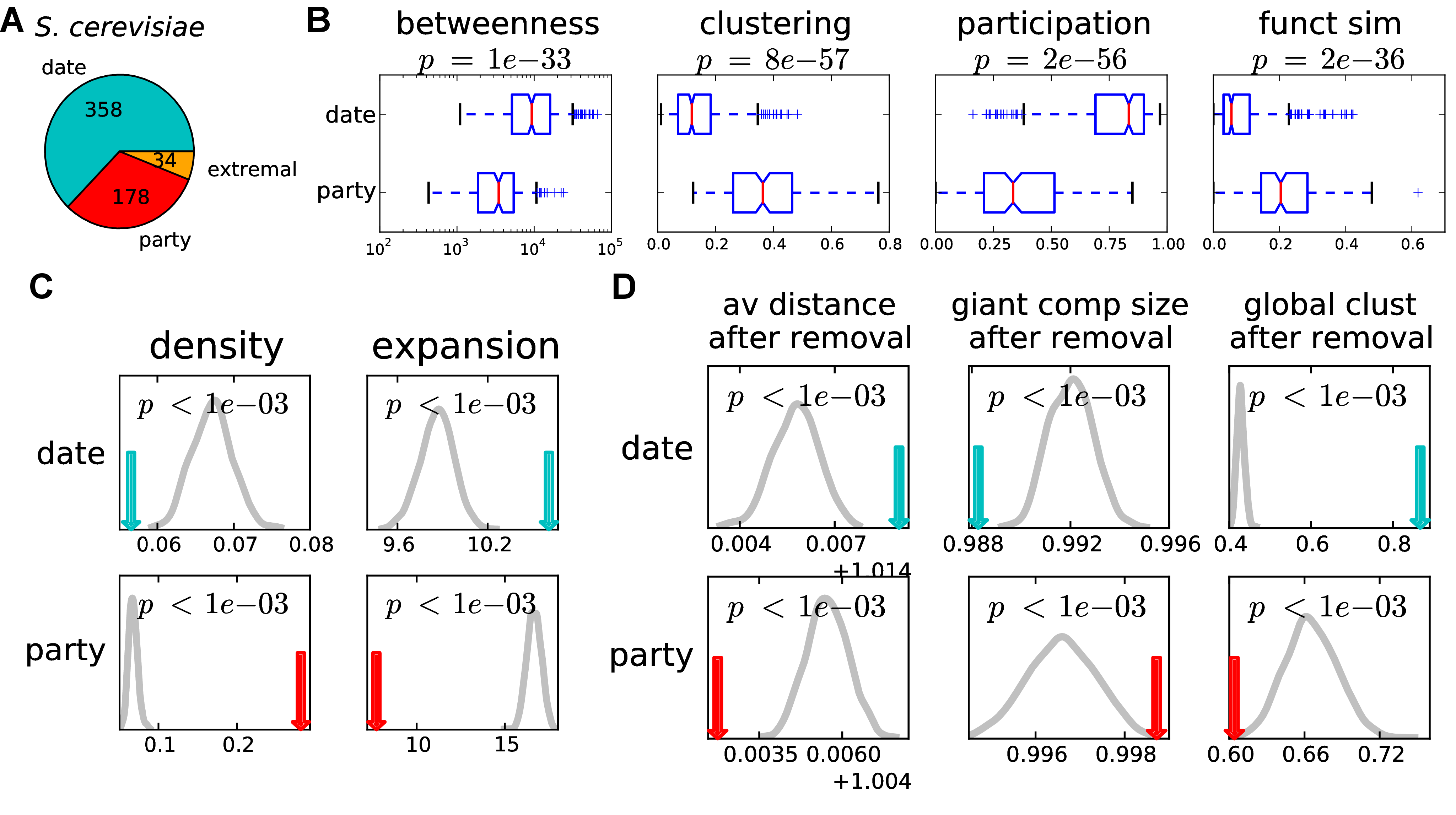

Supplement: Figure S6 — Date and party hub classification analysis in yeast network of all physical interactions (Yeast-all). (A) Number of hubs in each class. Party hubs in this network have avPCC; this threshold corresponds to the top third of avPCC values for all hubs categorized as either party or date. (B) Betweenness, clustering coefficient, participation coefficient and functional similarity for date and party hubs. (C) Density and expansion of date and party hubs. (D) Effect of hub removal for party and date when considering the average path distance, the size of the largest connected component, and the global clustering coefficient. See caption of Fig. 1 in the main text for details. (TIF) [file pcbi.1003243.s006.tif]

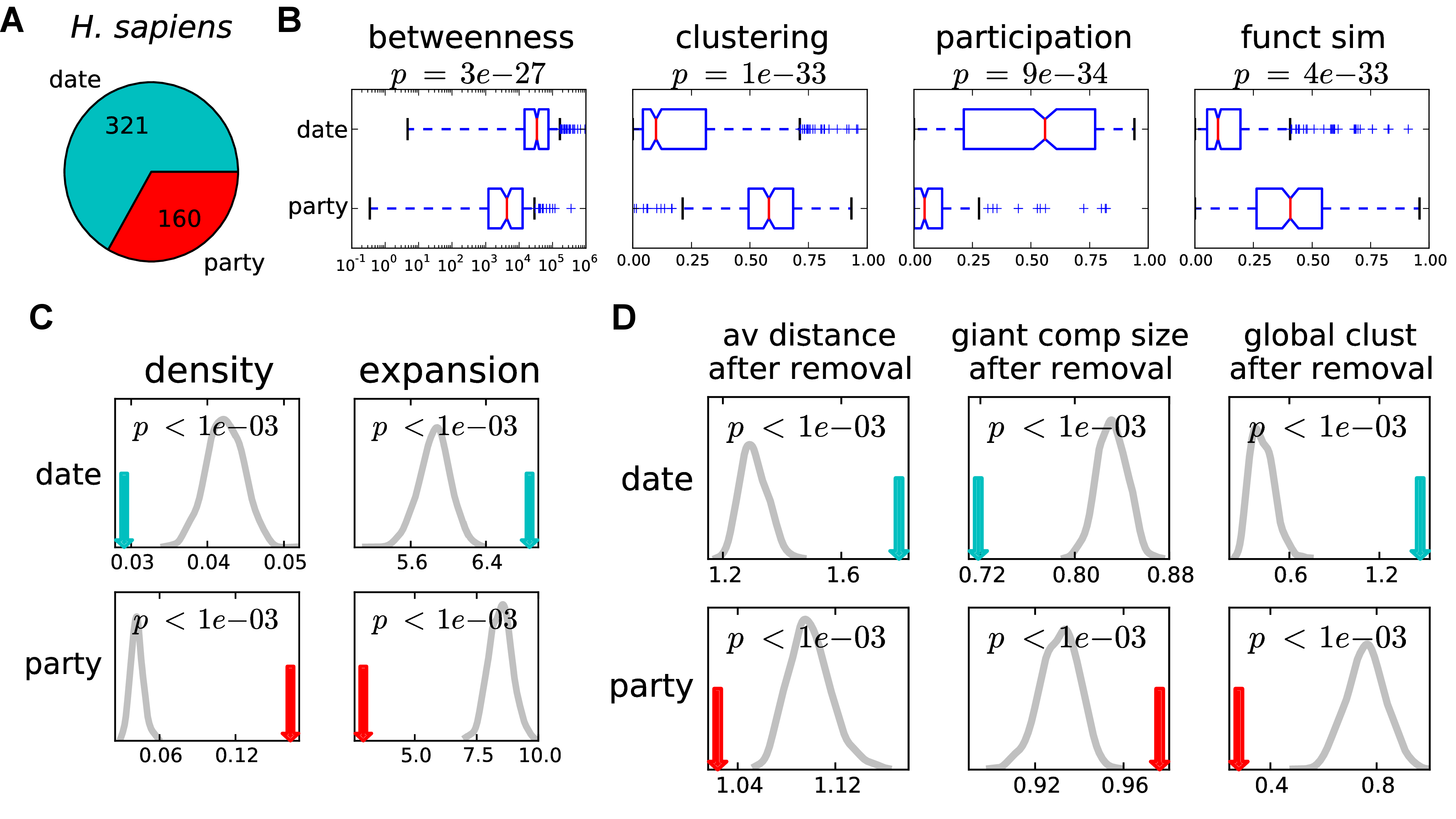

Supplement: Figure S7 — Date and party hub classification analysis in human high quality network (Human-hq) with extremal hubs included. (A) Number of hubs in each class. Party hubs in this network have avPCC; this threshold corresponds to the top third of avPCC values for all hubs categorized as either party or date. (B) Betweenness, clustering coefficient, participation coefficient and functional similarity for date and party hubs. (C) Density and expansion of date and party hubs. (D) Effect of hub removal for party and date when considering the average path distance, the size of the largest connected component, and the global clustering coefficient. See caption of Fig. 1 in the main text for details. (TIF) [file pcbi.1003243.s007.tif]

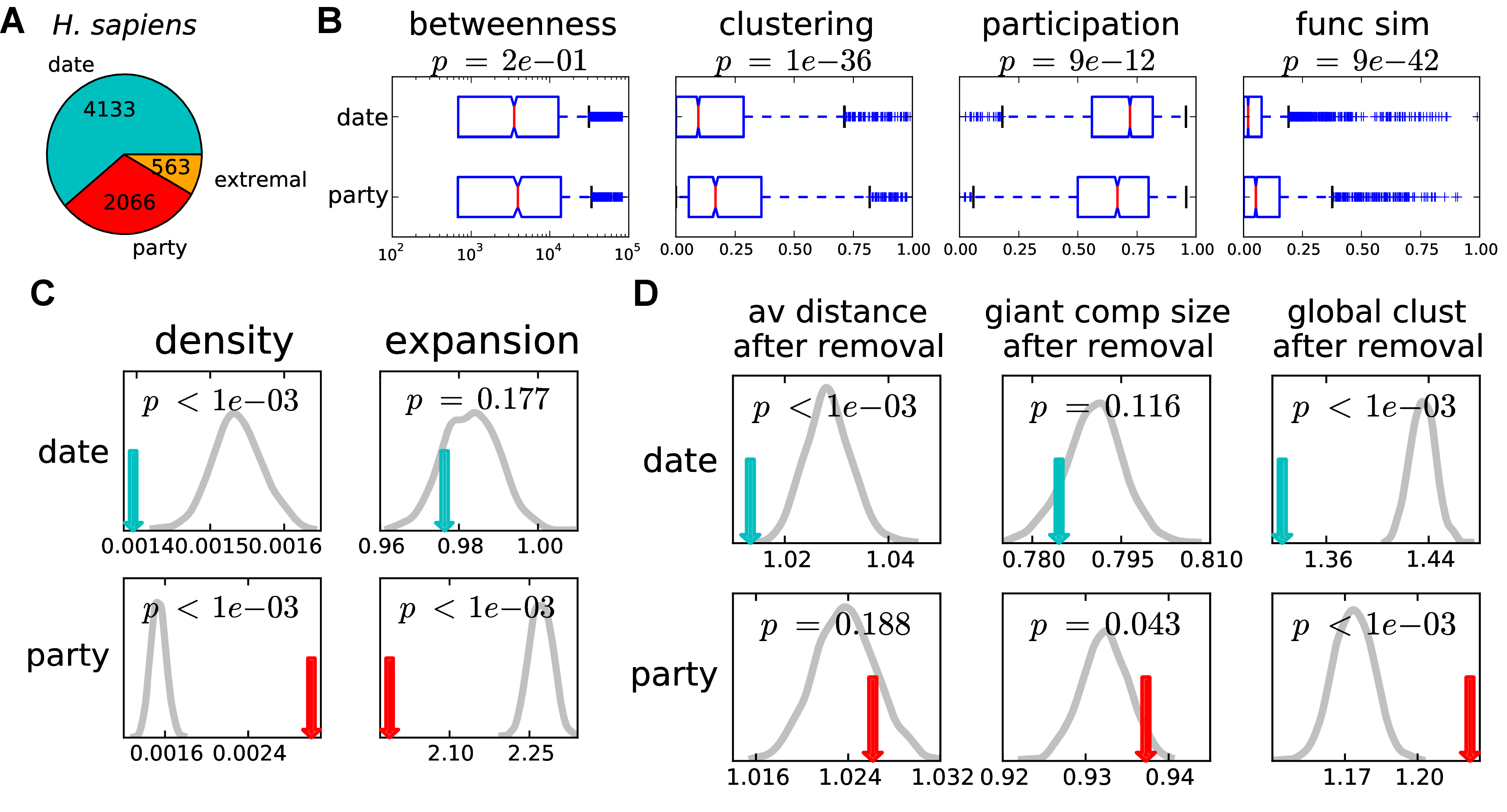

Supplement: Figure S8 — Date and party hub classification analysis in human network of all physical interactions ( Human-all ), with all genes of degree as hubs. (A) Number of hubs in each class. Party hubs in this network have avPCC; this threshold corresponds to the top third of avPCC values for all hubs categorized as either party or date. (B) Betweenness, clustering coefficient, participation coefficient and functional similarity for date and party hubs. (C) Density and expansion of date and party hubs. (D) Effect of hub removal for party and date when considering the average path distance, the size of the largest connected component, and the global clustering coefficient. See caption of Fig. 1 in the main text for details. (TIF) [file pcbi.1003243.s008.tif]

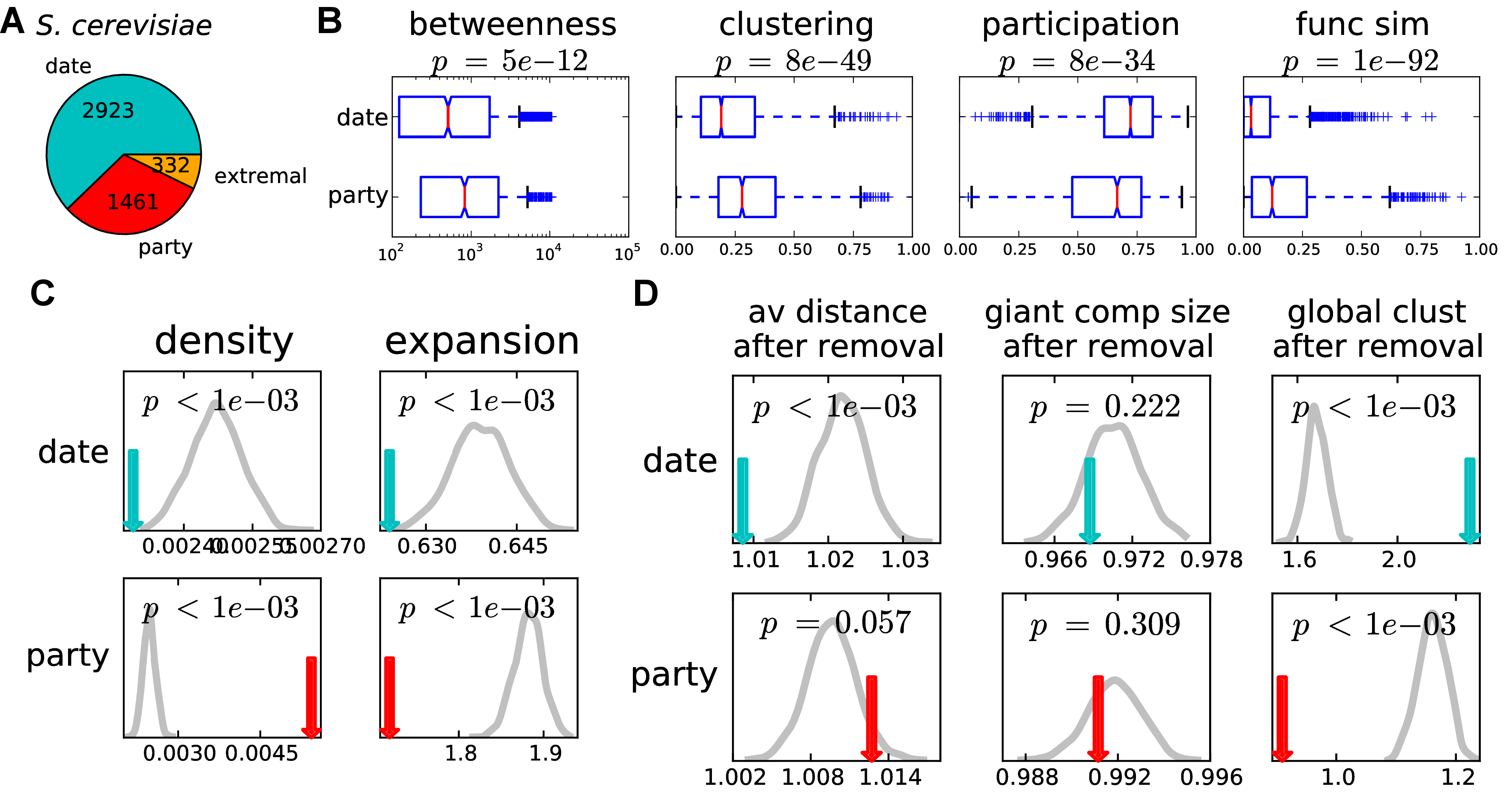

Supplement: Figure S9 — Date and party hub classification analysis in yeast network of all physical interactions ( Yeast-all ), with all genes of degree as hubs. (A) Number of hubs in each class. Party hubs in this network have avPCC; this threshold corresponds to the top third of avPCC values for all hubs categorized as either party or date. (B) Betweenness, clustering coefficient, participation coefficient and functional similarity for date and party hubs. (C) Density and expansion of date and party hubs. (D) Effect of hub removal for party and date when considering the average path distance, the size of the largest connected component, and the global clustering coefficient. See caption of Fig. 1 in the main text for details. (TIF) [file pcbi.1003243.s009.tif]

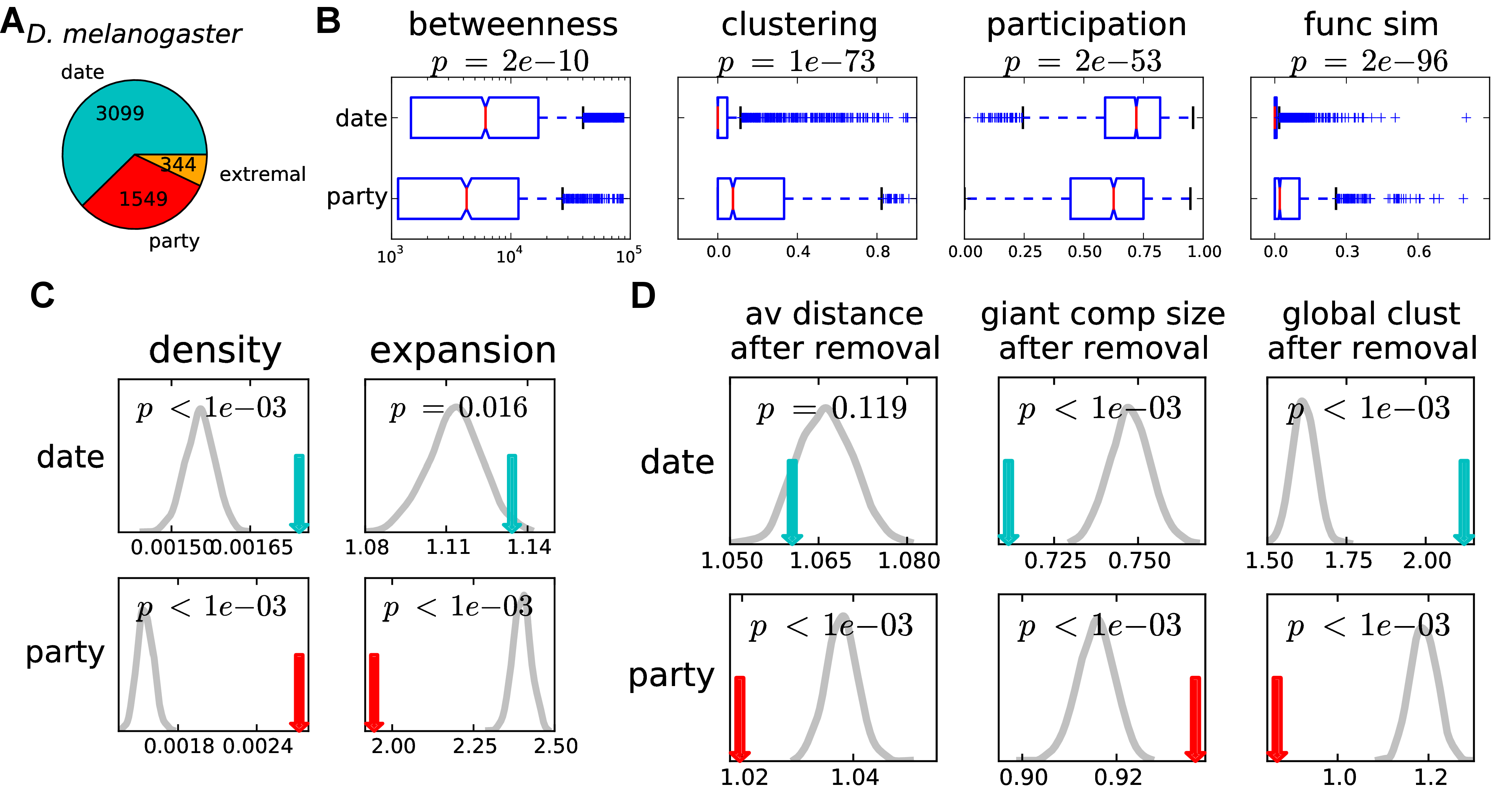

Supplement: Figure S10 — Date and party hub classification analysis in fly network of all physical interactions ( Fly ), with all genes of degree as hubs. (A) Number of hubs in each class. Party hubs in this network have avPCC; this threshold corresponds to the top third of avPCC values for all hubs categorized as either party or date. (B) Betweenness, clustering coefficient, participation coefficient and functional similarity for date and party hubs. (C) Density and expansion of date and party hubs. (D) Effect of hub removal for party and date when considering the average path distance, the size of the largest connected component, and the global clustering coefficient. See caption of Fig. 1 in the main text for details. (TIF) [file pcbi.1003243.s010.tif]

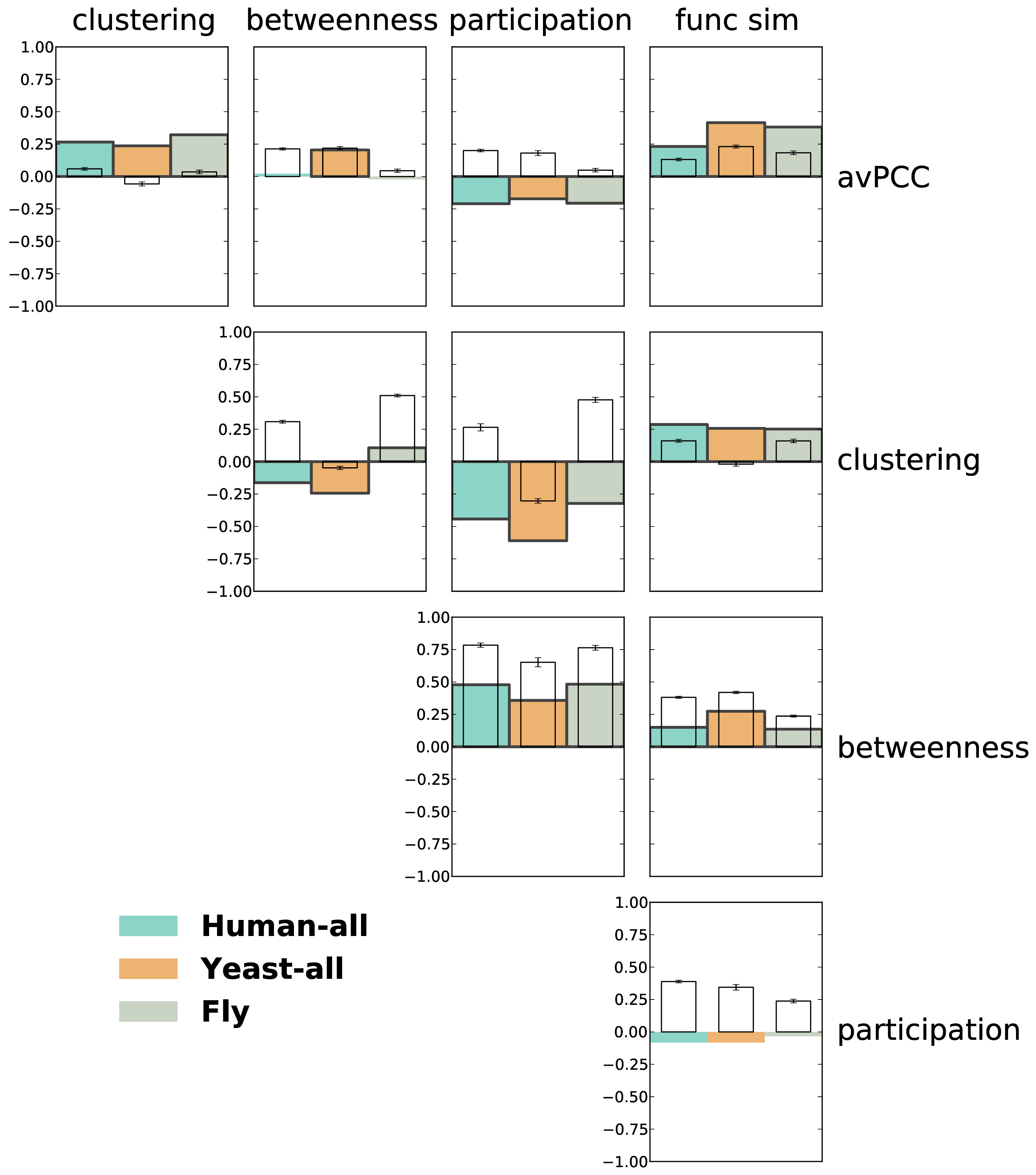

Supplement: Figure S11 — Spearman correlation of hub characteristics in interaction networks, with all genes of degree as hubs. Every bar represents a Spearman correlation between two characteristics of hubs in one of the networks. Bars of significant correlations (absolute value , p-value) have black edges. Smaller uncolored bars show average correlation (with error bars depicting the standard deviations) in 20 random networks on the same genes with the same number of interactions for each. (TIF) [file pcbi.1003243.s011.tif]

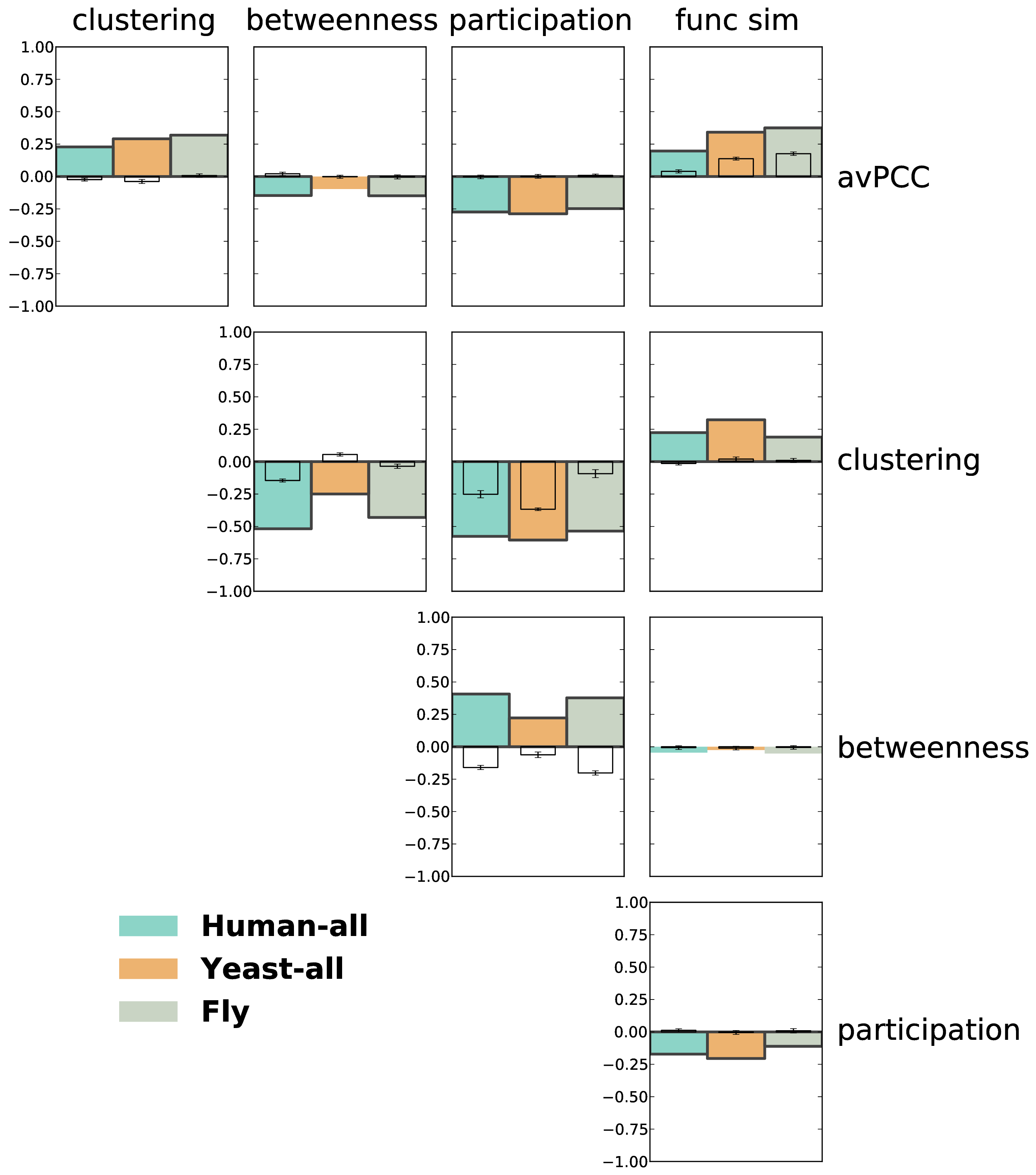

Supplement: Figure S12 — Spearman correlation of hub characteristics in interaction networks, with all genes of degree as hubs and with correction for degree. Every bar represents a partial Spearman correlation corrected for degree between two characteristics of hubs in one of the networks. Bars of significant correlations (absolute value , p-value) have black edges. Smaller uncolored bars show average correlation (with error bars depicting the standard deviations) in 20 random networks on the same genes with the same number of interactions for each. (TIF) [file pcbi.1003243.s012.tif]

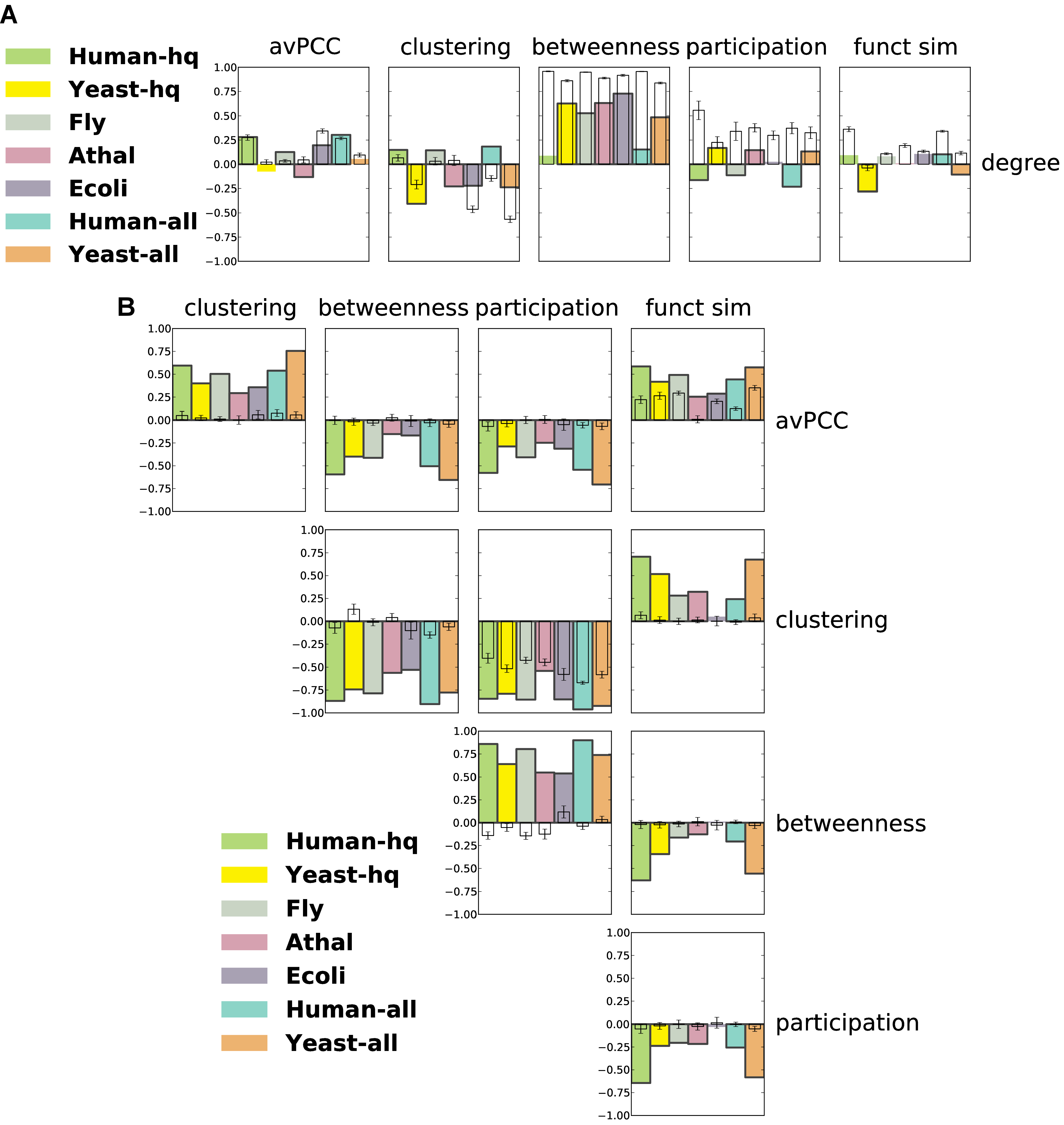

Supplement: Figure S13 — Correlation with degree is not a confounding factor in the correlation analysis of hub characteristics. (A) Every bar represents a Spearman correlation between a hub characteristic and degree (the number of interactions) for hubs in one of the networks. (B) Every bar represents a partial Spearman correlation corrected for degree between two characteristics of hubs in one of the networks. Bars of significant correlations (absolute value , p-value) have black edges. Smaller uncolored bars show average correlation (with error bars for standard deviations) in 20 random networks on the same genes with the same number of interactions for each. (TIF) [file pcbi.1003243.s013.tif]

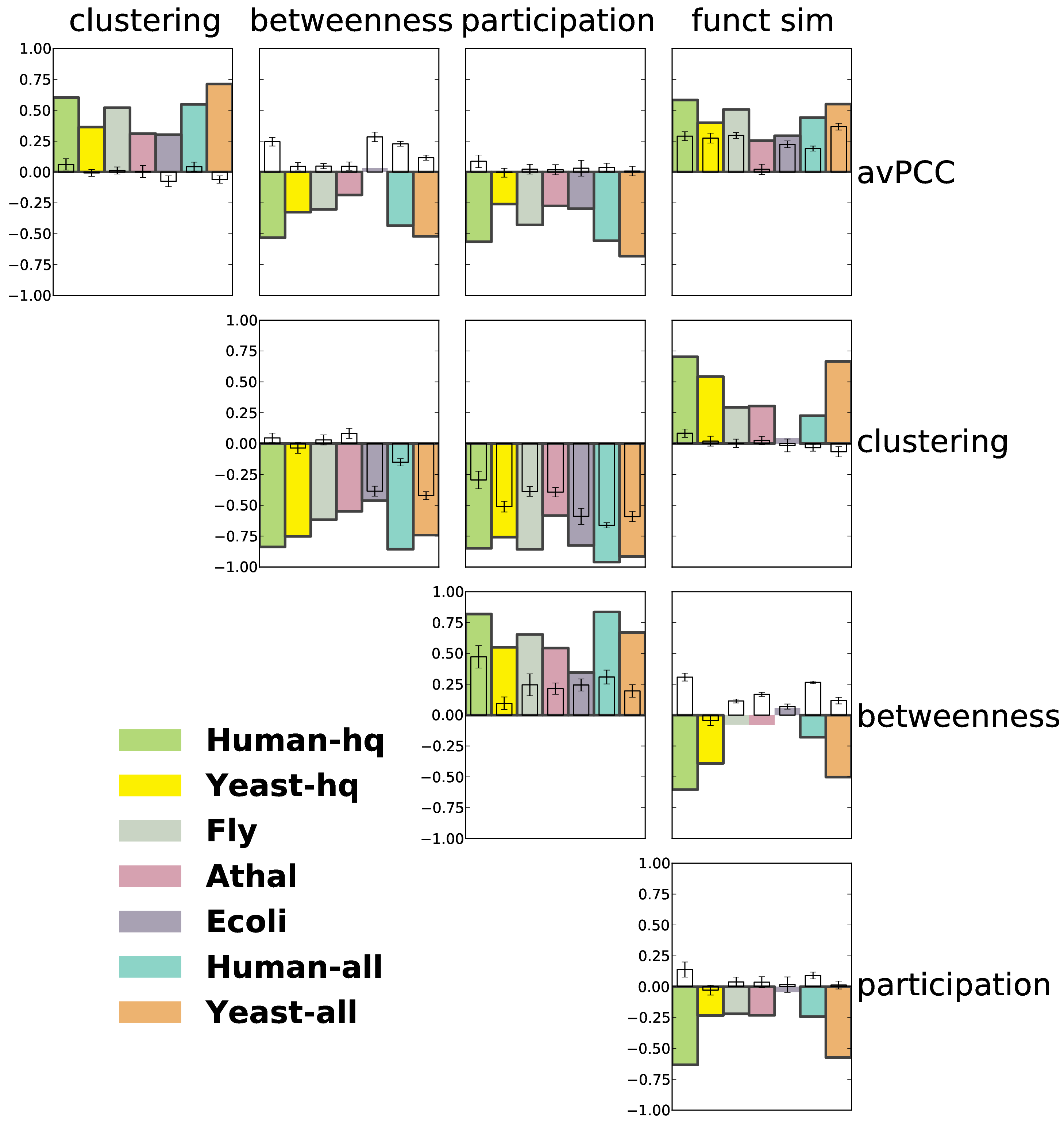

Supplement: Figure S14 — Hubs with extremal properties do not bias the correlation analysis of hub characteristics. Every bar represents a Spearman correlation between two characteristics of non-extremal hubs in one of the networks. Bars of significant correlations (absolute value , p-value) have black edges. Smaller uncolored bars show average correlation (with error bars for standard deviations) in 20 random networks on the same genes with the same number of interactions for each. (TIF) [file pcbi.1003243.s014.tif]

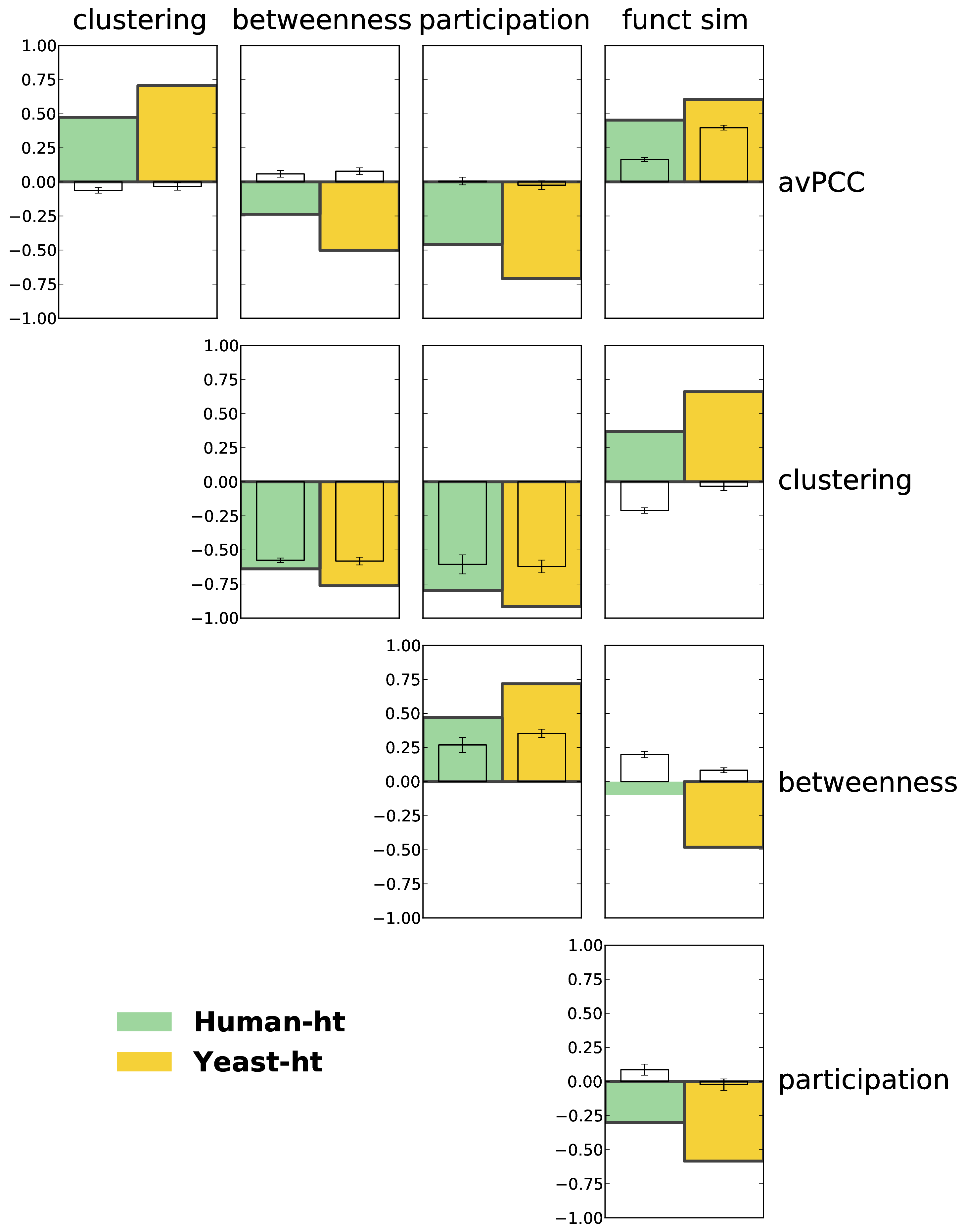

Supplement: Figure S15 — Spearman correlation of hub characteristics in high-throughput interaction networks for human and yeast. Every bar represents a Spearman correlation between two characteristics of hubs in one of the networks. Bars of significant correlations (absolute value , p-value) have black edges. Smaller uncolored bars show average correlation (with error bars for standard deviations) in 20 random networks on the same genes with the same number of interactions for each. (TIF) [file pcbi.1003243.s015.tif]

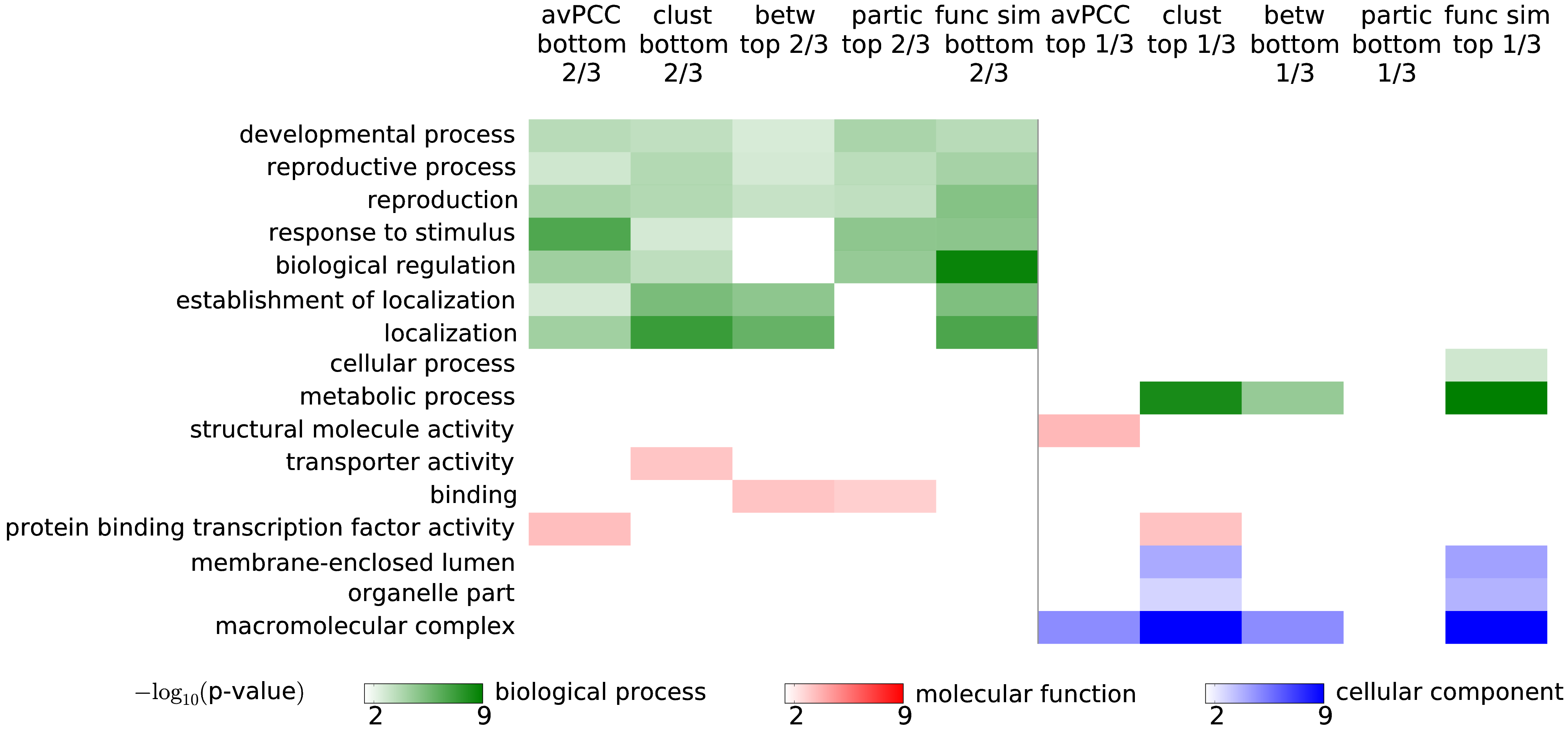

Supplement: Figure S16 — GO annotation enrichment analysis of hubs in Yeast-hq. GO annotation enrichment analysis of hubs divided in a 2-to-1 proportion by avPCC, clustering, betweenness, participation and functional similarity scores in Yeast-hq. See Fig. 3 in the main text for details. (TIF) [file pcbi.1003243.s016.tif]

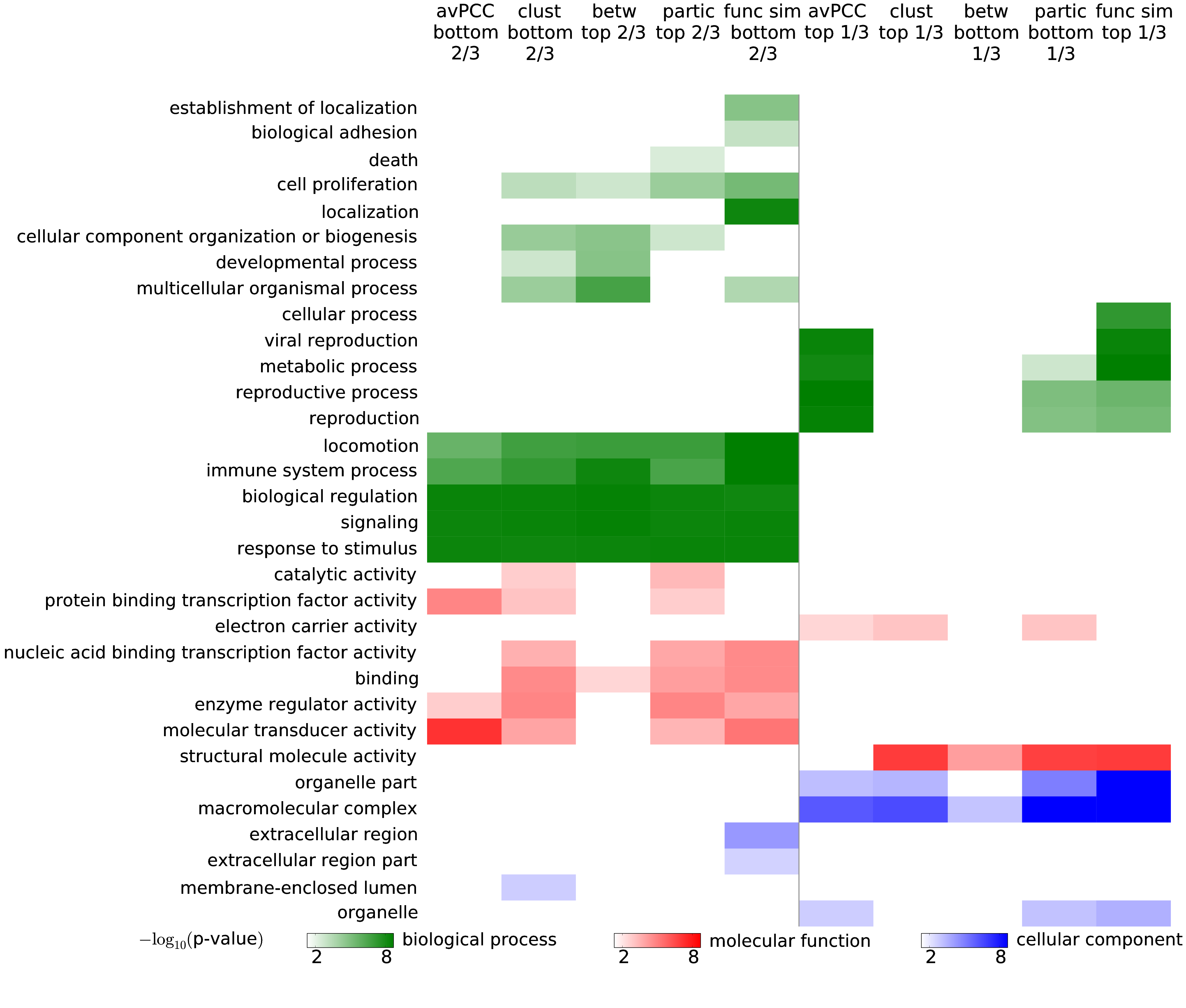

Supplement: Figure S17 — GO annotation enrichment analysis of hubs in Human-all. GO annotation enrichment analysis of hubs divided in a 2-to-1 proportion by avPCC, clustering, betweenness, participation and functional similarity scores in Human-all. See Fig. 3 in the main text for details. (TIF) [file pcbi.1003243.s017.tif]

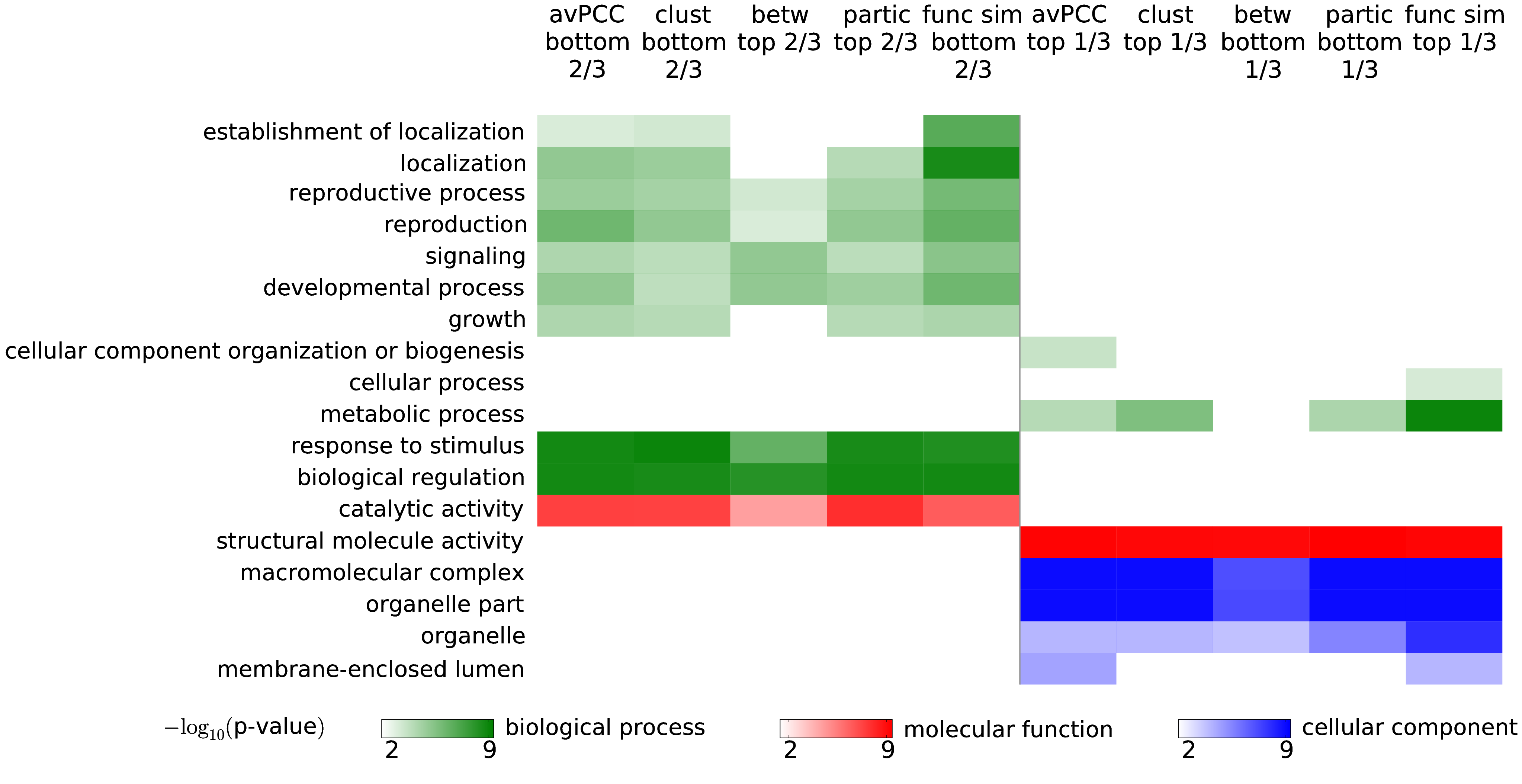

Supplement: Figure S18 — GO annotation enrichment analysis of hubs in Yeast-all. GO annotation enrichment analysis of hubs divided in a 2-to-1 proportion by avPCC, clustering, betweenness, participation and functional similarity scores in Yeast-all. See Fig. 3 in the main text for details. (TIF) [file pcbi.1003243.s018.tif]

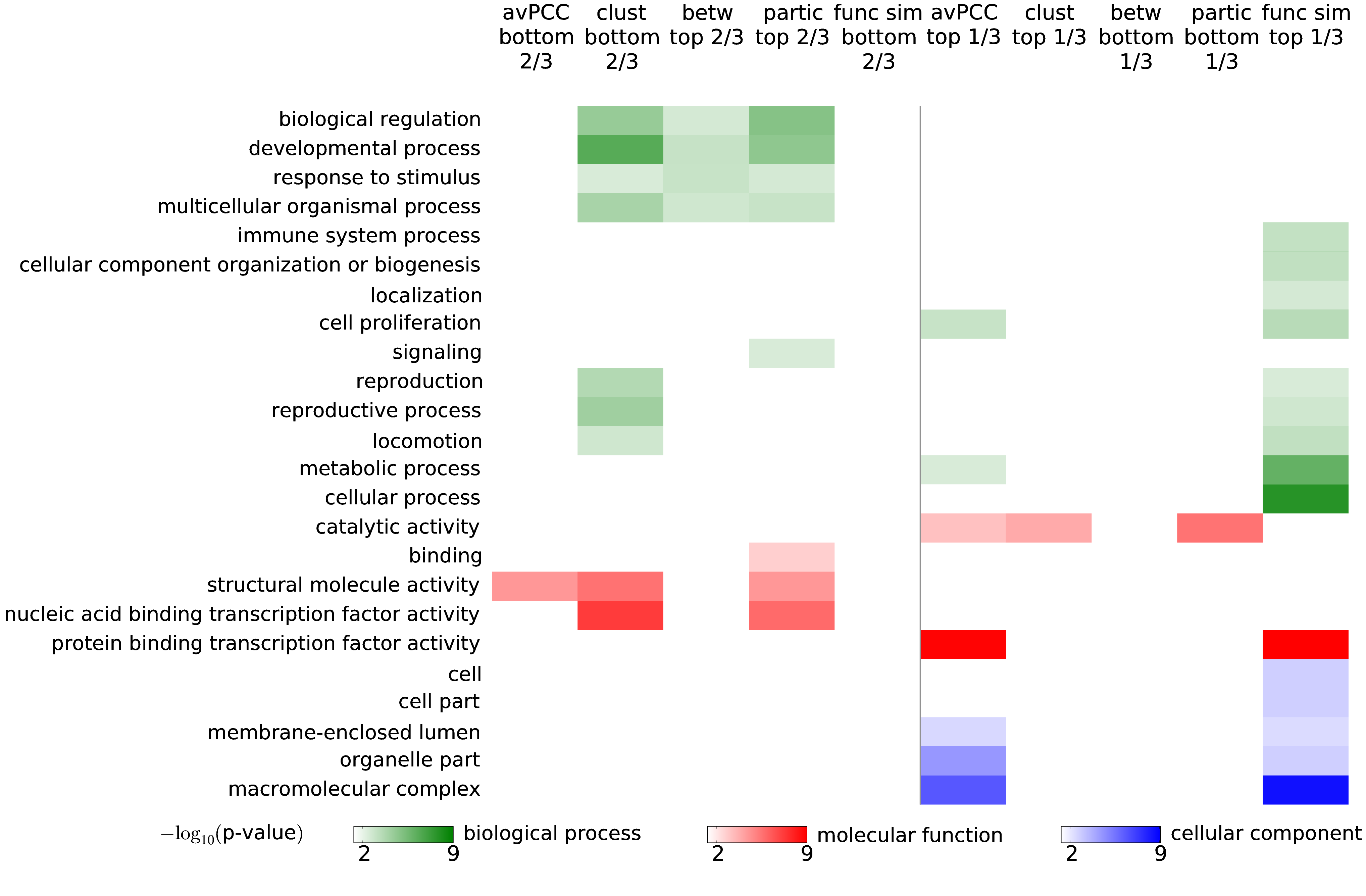

Supplement: Figure S19 — GO annotation enrichment analysis of hubs in Fly. GO annotation enrichment analysis of hubs divided in a 2-to-1 proportion by avPCC, clustering, betweenness, participation and functional similarity scores in Fly. See Fig. 3 in the main text for details. (TIF) [file pcbi.1003243.s019.tif]

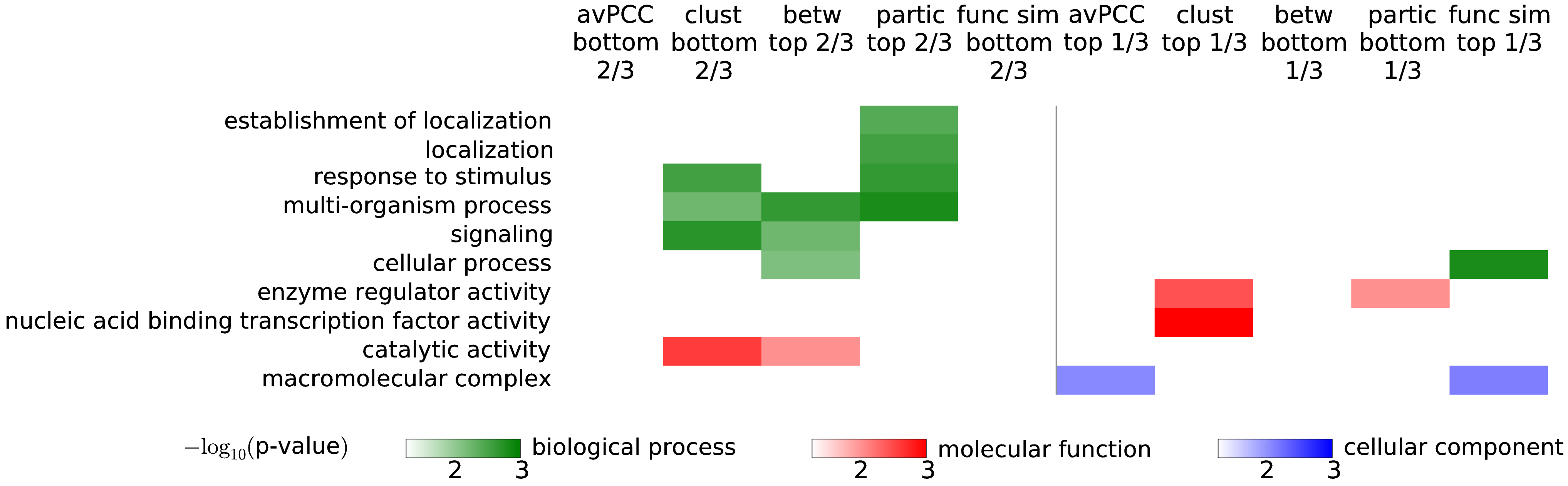

Supplement: Figure S20 — GO annotation enrichment analysis of hubs in Athal. GO annotation enrichment analysis of hubs divided in a 2-to-1 proportion by avPCC, clustering, betweenness, participation and functional similarity scores in Athal. See Fig. 3 in the main text for details. (TIF) [file pcbi.1003243.s020.tif]

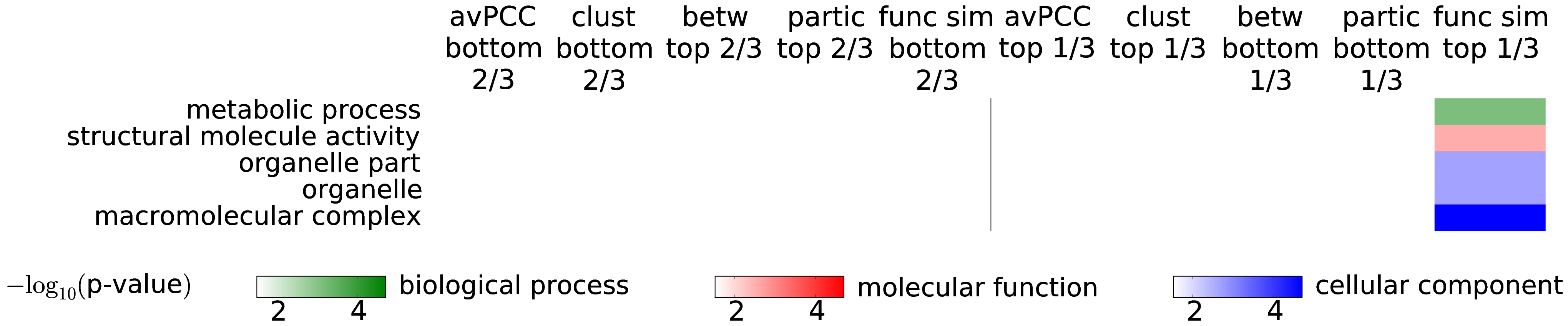

Supplement: Figure S21 — GO annotation enrichment analysis of hubs in Ecoli. GO annotation enrichment analysis of hubs divided in a 2-to-1 proportion by avPCC, clustering, betweenness, participation and functional similarity scores in Ecoli. See Fig. 3 in the main text for details. (TIF) [file pcbi.1003243.s021.tif]

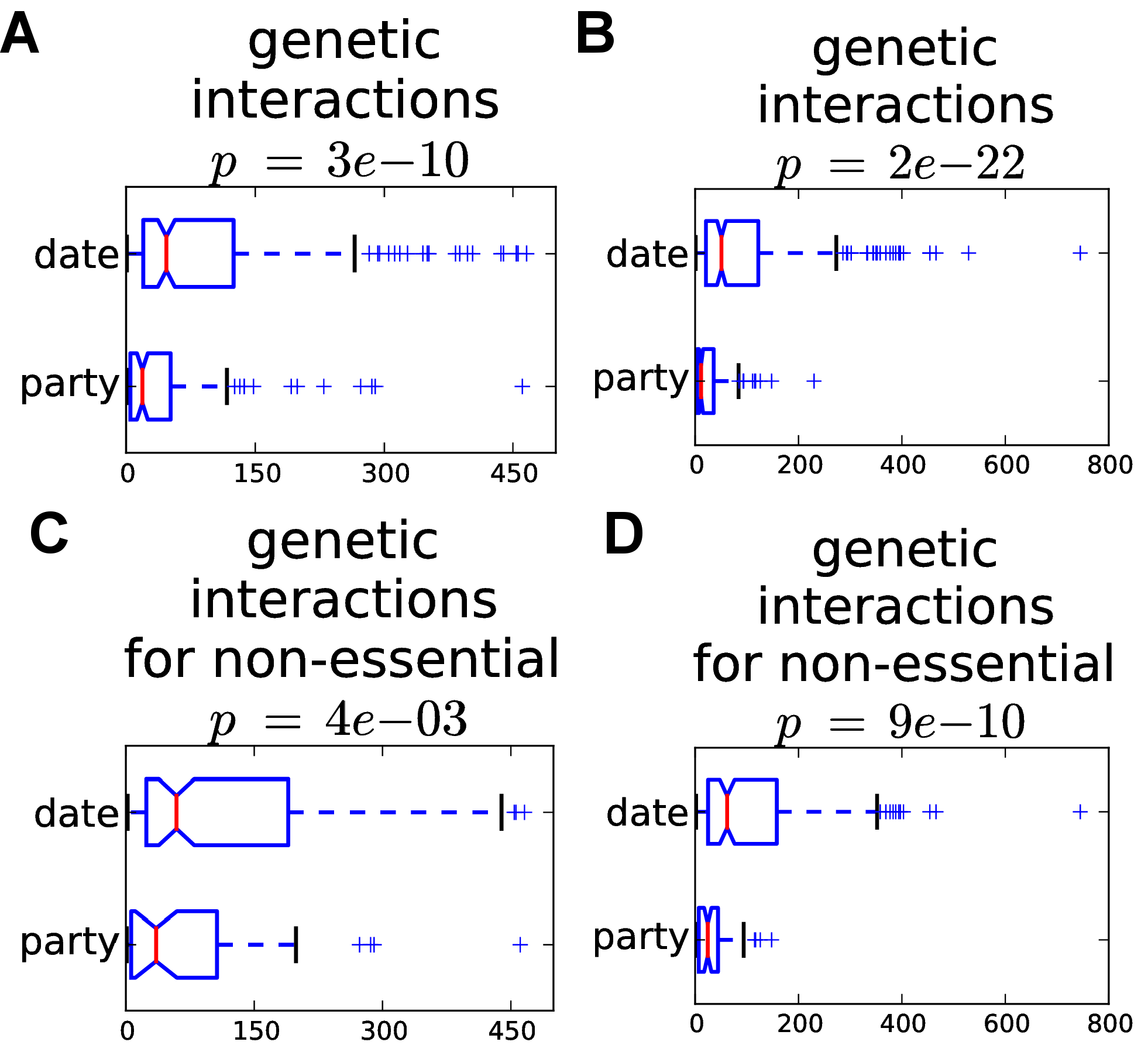

Supplement: Figure S22 — Genetic interactions for date and party hubs in yeast. Date hubs participate in significantly larger number of genetic interactions than party hubs, when date and party hubs are defined from yeast networks (A) Yeast-hq (B) Yeast-all (Mann–Whitney U). Even when all essential genes are removed from consideration, the same trend is observed for both networks (C) Yeast-hq and (D) Yeast-all. (TIF) [file pcbi.1003243.s022.tif]

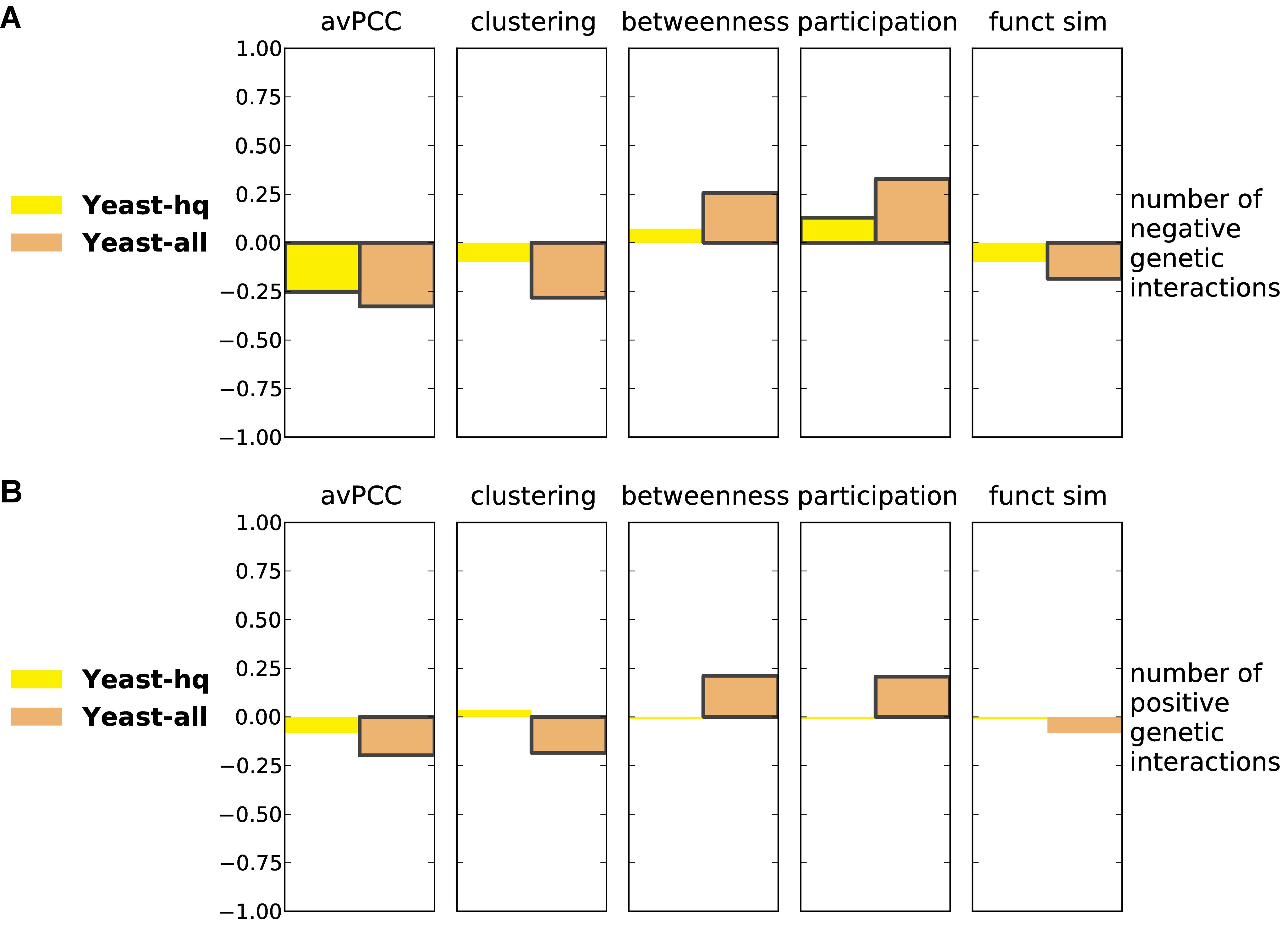

Supplement: Figure S23 — Spearman correlation of hub characteristics with the number of negative and positive genetic interactions. (A) Every bar represents a Spearman correlation between a hub characteristic and the number of negative genetic interactions for hubs in one of the physical interaction networks for yeast. (B) Every bar represents a Spearman correlation between a hub characteristic and the number of positive genetic interactions for hubs in one of the physical interaction networks for yeast. Bars of significant correlations (absolute value , p-value) have black edges. (TIF) [file pcbi.1003243.s023.tif]

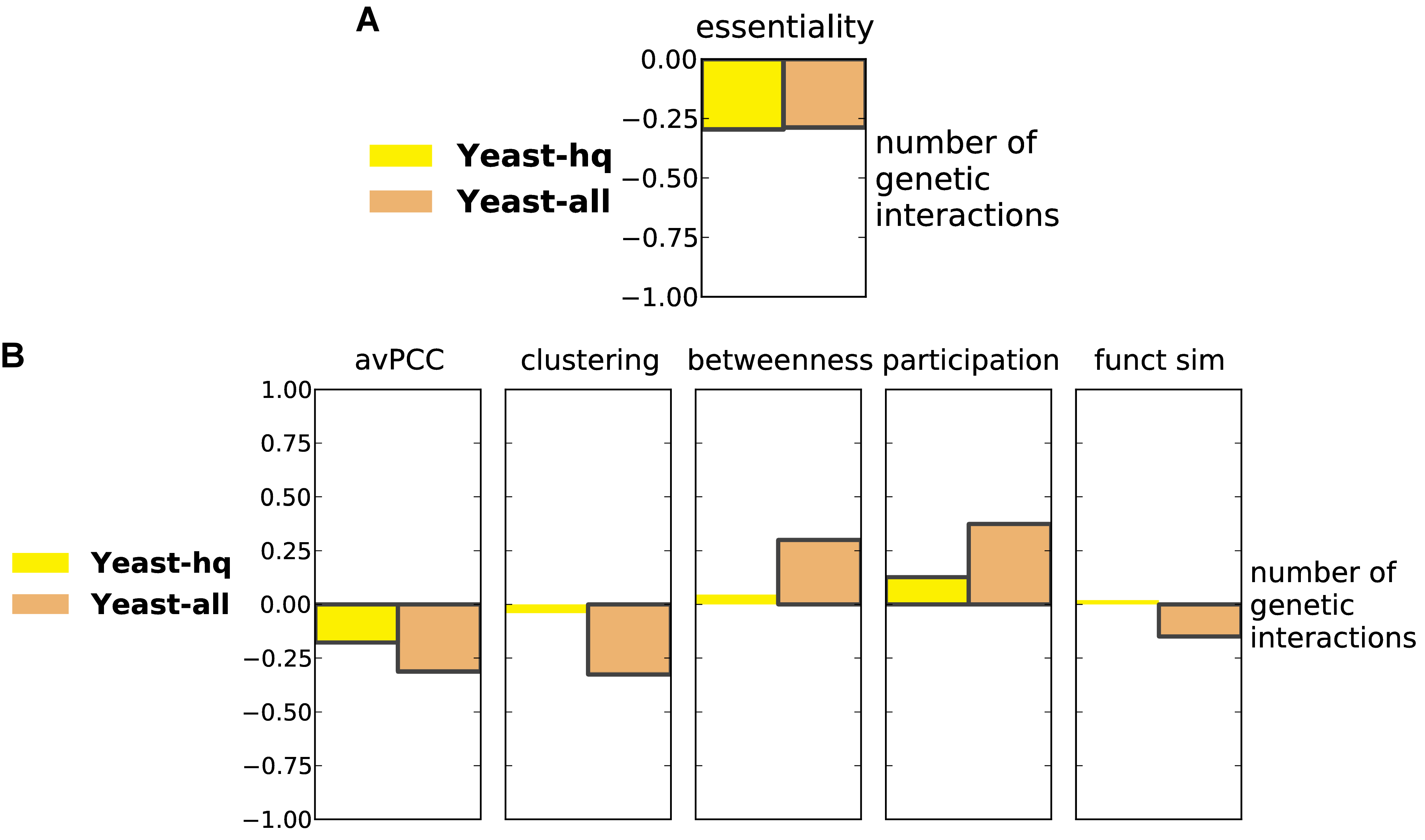

Supplement: Figure S24 — Essentiality is not a confounding factor in the correlation analysis of genetic degree with hub characteristics in yeast physical interaction networks. (A) Every bar represents a Spearman correlation between essentiality (1 if essential, 0 otherwise) and the number of genetic interactions for hubs in one of the physical interaction networks for yeast. (B) Every bar represents a partial Spearman correlation between a hub characteristic and the number of genetic interactions corrected for essentiality for hubs in one of the physical interaction networks for yeast. Bars of significant correlations (absolute value , p-value) have black edges. (TIF) [file pcbi.1003243.s024.tif]

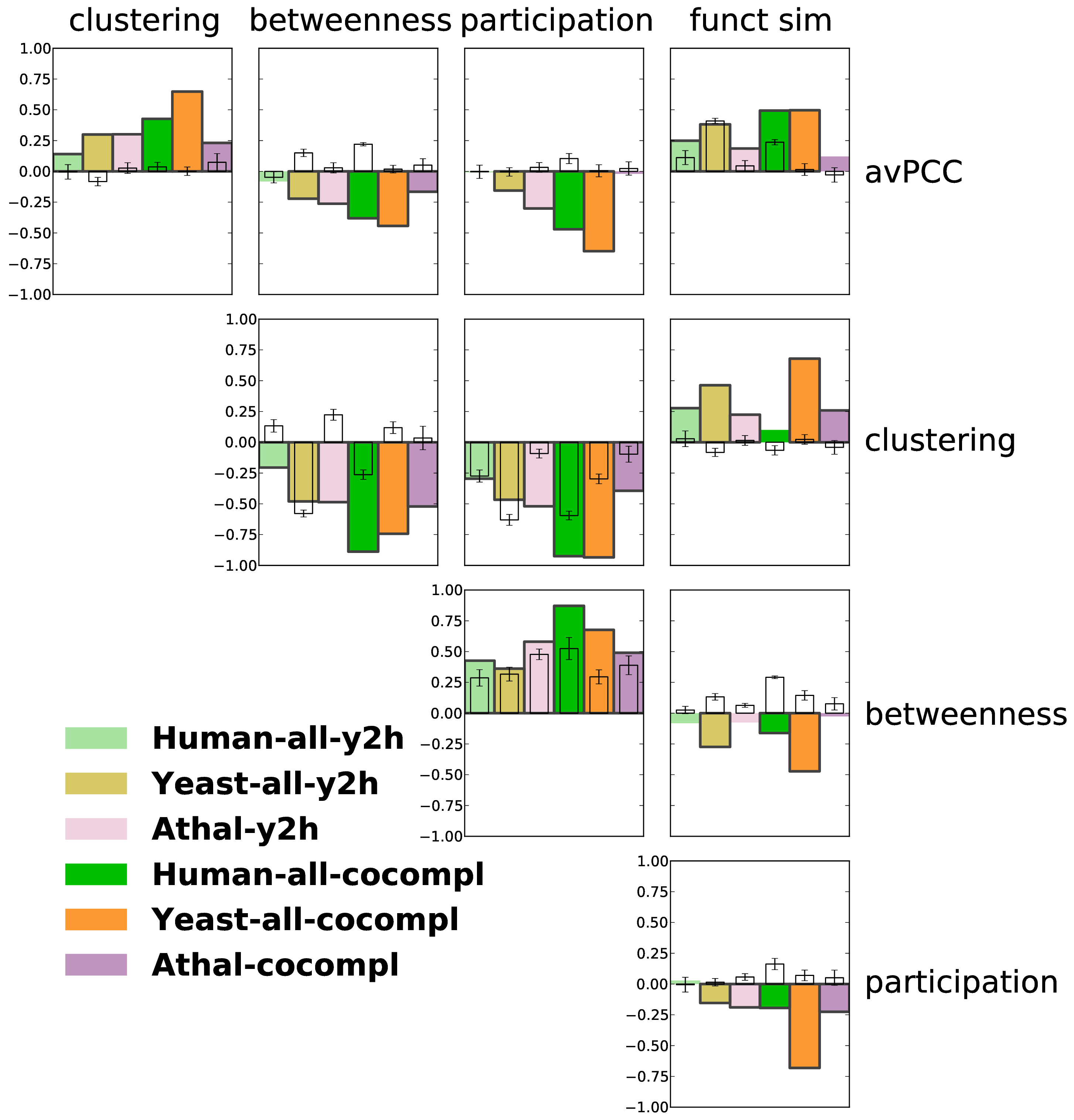

Supplement: Figure S25 — Spearman correlation of hub characteristics in yeast two-hybrid and co-complex interaction networks. Every bar represents a Spearman correlation between two characteristics of hubs in one of the networks. Bars of significant correlations (absolute value , p-value) have black edges. Smaller uncolored bars show average correlation (with error bars for standard deviations) in 20 random networks on the same genes with the same number of interactions for each. (TIF) [file pcbi.1003243.s025.tif]

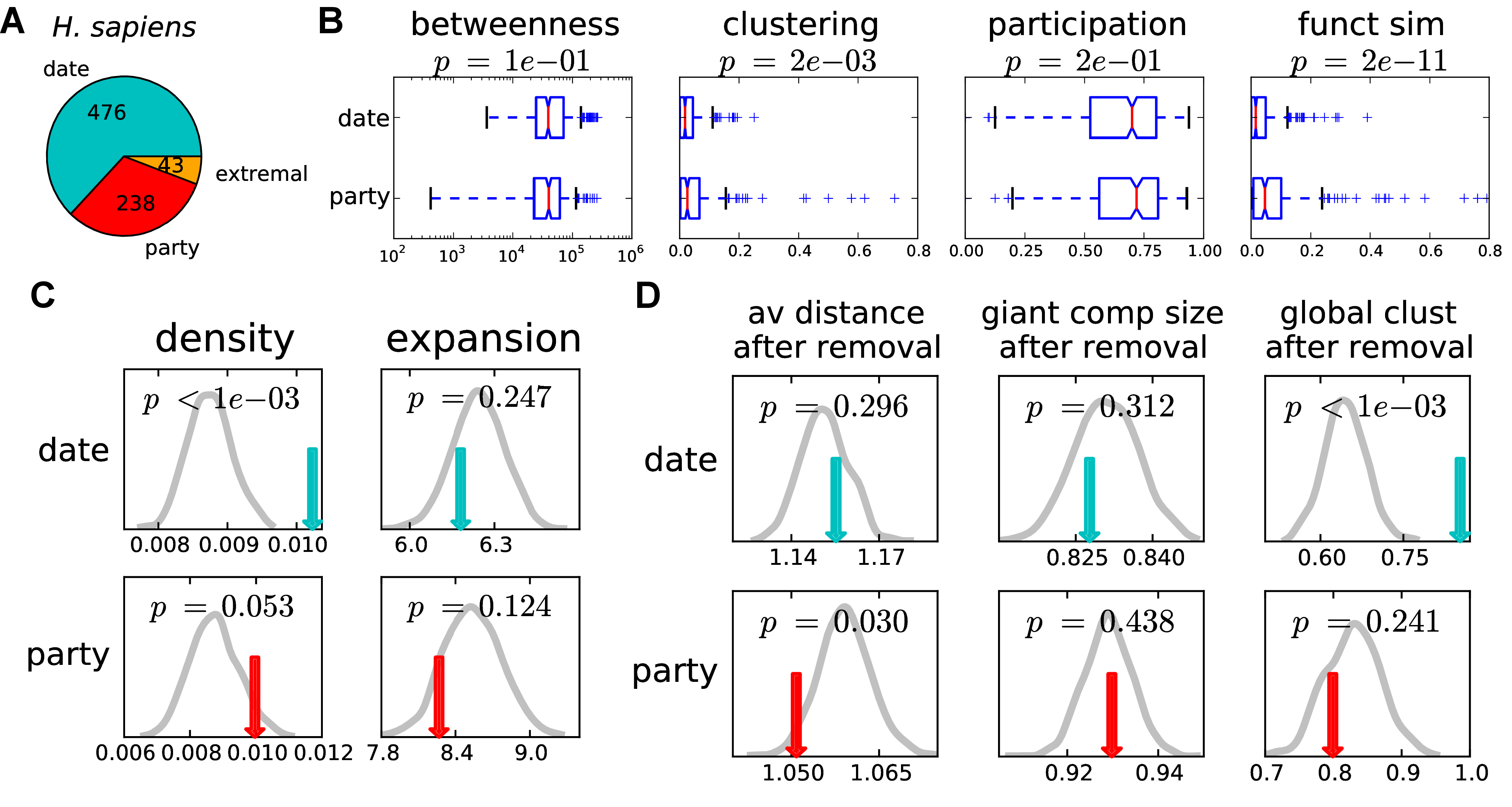

Supplement: Figure S26 — Date and party hub classification analysis in human network of all known interactions from yeast two-hybrid experiments (Human-all-y2h). (A) Number of hubs in each class. Party hubs in this network have avPCC; this threshold corresponds to the top third of avPCC values for all hubs categorized as either party or date. (B) Betweenness, clustering coefficient, participation coefficient and functional similarity for date and party hubs. (C) Density and expansion of date and party hubs. (D) Effect of hub removal for party and date when considering the average path distance, the size of the largest connected component, and the global clustering coefficient. See caption of Fig. 1 in the main text for details. (TIF) [file pcbi.1003243.s026.tif]

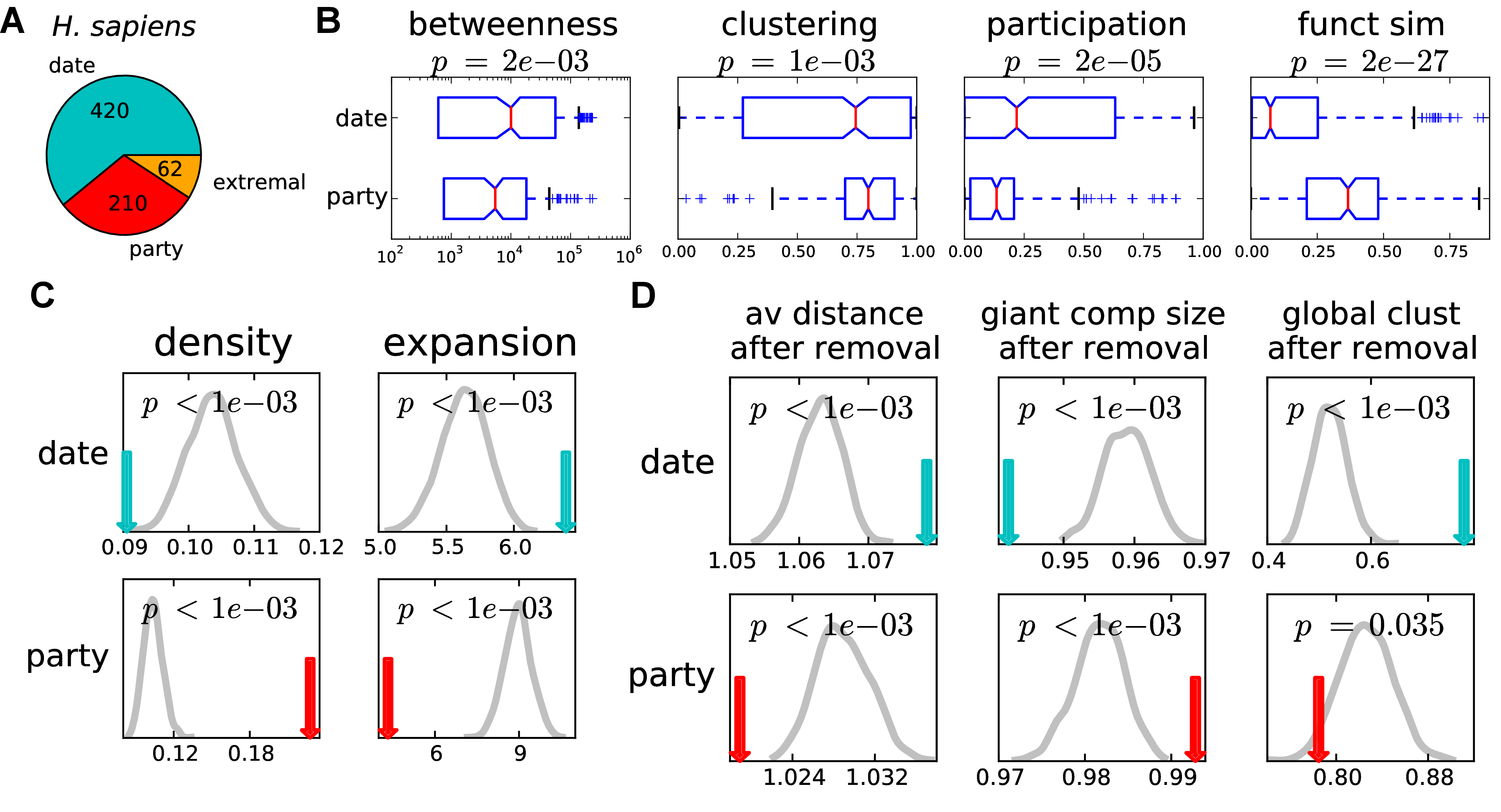

Supplement: Figure S27 — Date and party hub classification analysis in human network of all known interactions derived from complexes (Human-all-cocompl). (A) Number of hubs in each class. Party hubs in this network have avPCC; this threshold corresponds to the top third of avPCC values for all hubs categorized as either party or date. (B) Betweenness, clustering coefficient, participation coefficient and functional similarity for date and party hubs. (C) Density and expansion of date and party hubs. (D) Effect of hub removal for party and date when considering the average path distance, the size of the largest connected component, and the global clustering coefficient. See caption of Fig. 1 in the main text for details. (TIF) [file pcbi.1003243.s027.tif]

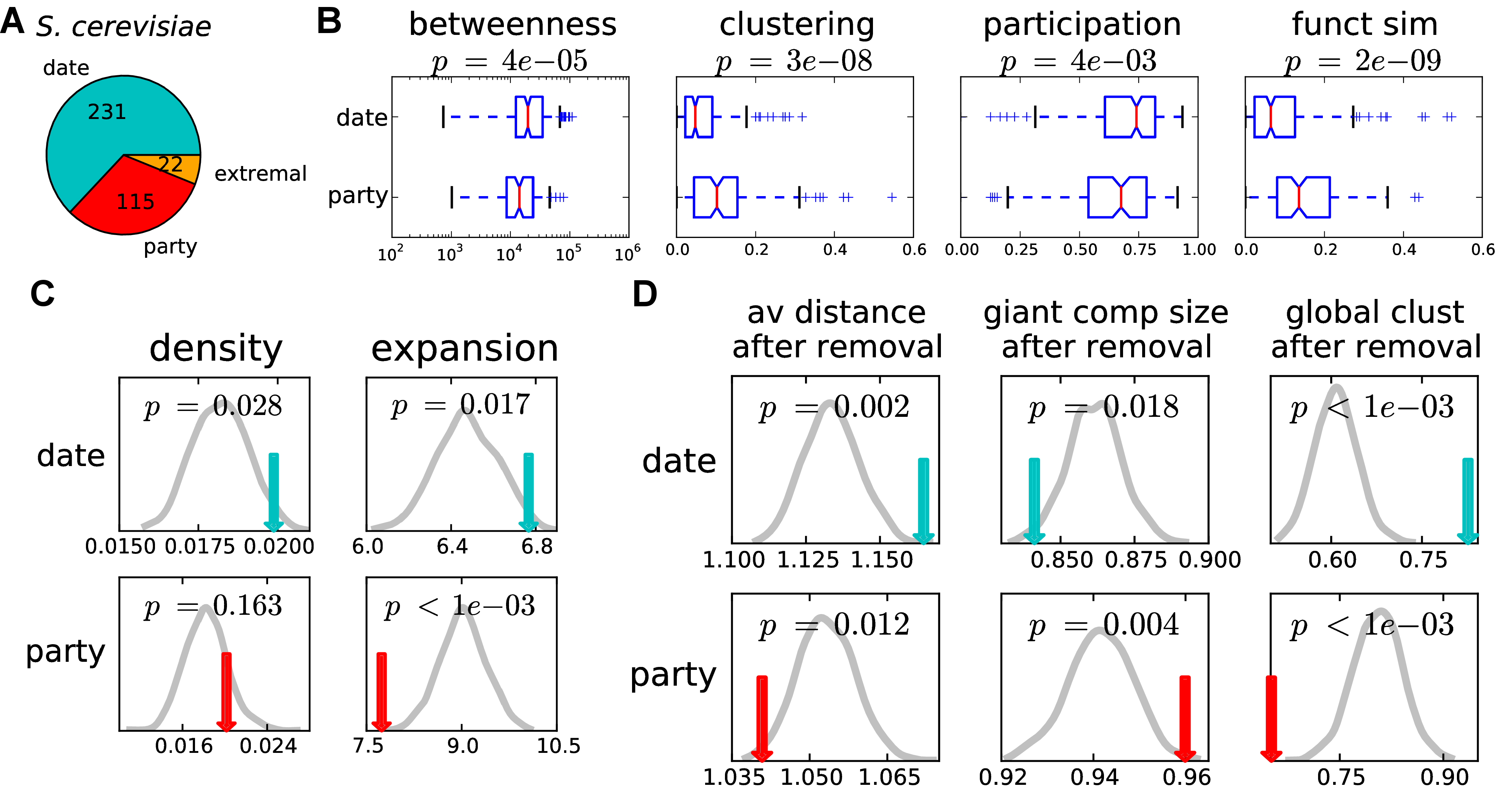

Supplement: Figure S28 — Date and party hub classification analysis in yeast network of all known interactions from yeast two-hybrid experiments (Yeast-all-y2h). (A) Number of hubs in each class. Party hubs in this network have avPCC; this threshold corresponds to the top third of avPCC values for all hubs categorized as either party or date. (B) Betweenness, clustering coefficient, participation coefficient and functional similarity for date and party hubs. (C) Density and expansion of date and party hubs. (D) Effect of hub removal for party and date when considering the average path distance, the size of the largest connected component, and the global clustering coefficient. See caption of Fig. 1 in the main text for details. (TIF) [file pcbi.1003243.s028.tif]

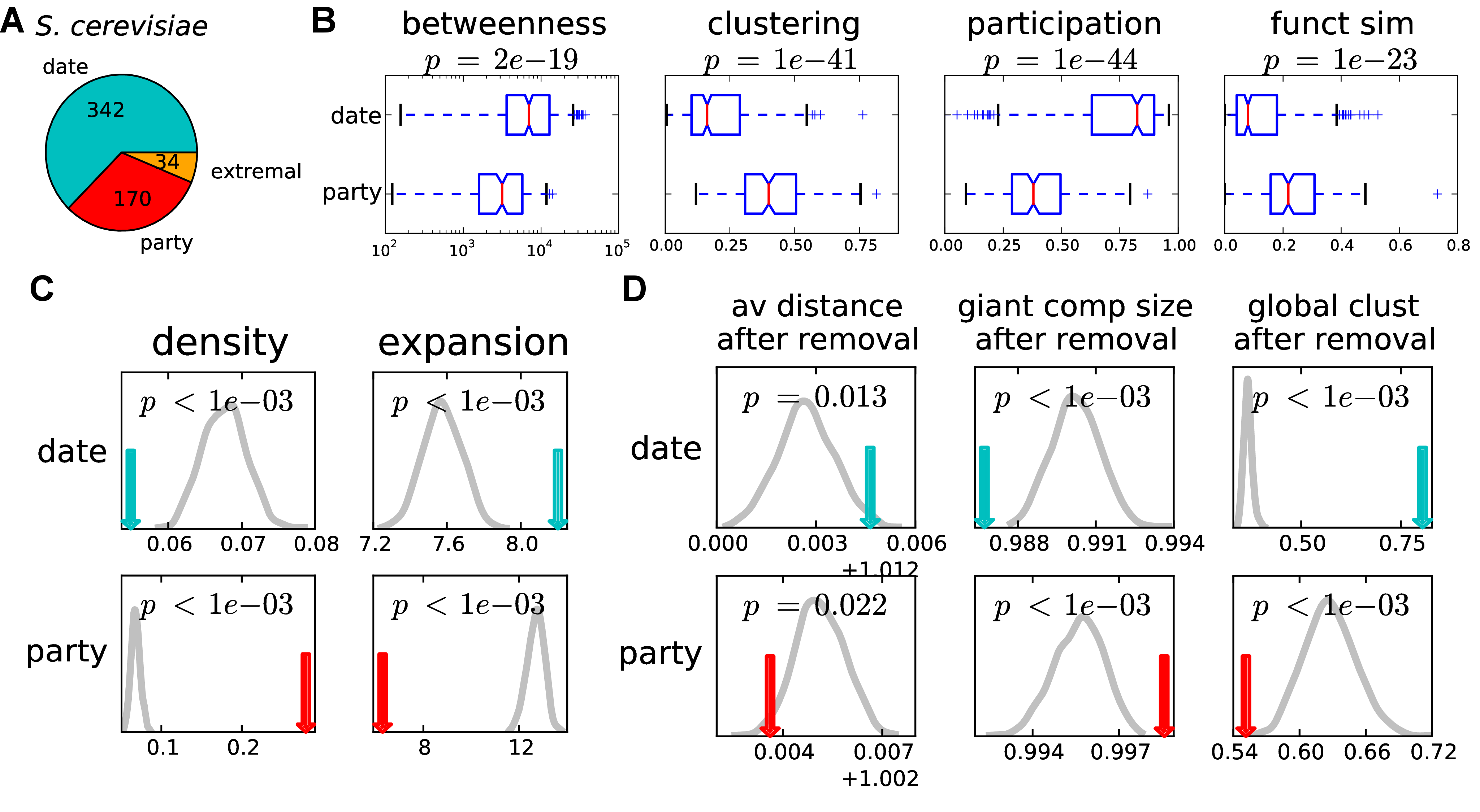

Supplement: Figure S29 — Date and party hub classification analysis in the yeast network of all known interactions derived from complexes (Yeast-all-cocompl). (A) Number of hubs in each class. Party hubs in this network have avPCC; this threshold corresponds to the top third of avPCC values for all hubs categorized as either party or date. (B) Betweenness, clustering coefficient, participation coefficient and functional similarity for date and party hubs. (C) Density and expansion of date and party hubs. (D) Effect of hub removal for party and date when considering the average path distance, the size of the largest connected component, and the global clustering coefficient. See caption of Fig. 1 in the main text for details. (TIF) [file pcbi.1003243.s029.tif]

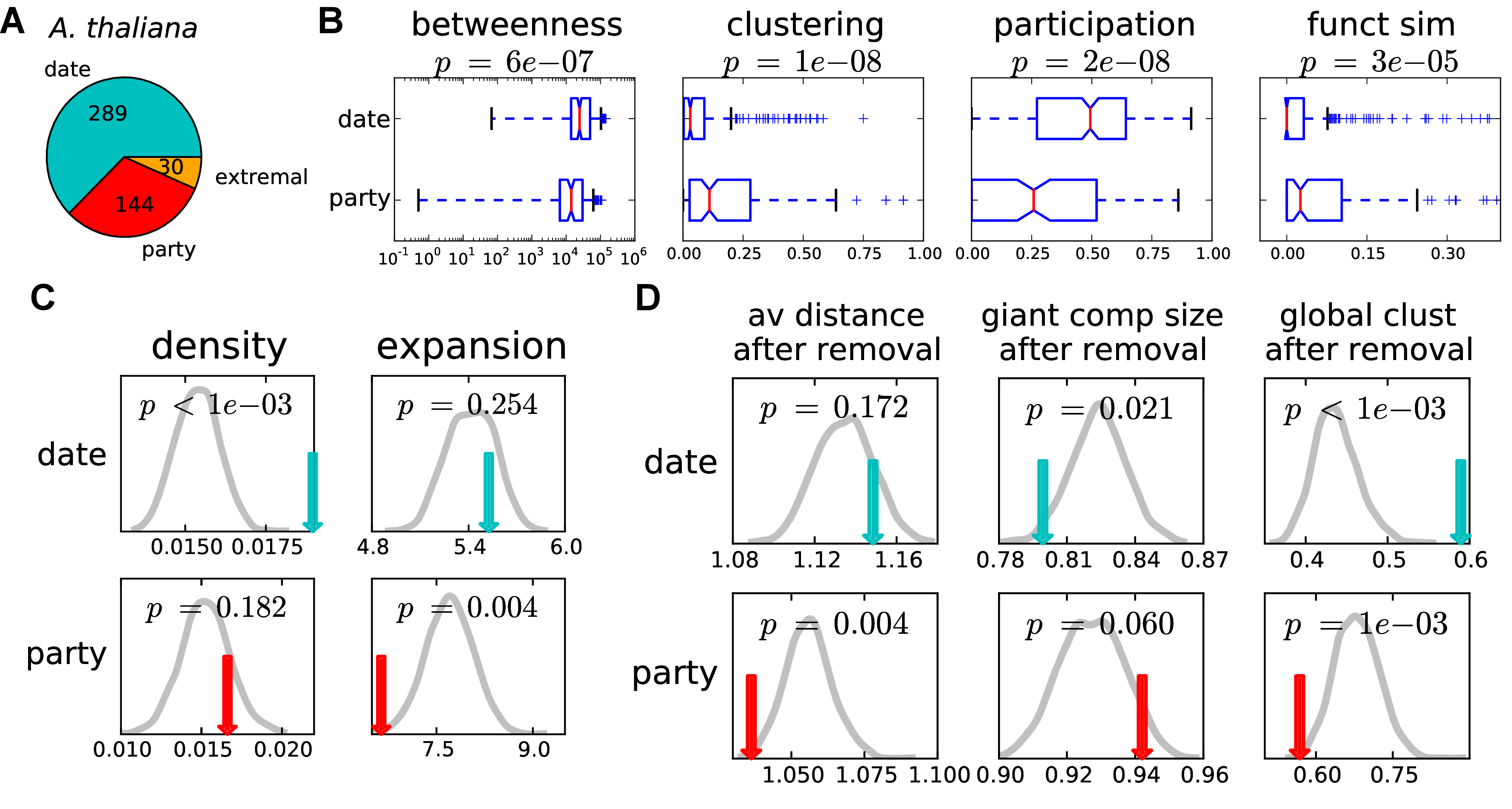

Supplement: Figure S30 — Date and party hub classification analysis in the Arabidopsis network of all known interactions from yeast two-hybrid experiments (Athal-y2h). (A) Number of hubs in each class. Party hubs in this network have avPCC; this threshold corresponds to the top third of avPCC values for all hubs categorized as either party or date. (B) Betweenness, clustering coefficient, participation coefficient and functional similarity for date and party hubs. (C) Density and expansion of date and party hubs. (D) Effect of hub removal for party and date when considering the average path distance, the size of the largest connected component, and the global clustering coefficient. See caption of Fig. 1 in the main text for details. (TIF) [file pcbi.1003243.s030.tif]

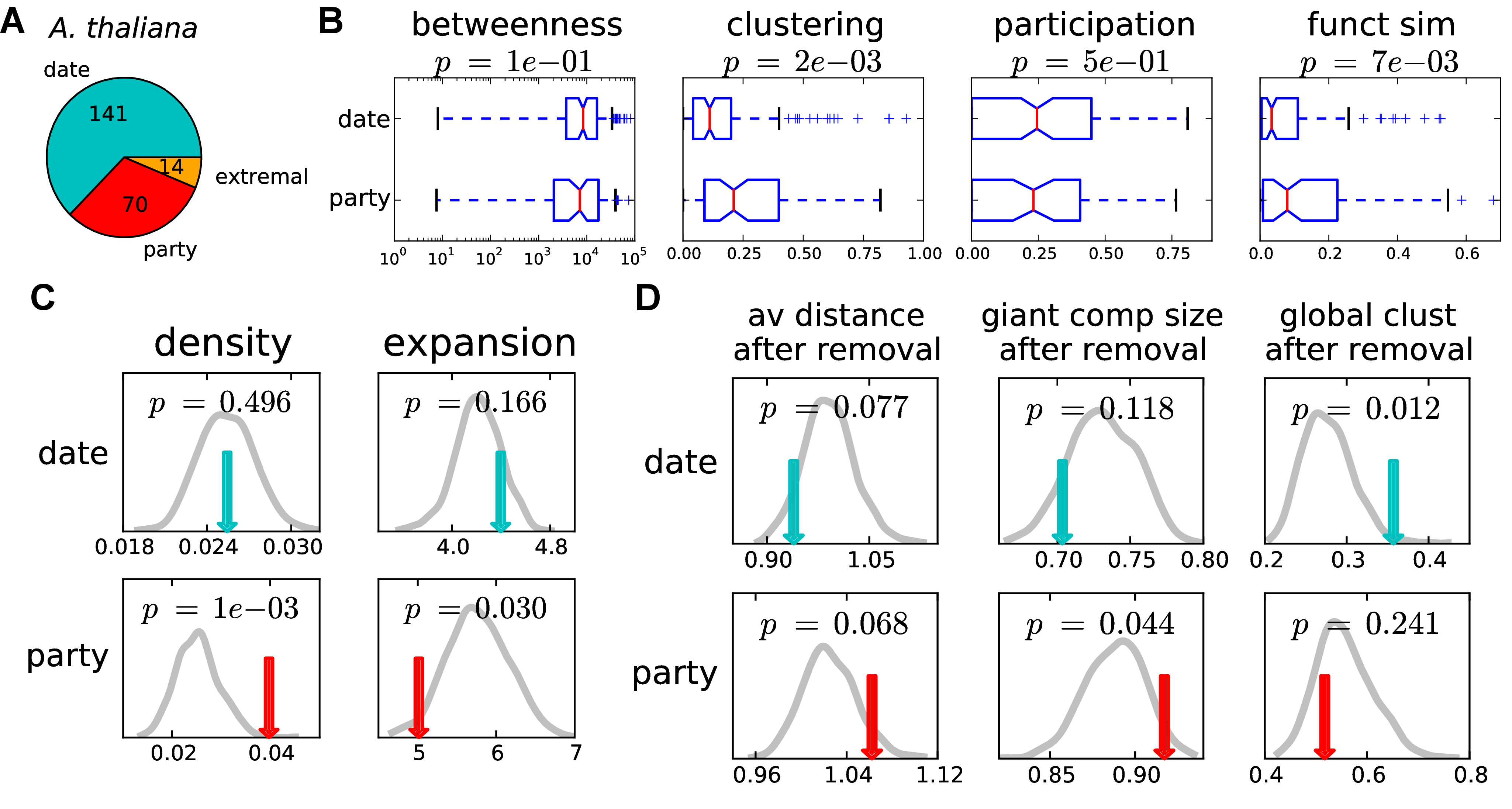

Supplement: Figure S31 — Date and party hub classification analysis in Arabidopsis network of all known interactions derived from complexes (Athal-cocompl). (A) Number of hubs in each class. Party hubs in this network have avPCC; this threshold corresponds to the top third of avPCC values for all hubs categorized as either party or date. (B) Betweenness, clustering coefficient, participation coefficient and functional similarity for date and party hubs. (C) Density and expansion of date and party hubs. (D) Effect of hub removal for party and date when considering the average path distance, the size of the largest connected component, and the global clustering coefficient. See caption of Fig. 1 in the main text for details. (TIF) [file pcbi.1003243.s031.tif]

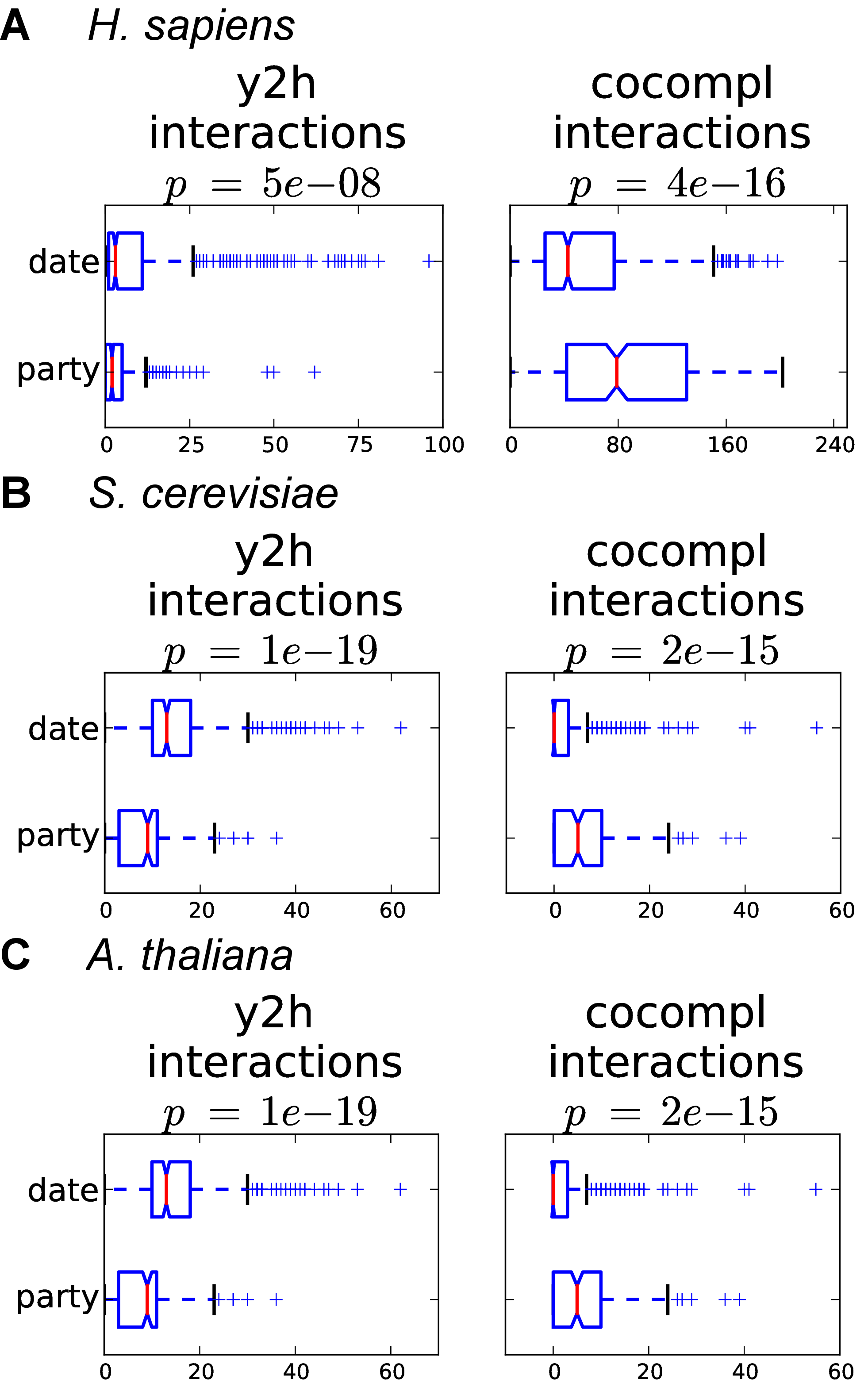

Supplement: Figure S32 — Yeast two-hybrid and co-complex interactions of date and party hubs. Date hubs have significantly many more binary (yeast two-hybrid, y2h) interactions, while party hubs participate in significantly larger number of interactions derived from complexes (co-complex, cocompl) in networks (A) Human-all (B) Yeast-all (C) Athal (Mann–Whitney U). (TIF) [file pcbi.1003243.s032.tif]

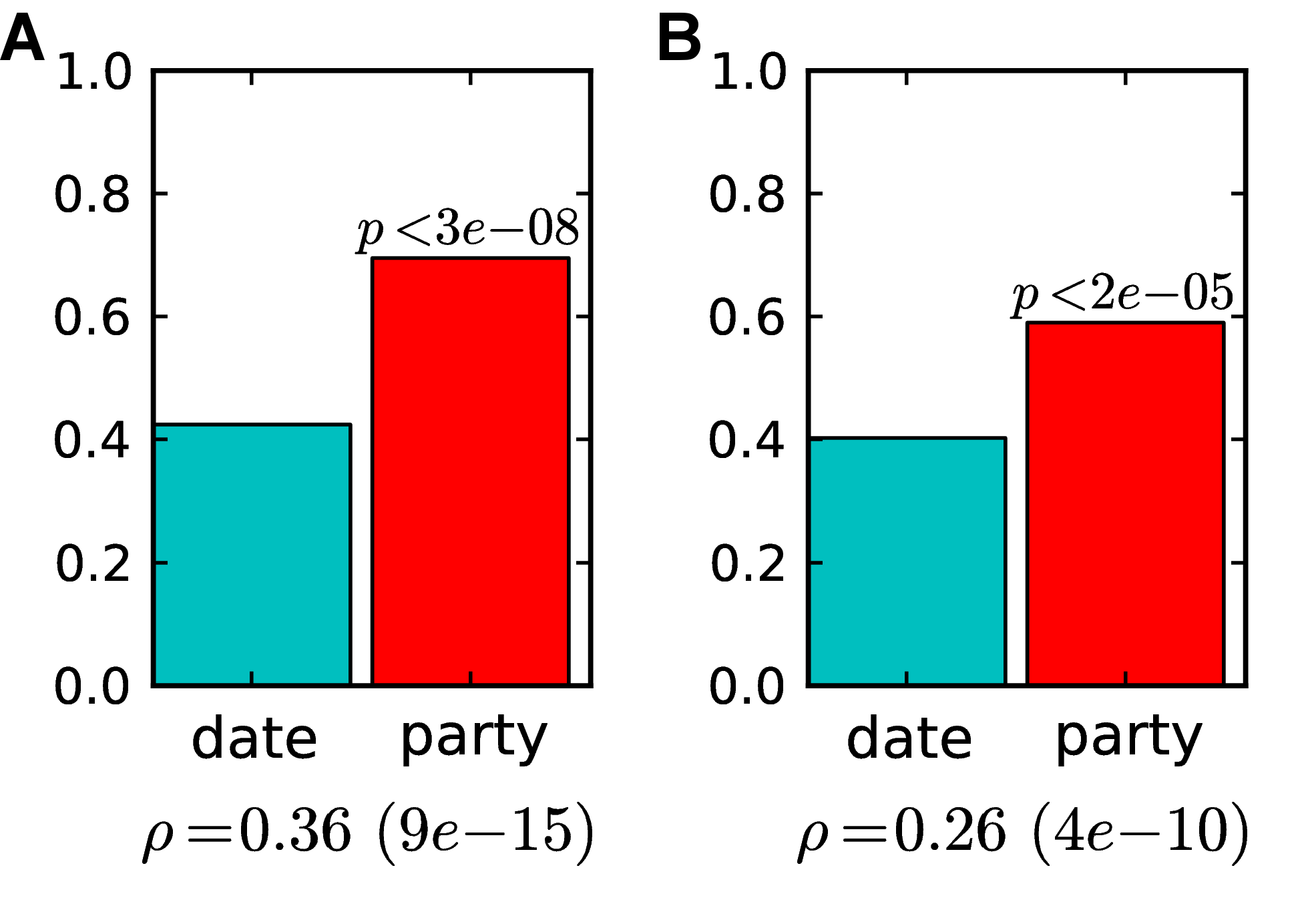

Supplement: Figure S33 — Party hubs are more likely to be essential than date hubs. Fraction of date and party hubs that are essential in (A) Yeast-hq (B) Yeast-all. Party hubs are significantly enriched with essential genes (hypergeometric test). Spearman correlation (with p-value) of essentiality indicator vector (1 if essential, 0 otherwise) and avPCC shown on bottom is significantly positive. (TIF) [file pcbi.1003243.s033.tif]

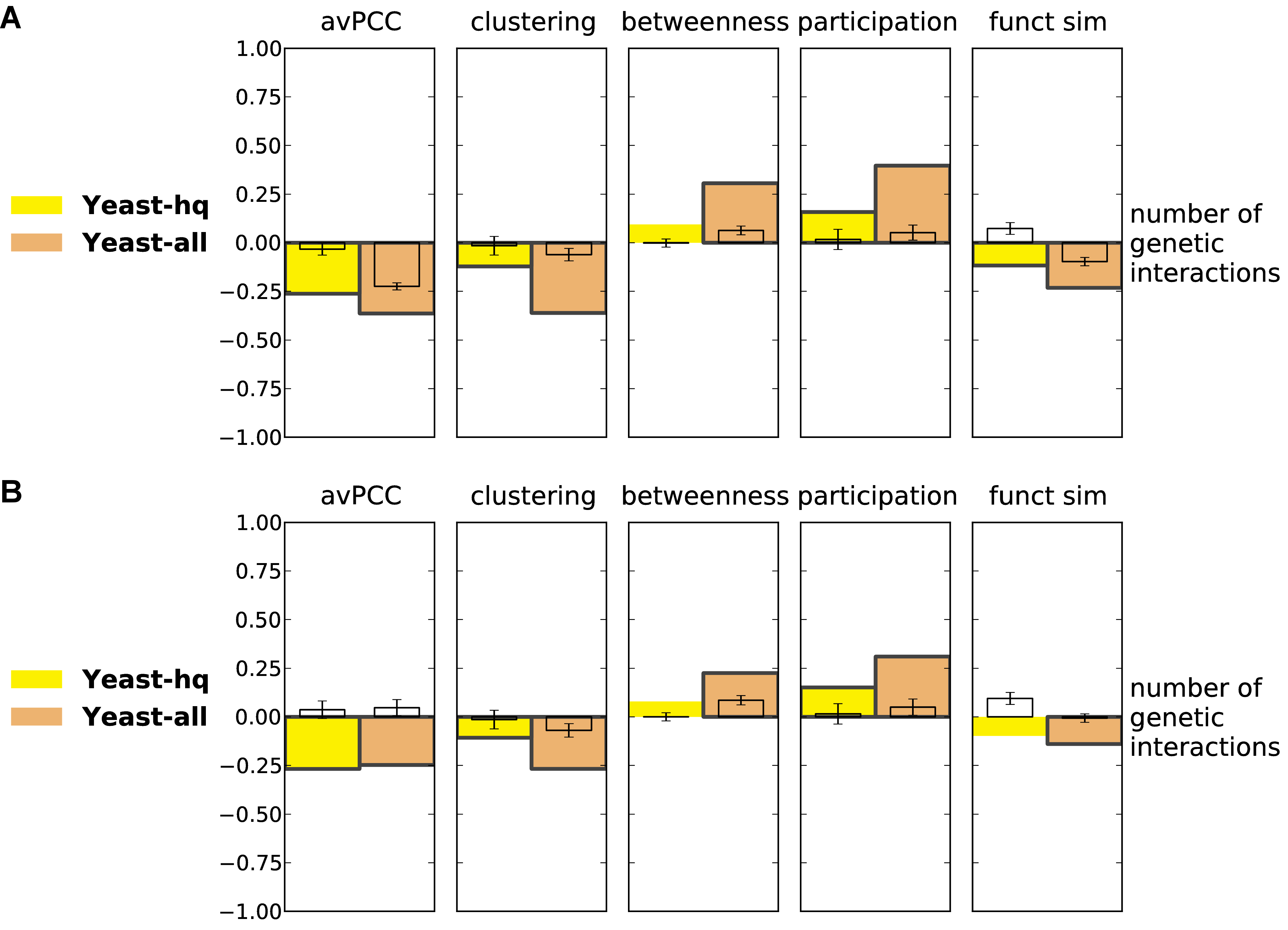

Supplement: Figure S34 — avPCC-rand is not a confounding factor in the correlation analysis of hub characteristics and genetic degree in yeast physical interaction networks. (A) Every bar represents a Spearman correlation between a hub characteristic and the number of genetic interactions for hubs in one of the yeast networks. Bars of significant correlations (absolute value , p-value) have black edges. (B) Every bar represents a partial Spearman correlation between a hub characteristic and the number of genetic interactions corrected for avPCC-rand for hubs in one of the yeast networks. Smaller uncolored bars show average correlation (with error bars for standard deviations) in 100 random networks on the same genes with the same number of interactions for each. Random networks used for the plot are different from those used for the calculation of avPCC-rand. (TIF) [file pcbi.1003243.s034.tif]

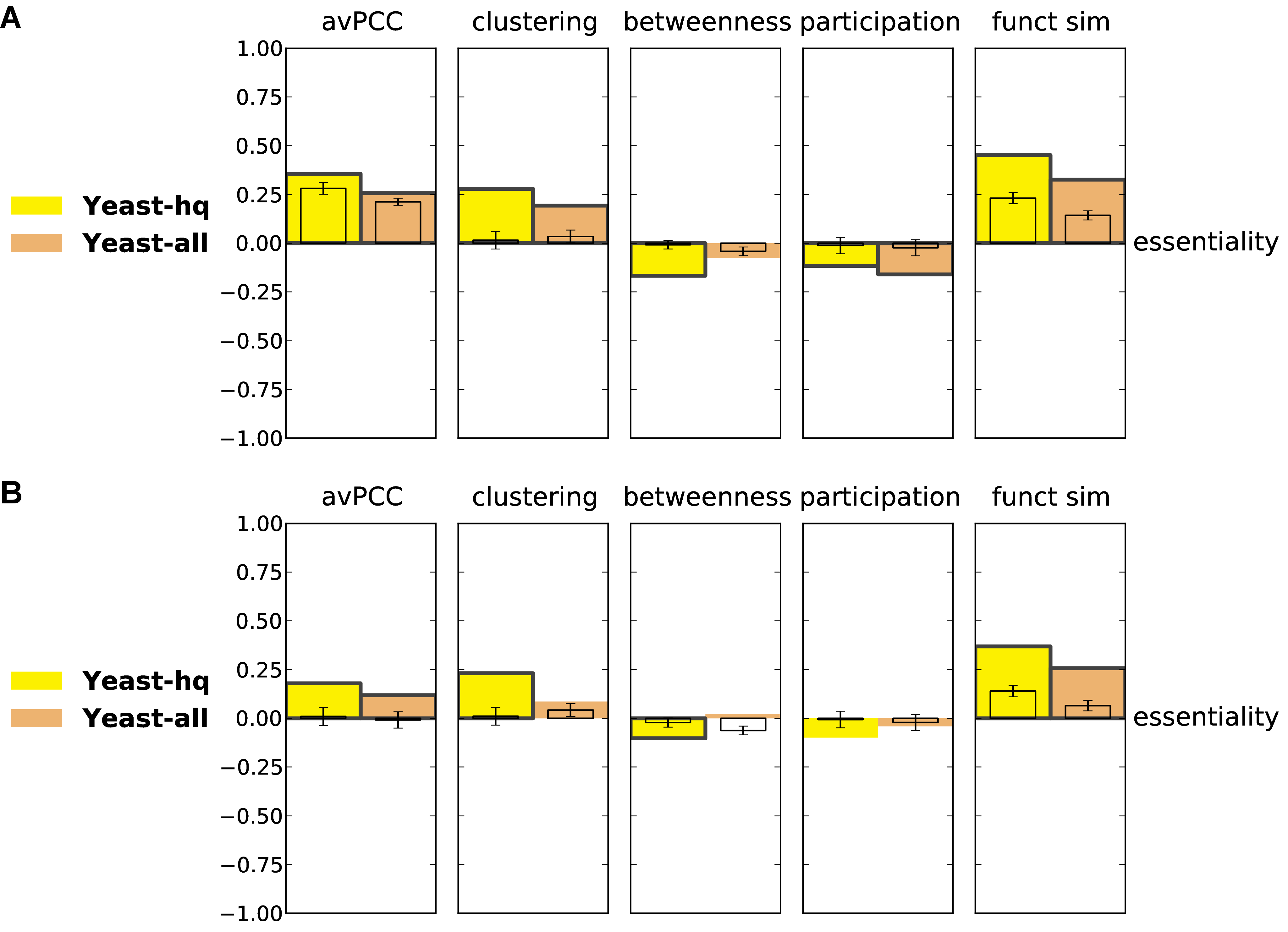

Supplement: Figure S35 — avPCC-rand is not a confounding factor in the correlation analysis of hub characteristics and essentiality in yeast physical interaction networks. (A) Every bar represents a Spearman correlation between a hub characteristic and essentiality (1 if essential, 0 otherwise) for hubs in one of the yeast networks. (B) Every bar represents a partial Spearman correlation between a hub characteristic and essentiality (1 if essential, 0 otherwise) corrected for avPCC-rand for hubs in one of the yeast networks. Bars of significant correlations (absolute value , p-value) have black edges. Smaller uncolored bars show average correlation (with error bars for standard deviations) in 100 random networks on the same genes with the same number of interactions for each. Random networks used for the plot are different from those used for the calculation of avPCC-rand. (TIF) [file pcbi.1003243.s035.tif]
