# Supplementary material for: Simple Topological Features Reflect Dynamics and Modularity in Protein Interaction Networks
Source: PLoS Comput Biol. 2013 Oct 10;9(10):e1003243. doi: 10.1371/journal.pcbi.1003243 (PMC3794914; doi:10.1371/journal.pcbi.1003243)
Supplement: Table S1 — Spearman correlation of avPCC with clustering, betweenness, participation and functional similarity of hubs in the network. (PDF) [file pcbi.1003243.s036.pdf]

Table S1. Spearman correlation of avPCC with clustering, betweenness, participation and functional similarity of hubs in the network.

	clustering	betweenness	participation	func. similarity
**Human-hq**	**0.61** (7e−48)	−**0.54** (1e−36)	−**0.59** (1e−45)	**0.59** (1e−44)
**Yeast-hq**	**0.39** (5e−18)	−**0.36** (6e−15)	−**0.30** (2e−10)	**0.42** (2e−20)
**Fly**	**0.51** (5e−59)	−**0.28** (3e−17)	−**0.42** (2e−37)	**0.50** (4e−55)
**Athal**	**0.31** (5e−13)	−**0.20** (5e−06)	−**0.26** (2e−09)	**0.25** (1e−08)
**Ecoli**	**0.30** (6e−08)	0.03 (6e−01)	−**0.30** (4e−08)	**0.30** (4e−08)
**Human-all**	**0.56** (4e−79)	−**0.42** (2e−40)	−**0.58** (2e−83)	**0.46** (4e−49)
**Yeast-all**	**0.72** (1e−91)	−**0.55** (2e−45)	−**0.69** (6e−82)	**0.56** (3e−49)

All correlations except one are significant ($p < 0.05$) and are shown in bold. avPCC is positively correlated with clustering coefficient and functional similarity, while negatively correlated with betweenness centrality and participation coefficient. See also Tables S2, S3, and S4.
