# Supplementary material for: Simple Topological Features Reflect Dynamics and Modularity in Protein Interaction Networks
Source: PLoS Comput Biol. 2013 Oct 10;9(10):e1003243. doi: 10.1371/journal.pcbi.1003243 (PMC3794914; doi:10.1371/journal.pcbi.1003243)
Supplement: Table S2 — Spearman correlation of clustering coefficient with betweenness, participation and functional similarity of hubs in the network. (PDF) [file pcbi.1003243.s037.pdf]

Table S2. Spearman correlation of clustering coefficient with betweenness, participation and functional similarity of hubs in the network.

	betweenness	participation	func. similarity
**Human-hq**	**−0.84** (4e−131)	**−0.85** (5e−135)	**0.71** (6e−75)
**Yeast-hq**	**−0.78** (1e−94)	**−0.78** (3e−93)	**0.57** (1e−39)
**Fly**	**−0.59** (1e−80)	**−0.86** (2e−250)	**0.29** (4e−18)
**Athal**	**−0.57** (6e−49)	**−0.55** (5e−46)	**0.31** (5e−14)
**Ecoli**	**−0.52** (5e−23)	**−0.84** (8e−85)	0.03 (6e−01)
**Human-all**	**−0.85** (5e−290)	**−0.96** (0)	**0.26** (6e−17)
**Yeast-all**	**−0.78** (9e−116)	**−0.92** (4e−234)	**0.68** (6e−78)

All correlations except one are significant ($p < 0.05$) and are shown in bold. See also Tables S1, S3 and S4.
