# Supplementary material for: Simple Topological Features Reflect Dynamics and Modularity in Protein Interaction Networks
Source: PLoS Comput Biol. 2013 Oct 10;9(10):e1003243. doi: 10.1371/journal.pcbi.1003243 (PMC3794914; doi:10.1371/journal.pcbi.1003243)
Supplement: Table S3 — Spearman correlation of betweenness centrality with participation and functional similarity of hubs in the network. (PDF) [file pcbi.1003243.s038.pdf]

Table S3. Spearman correlation of betweenness centrality with participation and functional similarity of hubs in the network.

	participation	func. similarity
**Human-hq**	**0.83** (2e−123)	−**0.62** (6e−52)
**Yeast-hq**	**0.60** (1e−44)	−**0.43** (7e−22)
**Fly**	**0.62** (2e−92)	−**0.09** (5e−03)
**Athal**	**0.51** (1e−38)	−**0.09** (3e−02)
**Ecoli**	**0.39** (6e−13)	0.09 (1e−01)
**Human-all**	**0.83** (1e−263)	−**0.19** (2e−09)
**Yeast-all**	**0.70** (1e−86)	−**0.54** (9e−44)

All correlations except one are significant ($p < 0.05$) and are shown in bold. See also Tables S1, S2 and S4.
