# Supplementary material for: Simple Topological Features Reflect Dynamics and Modularity in Protein Interaction Networks
Source: PLoS Comput Biol. 2013 Oct 10;9(10):e1003243. doi: 10.1371/journal.pcbi.1003243 (PMC3794914; doi:10.1371/journal.pcbi.1003243)
Supplement: Table S4 — Spearman correlation of participation coefficient with functional similarity. (PDF) [file pcbi.1003243.s039.pdf]

Table S4. Spearman correlation of participation coefficient with functional similarity.

	func. similarity
**Human-hq**	$-\mathbf{0.65}$ (1e−58)
**Yeast-hq**	$-\mathbf{0.27}$ (4e−09)
**Fly**	$-\mathbf{0.21}$ (3e−10)
**Athal**	$-\mathbf{0.21}$ (4e−07)
**Ecoli**	−0.03 (6e−01)
**Human-all**	$-\mathbf{0.27}$ (6e−19)
**Yeast-all**	$-\mathbf{0.59}$ (3e−54)

All correlations except one are significant ($p < 0.05$) and are shown in bold. See also Tables S1, S3 and S2.
