# Supplementary material for: Simple Topological Features Reflect Dynamics and Modularity in Protein Interaction Networks
Source: PLoS Comput Biol. 2013 Oct 10;9(10):e1003243. doi: 10.1371/journal.pcbi.1003243 (PMC3794914; doi:10.1371/journal.pcbi.1003243)
Supplement: Table S5 — Spearman correlation for characteristics of orthologous hubs in Yeast-hq and Human-hq . (PDF) [file pcbi.1003243.s040.pdf]

Table S5. Spearman correlation for characteristics of orthologous hubs in Yeast-hq and Human-hq.

characteristic	ρ	p-val	empirical p-val
avPCC	**0.23**	0.005	0.001
clustering	**0.62**	$7e{-}17$	< 0.001
betweenness	**0.53**	$6e{-}12$	< 0.001
participation	**0.58**	$2e{-}14$	< 0.001
func. sim	**0.49**	$4e{-}10$	< 0.001

Five hub characteristics for all 149 orthologous pairs between 109 hubs in **Yeast-hq** and 124 hubs in **Human-hq** are significantly positively correlated, as measured by Spearman's rho (ρ) and the correspondingly determined p-values and empirical p-values for 1000 random permutations of hubs. See main text and **Materials and methods** for details.
