# Supplementary material for: Simple Topological Features Reflect Dynamics and Modularity in Protein Interaction Networks
Source: PLoS Comput Biol. 2013 Oct 10;9(10):e1003243. doi: 10.1371/journal.pcbi.1003243 (PMC3794914; doi:10.1371/journal.pcbi.1003243)
Supplement: Table S6 — Spearman correlation of avPCC for orthologs between species. (PDF) [file pcbi.1003243.s041.pdf]

Table S6. Spearman correlation of avPCC for orthologs between species.

networks 1 and 2	ρ	p-val	empirical p-val	# or-tho-logs	# hubs org 1	# hubs org 2
**Athal** and **Fly**	**0.38**	$2e{-}06$	< 0.001	143	99	73
**Yeast-all** and **Athal**	**0.26**	0.005	0.004	115	48	67
**Yeast-hq** and **Fly**	**0.26**	0.002	0.001	136	100	117
**Athal** and **Human-all**	0.14	0.07	0.030	180	90	89
**Athal** and **Human-hq**	0.08	0.3	0.150	133	79	63
**Fly** and **Human-all**	0.02	0.7	0.362	316	201	235
**Yeast-all** and **Fly**	-0.06	0.4	0.215	182	139	134
**Yeast-hq** and **Athal**	-0.18	0.1	0.066	68	39	60

avPCC correlation analysis for hubs in pairs of networks: Spearman's rho, corresponding p-value, empirical p-value for 1000 random permutations of avPCC among hubs, the number of orthologous pairs of hubs and the numbers of hubs in each organism involved into orthologs (only for hubs with assigned avPCC score). Correlations with absolute value above 0.1 and both p-values < 0.05 are shown in bold. See main text and **Materials and methods** for details.
