# Supplementary material for: Simple Topological Features Reflect Dynamics and Modularity in Protein Interaction Networks
Source: PLoS Comput Biol. 2013 Oct 10;9(10):e1003243. doi: 10.1371/journal.pcbi.1003243 (PMC3794914; doi:10.1371/journal.pcbi.1003243)
Supplement: Table S7 — Spearman correlation of clustering coefficient for orthologs between species. (PDF) [file pcbi.1003243.s042.pdf]

Table S7. Spearman correlation of clustering coefficient for orthologs between species.

networks 1 and 2	ρ	p-val	empirical p-val
**Yeast-hq** and **Athal**	**0.35**	0.003	< 0.001
**Athal** and **Human-hq**	**0.34**	$6e{-}05$	0.001
**Fly** and **Human-all**	**0.28**	$7e{-}07$	< 0.001
**Yeast-hq** and **Fly**	**0.25**	0.003	0.003
**Athal** and **Human-all**	**0.23**	0.002	0.001
**Yeast-all** and **Athal**	**0.22**	0.02	0.012
**Yeast-all** and **Fly**	**0.17**	0.02	0.009
**Athal** and **Fly**	-0.03	0.7	0.343

Clustering coefficient correlation analysis for hubs in pairs of networks: Spearman's rho, corresponding p-value, empirical p-value for 1000 random permutations of clustering coefficient values among hubs. Correlations with absolute value above 0.1 and both p-values < 0.05 are shown in bold. See main text and **Materials and methods** for details.
