# Supplementary material for: Simple Topological Features Reflect Dynamics and Modularity in Protein Interaction Networks
Source: PLoS Comput Biol. 2013 Oct 10;9(10):e1003243. doi: 10.1371/journal.pcbi.1003243 (PMC3794914; doi:10.1371/journal.pcbi.1003243)
Supplement: Table S8 — Spearman correlation of betweenness centrality for orthologs between species. (PDF) [file pcbi.1003243.s043.pdf]

Table S8. Spearman correlation of betweenness centrality for orthologs between species.

networks 1 and 2	ρ	p-val	empirical p-val
**Yeast-all** and **Athal**	**0.42**	$3e{-}06$	< 0.001
**Athal** and **Human-hq**	**0.38**	$7e{-}06$	< 0.001
**Athal** and **Human-all**	**0.33**	$6e{-}06$	< 0.001
**Yeast-hq** and **Athal**	**0.37**	0.002	0.001
**Fly** and **Human-all**	**0.27**	$8e{-}07$	< 0.001
**Yeast-hq** and **Fly**	**0.26**	0.002	0.001
**Yeast-all** and **Fly**	0.11	0.1	0.067
**Athal** and **Fly**	−0.00	1.0	0.493

Betweenness centrality correlation analysis for hubs in pairs of networks: Spearman's rho, corresponding p-value, empirical p-value for 1000 random permutations of betweenness centrality values among hubs. Correlations with absolute value above 0.1 and both p-values < 0.05 are shown in bold. See main text and **Materials and methods** for details.
