# Supplementary material for: Simple Topological Features Reflect Dynamics and Modularity in Protein Interaction Networks
Source: PLoS Comput Biol. 2013 Oct 10;9(10):e1003243. doi: 10.1371/journal.pcbi.1003243 (PMC3794914; doi:10.1371/journal.pcbi.1003243)
Supplement: Table S9 — Spearman correlation of participation coefficient for orthologs between species. (PDF) [file pcbi.1003243.s044.pdf]

Table S9. Spearman correlation of participation coefficient for orthologs between species.

networks 1 and 2	ρ	p-val	empirical p-val
**Fly** and **Human-all**	**0.19**	$9e{-}04$	< 0.001
**Yeast-hq** and **Athal**	0.18	0.2	0.090
**Yeast-all** and **Athal**	0.15	0.1	0.043
**Athal** and **Human-all**	0.09	0.2	0.105
**Athal** and **Human-hq**	0.11	0.2	0.093
**Yeast-hq** and **Fly**	0.03	0.8	0.391
**Yeast-all** and **Fly**	-0.03	0.8	0.395
**Athal** and **Fly**	0.02	0.9	0.436

Participation coefficient correlation analysis for hubs in pairs of networks: Spearman's rho, corresponding p-value, empirical p-value for 1000 random permutations of participation coefficient values among hubs. Correlations with absolute value above 0.1 and both p-values < 0.05 are shown in bold. See main text and **Materials and methods** for details.
