# Supplementary material for: Simple Topological Features Reflect Dynamics and Modularity in Protein Interaction Networks
Source: PLoS Comput Biol. 2013 Oct 10;9(10):e1003243. doi: 10.1371/journal.pcbi.1003243 (PMC3794914; doi:10.1371/journal.pcbi.1003243)
Supplement: Table S10 — Spearman correlation of functional similarity for orthologs between species. (PDF) [file pcbi.1003243.s045.pdf]

Table S10. Spearman correlation of functional similarity for orthologs between species.

networks 1 and 2	ρ	p-val	empirical p-val
**Yeast-hq** and **Fly**	**0.37**	$2e{-}05$	< 0.001
**Fly** and **Human-all**	**0.25**	$8e{-}06$	< 0.001
**Athal** and **Fly**	**0.24**	$4e{-}03$	0.004
**Yeast-all** and **Athal**	**0.19**	0.04	0.022
**Yeast-all** and **Fly**	0.12	0.1	0.055
**Athal** and **Human-all**	0.05	0.5	0.211
**Yeast-hq** and **Athal**	−0.02	0.9	0.424
**Athal** and **Human-hq**	−0.06	0.5	0.260

Functional similarity correlation analysis for hubs in pairs of networks: Spearman's rho, corresponding p-value, empirical p-value for 1000 random permutations of functional similarity values among hubs. Correlations with absolute value above 0.1 and both p-values < 0.05 are shown in bold. See main text and **Materials and methods** for details.
