# Supplementary material for: Simple Topological Features Reflect Dynamics and Modularity in Protein Interaction Networks
Source: PLoS Comput Biol. 2013 Oct 10;9(10):e1003243. doi: 10.1371/journal.pcbi.1003243 (PMC3794914; doi:10.1371/journal.pcbi.1003243)
Supplement: Table S11 — Fraction of hubs annotated with GO terms in each network. (PDF) [file pcbi.1003243.s046.pdf]

Table S11. Fraction of hubs annotated with GO terms in each network.

network	BP	CC	MF	none
**Human-hq**	97	96	74	3
**Yeast-hq**	92	96	71	2
**Fly-hq**	68	63	66	21
**Athal**	71	58	69	13
**Ecoli**	48	39	49	35
**Human-all**	85	86	61	10
**Yeast-all**	94	98	79	0

For each network and for each ontology (Biological process, Cellular component, Molecular function), we show the percentage of hubs annotated with at least some terms other than the root (the most general) term in this ontology (BP, CC, MF), and the percentage of hubs not annotated in any of the three ontologies (none).
