# Supplementary material for: Simple Topological Features Reflect Dynamics and Modularity in Protein Interaction Networks
Source: PLoS Comput Biol. 2013 Oct 10;9(10):e1003243. doi: 10.1371/journal.pcbi.1003243 (PMC3794914; doi:10.1371/journal.pcbi.1003243)
Supplement: Table S12 — Interaction evidence types from different sources used for interaction annotation. (PDF) [file pcbi.1003243.s047.pdf]

Table S12. Interaction evidence types from different sources used for interaction annotation.

	yeast two-hybrid	co-complex
BioGRID	Two-hybrid	Affinity Capture-Luminescence<br>Affinity Capture-MS<br>Affinity Capture-RNA<br>Affinity Capture-Western<br>Co-purification<br>Reconstituted Complex
IntAct	two hybrid	tandem affinity purification<br>pull down<br>anti tag coimmunoprecipitation<br>anti bait coimmunoprecipitation<br>affinity chromatography technology
Bossi and Lehner 2009	two_hybrid<br>-two_hybrid<br>two_hybrid_test<br>yeast<br>two_hybridarray<br>two_hybridpooling<br>sd4-two_hybrid<br>lacz4-two_hybrid<br>two_h	affinitycapture_ms<br>affin<br>gst_pulldown<br>affinity<br>mass_spectrometry<br>indirect_complex<br>reconstitutedcomplex<br>affinity_tag<br>affinity_chromatography<br>co_purification<br>direct_complex<br>copurification<br>affinity_c<br>affinity_chrom<br>tandem_affinitypurification<br>tap<br>literature_annotated_complex<br>affinity_techniques<br>affinity_co_purification<br>affinitycapture_western<br>affinit<br>pull_down

Note that evidence types from [Bossi and Lehner, *Mol Sys Biol* 5:260 (2009)] are not organized in any formal vocabulary and were parsed from Column 3 of the table with interactions using symbols |, (,), and : as punctuation.
