# Supplementary material for: Simple Topological Features Reflect Dynamics and Modularity in Protein Interaction Networks
Source: PLoS Comput Biol. 2013 Oct 10;9(10):e1003243. doi: 10.1371/journal.pcbi.1003243 (PMC3794914; doi:10.1371/journal.pcbi.1003243)
Supplement: Table S13 — Datasets used in S. cerevisiae expression compendium. (PDF) [file pcbi.1003243.s048.pdf]

Table S13. Datasets used in *S. cerevisiae* expression compendium

GEO accession number	publication or submission information	# datapoints
GSE29894	Cell cycle and G1 cyclins. Public on Jun 11, 2011. Skotheim Lab, Stanford `http://web.me.com/skotheim/Site/People.html`	32
GSE22904	Lewis JA, Elkon IM, McGee MA, Higbee AJ et al. Exploiting natural variation in Saccharomyces cerevisiae to identify genes for increased ethanol resistance. Genetics 2010 Dec;186(4):1197-205. PMID: 20855568	18
GSE23204	The Role of the Rad4-Rad23 Complex and Rad4 Ubiquitination in UV-Responsive Transcription. Public on Aug 02, 2010. Humphryes N, Reed S. Cardiff University School of Medicine	12
GSE22458	Bermejo C, Garca R, Straede A, Rodrguez-Pea JM et al. Characterization of sensor-specific stress response by transcriptional profiling of wsc1 and mid2 deletion strains and chimeric sensors in Saccharomyces cerevisiae. OMICS 2010 Dec;14(6):679-88. PMID: 20958245	10
GSE15254	Staschke KA, Dey S, Zaborske JM, Palam LR et al. Integration of general amino acid control and target of rapamycin (TOR) regulatory pathways in nitrogen assimilation in yeast. J Biol Chem 2010 May 28;285(22):16893-911. PMID: 20233714	18
GSE15147	Eng KH, Kvitek DJ, Keles S, Gasch AP. Transient genotype-by-environment interactions following environmental shock provide a source of expression variation for essential genes. Genetics 2010 Feb;184(2):587-93. PMID: 19966067	34
GSE18121	Gene expression regulation in response to heat stress in different yeast strains. Public on Nov 09, 2009. Cowart LA, Lu X, Hannun Y. Medical University of South Carolina	21
GSE13653	Halbeisen RE, Gerber AP. Stress-Dependent Coordination of Transcriptome and Translatome in Yeast. PLoS Biol 2009 May 5;7(5):e105. PMID: 19419242	12
GSE8335	Berry DB, Gasch AP. Stress-activated genomic expression changes serve a preparative role for impending stress in yeast. Mol Biol Cell 2008 Nov;19(11):4580-7. PMID: 18753408	32
GSE7645	Expression data for Saccharomyces cerevisiae oxidative stress response. Public on Oct 24, 2007. Sha W, Martins A, Laubenbacher R, Mendes P, Shulaev V. Virginia Bioinformatics Institute	16
GSE7362	The contribution of different nutrients to spore germination in Saccharomyces cerevisiae. Joseph-Strauss D, Zenvirth D, Simchen G, Barkai N. Spore germination in Saccharomyces cerevisiae: global gene expression patterns and cell cycle landmarks. Genome Biol 2007;8(11):R241. PMID: 17999778	38
GSE7358	Spore Germination in Saccharomyces cerevisiae : transfer of wild type spores to rich (YPD) medium. Joseph-Strauss D, Zenvirth D, Simchen G, Barkai N. Spore germination in Saccharomyces cerevisiae: global gene expression patterns and cell cycle landmarks. Genome Biol 2007;8(11):R241. PMID: 17999778	31
GSE12270	Capaldi AP, Kaplan T, Liu Y, Habib N et al. Structure and function of a transcriptional network activated by the MAPK Hog1. Nat Genet 2008 Nov;40(11):1300-6. PMID: 18931682	29
GSE4987	Pramila T, Wu W, Miles S, Noble WS et al. The Forkhead transcription factor Hcm1 regulates chromosome segregation genes and fills the S-phase gap in the transcriptional circuitry of the cell cycle. Genes Dev 2006 Aug 15;20(16):2266-78. PMID: 16912276	25
GSE5376	Cell cycle of yeast deleted for yox1. Public on Sep 30, 2007. Pramila T, Breeden LL. Breeden Lab, FHCRC	25

GSE6302	Levy S, Ihmels J, Carmi M, Weinberger A et al. Strategy of transcription regulation in the budding yeast. PLoS One 2007 Feb 28;2(2):e250. PMID: 17327914	92
GSE8825	Brauer MJ, Huttenhower C, Airoldi EM, Rosenstein R et al. Coordination of growth rate, cell cycle, stress response, and metabolic activity in yeast. Mol Biol Cell 2008 Jan;19(1):352-67. PMID: 17959824	36
GSE8982	Mating response — six alpha factor concentrations (0.06, 0.2, 0.6, 6, 60 and 600 nM). Public on Sep 11, 2007. Barkai Lab, Department of Molecular Genetics, Weizmann Institute of Science	33
GSE10521	Azzouz N, Panasenko OO, Deluen C, Hsieh J et al. Specific roles for the Ccr4-Not complex subunits in expression of the genome. RNA 2009 Mar;15(3):377-83. PMID: 19155328	14
GSE11397	Willis IM, Chua G, Tong AH, Brost RL et al. Genetic interactions of MAF1 identify a role for Med20 in transcriptional repression of ribosomal protein genes. PLoS Genet 2008 Jul 4;4(7):e1000112. PMID: 18604275	12
