# Supplementary material for: Simple Topological Features Reflect Dynamics and Modularity in Protein Interaction Networks
Source: PLoS Comput Biol. 2013 Oct 10;9(10):e1003243. doi: 10.1371/journal.pcbi.1003243 (PMC3794914; doi:10.1371/journal.pcbi.1003243)
Supplement: Table S14 — Datasets used in D. melanogaster expression compendium. (PDF) [file pcbi.1003243.s049.pdf]

Table S14. Datasets used in *D. melanogaster* expression compendium

GEO accession number	publication or submission information	# datapoints
GSE7763	FlyAtlas `http://flyatlas.org`. Chintapalli VR, Wang J, Dow JA. Using FlyAtlas to identify better Drosophila melanogaster models of human disease. Nat Genet 2007 Jun;39(6):715-20. PMID: 17534367	30
GSE6186	Whole Genome Drosophila Embryogenesis Time Course. Public on Nov 02, 2006. Hooper SD, Boue S, Krause R, Jensen LJ, Mason CE, Ghanim M, Furlong EE, White KP, Bork P. State Lab, Department of Genetics, Yale University	28
GSE5430	Qin X, Ahn S, Speed TP, Rubin GM. Global analyses of mRNA translational control during early Drosophila embryogenesis. Genome Biol 2007;8(4):R63. PMID: 17448252	39
GSE8892	Abundant genetic variation in transcript level during early Drosophila development. Public on Aug 30, 2007. Nuzhdin SV, Tufts DM, Hahn MW. Department of Biology, Indiana University	18
GSE13303	Gene Expression during the Egg Development of Drosophila melanogaster. Baker DA, Russell S. Department of Genetics, University of Cambridge	12
GSE22354	Pavlopoulos A, Akam M. Hox gene Ultrabithorax regulates distinct sets of target genes at successive stages of Drosophila haltere morphogenesis. Proc Natl Acad Sci U S A 2011 Feb 15;108(7):2855-60. PMID: 21282633	12
GSE20497	Menin links the stress response to genome stability in Drosophila melanogaster. Razak Z. Canadian Drosophila Microarray Centre `http://www.flyarrays.com`	12
GSE5147	Sørensen JG, Nielsen MM, Kruhøffer M, Justesen J et al. Full genome gene expression analysis of the heat stress response in Drosophila melanogaster. Cell Stress Chaperones 2005 Winter;10(4):312-28. PMID: 16333985	18
modENCODE	Celniker SE, Dillon LA, Gerstein MB, Gunsalus KC, Henikoff S, Karpen GH, Kellis M, Lai EC, Lieb JD, MacAlpine DM, Micklem G, Piano F, Snyder M, Stein L, White KP, Waterston RH; modENCODE Consortium. Unlocking the secrets of the genome. Nature. 2009 Jun 18;459(7249):927-30. PMID: 19536255. As analyzed and published by FlyBase `http://flybase.org` at FlyBase High Throughput Expression Pattern Data Beta Version, FBrf0212041 (2010.10.13) `http://flybase.org/reports/FBrf0212041.html`	30
