# Supplementary material for: Simple Topological Features Reflect Dynamics and Modularity in Protein Interaction Networks
Source: PLoS Comput Biol. 2013 Oct 10;9(10):e1003243. doi: 10.1371/journal.pcbi.1003243 (PMC3794914; doi:10.1371/journal.pcbi.1003243)
Supplement: Text S1 — Simple topological features reflect dynamics and modularity in protein interaction networks. (PDF) [file pcbi.1003243.s050.pdf]

Supporting Text S1 for

Simple topological features reflect dynamics and modularity in protein interaction networks

Yuri Pritykin[1] and Mona Singh*[1]

[1]Princeton University, Princeton, New Jersey, USA

August 28, 2013

Contents

S1 Supplementary Results **1**
- S1.1 Hub classification analysis . . . 1
- S1.2 Analysis for a relaxed definition of hubs . . . 2
- S1.3 Potential biases and confounding factors in the correlation analysis of hub characteristics . . . 2
- S1.4 GO annotations of hubs . . . 2
- S1.5 Correction for essentiality when studying the number of genetic interactions of genes . . . 3
- S1.6 Yeast two-hybrid and co-complex interaction networks . . . 3
- S1.7 Comparison of network topology properties for orthologs between organisms . . . 3
- S1.8 Correction for signal from random networks for genetic interactions and essentiality 3

S2 Supplementary Materials and Methods **4**
- S2.1 Gene IDs . . . 4
- S2.2 Interactions . . . 5
- S2.3 Expression datasets . . . 6
- S2.4 Clustering the network for computing participation coefficient . . . 6

S1 Supplementary Results

S1.1 Hub classification analysis

Our classification of hubs into party, date and extremal for different networks (Fig. S1–S6) yields results qualitatively similar to those reported in the main text for the **Human-hq** network (Fig. 1).

*E-mail: mona@cs.princeton.edu

The results of classification of all hubs are qualitatively the same as the results of classification with extremal hubs excluded (Fig. S7, compare with Fig. 1).

S1.2 Analysis for a relaxed definition of hubs

For the three largest networks, we also considered a more relaxed definition of hubs, where all genes with degree ≥ 3 are considered as hubs, instead of just the top 10%. This results in 6762 hubs in **Human-all** (66.1% of all vertices), 4716 (83.6%) hubs in **Yeast-all** and 4992 (60.7%) hubs in **Fly**. Results of our hub classification analyses are largely the same as for hubs defined with a more selective definition (Fig. S8–S10). Furthermore, the results of correlation analysis of hub characteristics stay largely the same as with a more selective definition (Fig. S11).

We do however observe a higher betweenness for party hubs in **Yeast-all** when considering as hubs all genes of degree ≥ 3 (Fig. S9), which is the opposite trend of when a higher hub threshold is used. This may be explained by the correlation of degree with avPCC in this case, and the typically observed correlation between degree and betweenness. Indeed, the SRCC between degree and avPCC is 0.32 ($p < 1e{-}110$), the SRCC between degree and betweenness is 0.81 ($p = 0$), and the SRCC between avPCC and betweenness is 0.21 ($p < 9e{-}46$). However, the partial SRCC of avPCC and betweenness corrected for degree is -0.10 ($p < 3e{-}11$), which is consistent with all previous observations (Fig. S12).

S1.3 Potential biases and confounding factors in the correlation analysis of hub characteristics

The number of interactions of a protein in the network could be significantly correlated with avPCC and other topological measures, and this may be a confounding factor in the analysis [1]. Sometimes we indeed observe a correlation (Fig. S13A). To control for this, we calculate the Spearman partial correlation with a correction for degree. High correlations of hub characteristics remain significant (see Fig. S13B and compare with Fig. 2).

In order to show that hubs with extremal properties do not bias the analysis of correlations between hub features, we perform the same analysis, but with extremal hubs excluded, and observe very similar results (see Fig. S14 and compare with Fig. 2).

A bias towards more studied genes could also be responsible for some of the observed correlations [1]. In order to avoid that, we also perform the correlation analysis on high-throughput networks for yeast and human and observe the same trends as for our main networks (see Fig. S15, compare with Fig. 2).

S1.4 GO annotations of hubs

We performed GO enrichment analysis for date and party hubs, as well as for classes of hubs specified by other hub characteristics. These results are shown in Fig. 3 in the main text and in Fig. S16–S21. See **Materials and methods** in the main text for details.

For the **Fly**, **Athal** and **Ecoli** networks, we observe results that are in general similar to those for the human and yeast networks, though fewer terms are enriched. A possible explanation for the fewer number of enriched terms may be that hubs in these networks have fewer annotations than hubs in the networks of yeast and human. We show in Table S11 the fraction of hubs

annotated with terms other than the root in each ontology, and these numbers are considerably smaller for **Fly**, **Athal** and **Ecoli** than for the yeast and human networks.

S1.5 Correction for essentiality when studying the number of genetic interactions of genes

Non-essential genes participate in a significantly larger number of genetic interactions than essential genes, or in other words, essentiality is correlated with the number of genetic interactions (Fig. S24A). However, even after removing all essential genes from consideration, the numbers of genetic interactions date and party hubs are involved in are still significantly different (Fig. S22CD). The partial correlation of avPCC and the number of genetic interactions with correction for essentiality is almost as high as without correction (see Fig. S24B and compare with Fig. 4 in the main text).

S1.6 Yeast two-hybrid and co-complex interaction networks

The date and party hub analysis on networks of only yeast two-hybrid or only co-complex interactions for *H. sapiens*, *S. cerevisiae*, and *A. thaliana* (Fig. S26–S31) confirms that the date/party distinction is observable in these networks as well, though it is not as stringent for yeast two-hybrid as it is for co-complex networks.

S1.7 Comparison of network topology properties for orthologs between organisms

We compute the Spearman correlation of various hub characteristics across networks. The results for networks **Human-all** and **Yeast-all** are shown in the main text (Table 2), the results for networks **Human-hq** and **Yeast-hq** are in Table S5, and the results for the other networks are in Tables S6–S9 (organized per hub feature, rather than per a pair of networks). We observe that clustering coefficient, as well as betweenness centrality and participation coefficient, are highly correlated for networks of different organisms; this suggests that the placement and role of proteins within networks tend to be conserved and are biologically meaningful properties. Surprisingly, we do not observe the degree in the network, which is simply the number of physical interactions, to correlate in most cases: the only significant correlations were $\rho = 0.23$ ($p < 0.01$; empirical $p = 0.006$) for **Yeast-all** and **Athal**, $\rho = 0.14$ ($p < 0.003$; empirical $p = 0.002$) for **Yeast-all** and **Human-all**; this may be due to which proteins are studied more extensively in different networks.

S1.8 Correction for signal from random networks for genetic interactions and essentiality

For correlations between hub characteristics (Fig. 2), we compared real correlations with those observed in random networks (as reported in the main text). We also perform the same analysis for correlations between hub characteristics in yeast and the number of genetic interactions. That is, for the yeast networks, we compute the average correlation of the number of genetic interactions with avPCC, clustering, betweenness, participation and functional similarity in 100 random

networks generated to preserve the number of physical interactions for each gene (see Fig. S34A and compare with Fig. 4 in the main text which shows the same bars for real networks). In all cases, correlations in random networks are smaller by absolute value than significant correlations in real networks.

We noticed, however, a surprisingly strong significant negative correlation of avPCC and genetic degree in random counterparts of the network **Yeast-all**. We noticed no such relationship when the network was randomized and the degree distribution was not preserved (data not shown). We hypothesized that if the degrees of genes in random networks are restricted to be the same as in the real physical interaction network, certain properties of the resulting randomized networks may preserve structures and correlations in ways that are not entirely understood. For example, we observed that hubs have a preference to interact with the same genes they interact with in real networks when performing degree-preserving randomizations. This may result in a positive correlation between avPCC in a random network and avPCC in the real network, that leads to correlations between avPCC in the random network and other traits (such as genetic interaction degree). To correct for the behavior of avPCC in random networks, we generate another 100 random networks (separately from those used for the plots) and compute for each hub the average of avPCC scores in these networks. We denote the resulting hub score as avPCC-rand. We confirm a positive Spearman correlation between avPCC in the real network and avPCC-rand (0.60 in **Yeast-hq** and 0.74 in **Yeast-all**), though there is a large difference in the magnitude of avPCC and avPCC-rand (mean of avPCC 0.14 vs mean of avPCC-rand 0.03 over all hubs in **Yeast-hq**, and mean of avPCC 0.16 vs mean of avPCC-rand 0.04 over all hubs in **Yeast-all**). Then, we compute partial Spearman correlations of the genetic degree and avPCC, clustering, betweenness, participation, and functional similarity corrected for avPCC-rand, and the same values computed in random networks (Fig. S34B, compare with Fig. S34A). The correlation between avPCC and the genetic degree is significant even after this correction, and is close to zero in random networks. Note again that random networks used for plots are different from those used to calculate avPCC-rand.

We also perform the same analysis for essentiality and obtain similar results (Fig. S35); that is, there is a significant correlation of essentiality and other hub features including avPCC even after correction for avPCC from random networks.

S2 Supplementary Materials and Methods

S2.1 Gene IDs

The following gene names were used as identifiers in networks and expression datasets.

S. cerevisiae: locus names such as YDL229W or YLR438C-A.

H. sapiens: Ensembl gene ids such as ENSG00000008988 or ENSG00000141510.

D. melanogaster: locus names such as CG14228 or CG9986.

A. thaliana: locus names such as AT1G66410 or AT5G42190.

E. coli: locus names such as B0015 or B4142.

All other gene identifiers were mapped to these using files from Saccharomyces Genome

Database (SGD)[1], Profiling of Escherichia coli chromosome (PEC) database[2], EcoCyc project[3], Arabidopsis Information Resource (TAIR)[4], Database of Drosophila Genes & Genomes (FlyBase)[5], Drosophila Interactions Database (DroID)[6], and gene mapping files downloaded using BioMart MartView interface[7] for different organisms.

S2.2 Interactions

The following interaction networks for five organisms are considered. In all networks, self-loops and duplicate interactions were deleted. The size of each network is shown in Table 1 from the main text.

S. cerevisiae: Based on evidence types from BioGRID, interactions in **Yeast-all** were annotated as 'yeast two-hybrid' (7810 in **Yeast-all**) and 'co-complex' (44610 in **Yeast-all**), see Table S12. Annotations of genetic interactions were taken from BioGRID evidence types: `Negative Genetic`, `Synthetic Growth Defect`, `Synthetic Haploinsufficiency`, `Synthetic Lethality` for negative (96142 interactions in total) and `Positive Genetic`, `Synthetic Rescue` for positive (20068 interactions).

H. sapiens: Based on evidence types of interactions from [2], in the network **Human-all** 14633 interactions were annotated as 'yeast two-hybrid' and 50390 were annotated as 'co-complex', see Table S12.

D. melanogaster: The network of physical protein-protein interactions **Fly** was obtained by combining all interactions from DroID [3] version 2011_02 (25948 interactions, annotated 'yeast two-hybrid'), and from DPiM [4] (10623 coAP-MS interactions reported as high-quality in the publication, annotated 'co-complex').

A. thaliana: The network of protein-protein interactions **Athal** was formed from datasets downloaded from IntAct [5] and BioGRID, as well as from the recent publication [6]. First, 4707 interactions were obtained from BioGRID represented by 881 publications, then from IntAct 2620 interactions were obtained from those 272 publications (out of total of 603) that were not present in BioGRID, in order to avoid duplicate representation of interactions from the same publications with different gene ids. These interactions were annotated as 'yeast two-hybrid' (3086 interactions) and 'co-complex' (3148 interactions) based on evidence types provided by BioGRID and IntAct. All 6045 non-redundant interactions from [6], AI-1 dataset, were annotated as 'yeast two-hybrid', see Table S12.

E. coli: The network of physical protein-protein interactions **Ecoli** was collected from different databases via PSICQUIC View application [7] using query `(taxidA:83333 AND taxidB:83333) AND (type:physical OR detmethod:(biophysical OR biochemical OR "two hybrid" OR affinity OR "pull down"))`. This network consists mostly of co-complex data.

---

[1] `SGD_features.tab` from `http://www.yeastgenome.org/`

[2] `PECData.dat` from `http://www.shigen.nig.ac.jp/ecoli/pec/`

[3] `gene-links.dat` from `http://ecocyc.org/ecocyc/index.shtml`

[4] `gene_aliases.20101027` from `http://www.arabidopsis.org/`

[5] `gene_map_table_fb_2011_06.tsv.gz` and `fbgn_annotation_ID_fb_2011_06.tsv.gz` from `http://flybase.org/`

[6] `FLY_GENE_ATTR.txt` from `http://www.droidb.org/`

[7] `http://www.biomart.org/biomart/martview`

S2.3 Expression datasets

The expression compendia for the five organisms are as follows:

H. sapiens: the GNF Atlas project data over 79 cell or tissue types [8] (downloaded from GEO, accession number GDS596) is used as the source of expression data for human.

S. cerevisiae: In the GEO [9] database, aiming to construct an unbiased representative expression compendium and following the approach of Han *et al.* [1], we searched for keywords "stimulus response OR stress response OR cell cycle" while limiting the search to Series data (GSE) having from 20 to 100 datapoints (upper limit to avoid bias from large datasets), and publication date from 2006/07 to the 2011/07 (corresponding to the five years directly prior to when we gathered this data). We took only genome-wide datasets that used only *S. cerevisiae* in microarray experiments, and only those that after merging replicates would provide at least 10 datapoints. This resulted in a compendium of 20 datasets with the total of 540 expression datapoints (see Table S13).

D. melanogaster: An expression compendium was formed from a collection of GEO datasets as was done for yeast (see above). Genome-wide RNA-seq data from the modENCODE project [10], as analyzed and published by FlyBase [11, 12], was added as well. This resulted in the compendium of 9 datasets with a total of 199 datapoints, from different types of cells including embryonic and various adult fly tissues, and under different conditions including development and stress response (see Table S14).

A. thaliana: A compendium consisting of development data [13] (79 datapoints, from various tissues) and stress response data [14] (149 datapoints, from cells from roots and shoots, as well as from cell cultures) from AtGenExpress project was formed. Expression datasets were downloaded from the web page of the project[8].

E. coli: An expression compendium of 362 datapoints was formed from two smaller ones: a dataset consisting of 240 datapoints from different conditions with several timepoints for each was obtained from [15] as a log ratio data file, and the dataset of 122 datapoints corresponding to different conditions [16] was obtained from GEO, accession number GSE6836.

S2.4 Clustering the network for computing participation coefficient

In order to compute the participation coefficient for hubs in a protein-protein interaction network, we first had to find clusters in the network. For this, we used the SPICi clustering algorithm [17] with parameters optimized with a simple exhaustive search procedure to approximately maximize Newman's modularity [18].

Namely, SPICi has two main parameters: the minimum density threshold parameter d and the minimum increment ratio r. We run SPICi many times with different parameters, and optimize for the resulting value of modularity. At the first stage we run the algorithm with parameters $d = 0.2, 0.4, 0.6, 0.8, 1.0$ and $r = d$ and select the preliminary value of $d = d_0$ that produces the maximum modularity. Then we do a binary search for the optimal value of d in the segment $[d_0 - 0.14, d_0 + 0.14]$ with granularity $1/2^{15}$, and for each hypothetical value of d, we optimize r in the segment $[0, d]$ with a step $d/15$.

[8] `http://www.weigelworld.org/resources/microarray/AtGenExpress/`

This method produces, for example, parameters $d = r = 0.09487$ resulting in a Newman's modularity measure of 0.573881 for the network **Human-hq**, or parameters $d = r = 0.20215$ resulting in a modularity measure of 0.253280 for **Yeast-all**.

References

1. Han JJ, Bertin N, Hao T, et al. (2004) Evidence for dynamically organized modularity in the yeast protein-protein interaction network. Nature 430: 88–93.

2. Bossi A, Lehner B (2009) Tissue specificity and the human protein interaction network. Molecular systems biology 5: 260.

3. Murali T, Pacifico S, Yu J, Guest S, Roberts GG, et al. (2011) DroID 2011: a comprehensive, integrated resource for protein, transcription factor, RNA and gene interactions for Drosophila. Nucleic Acids Research 39: D736-D743.

4. Guruharsha KG, Rual JF, Zhai B, Mintseris J, Vaidya P, et al. (2011) A protein complex network of *Drosophila melanogaster*. Cell 147: 690–703.

5. Aranda B, Achuthan P, Alam-Faruque Y, et al. (2010) The IntAct molecular interaction database in 2010. Nucleic Acids Research 38(suppl. 1): D525–D531.

6. *Arabidopsis* Interactome Mapping Consortium (2011) Evidence for Network Evolution in an *Arabidopsis* Interactome Map. Science 333: 601-607.

7. Aranda B, Blankenburg H, Kerrien S, Brinkman FSL, Ceol A, et al. (2011) PSICQUIC and PSISCORE: accessing and scoring molecular interactions. Nature Methods 8: 528-529.

8. Su AI, Wiltshire T, Batalov S, Lapp H, Ching KA, et al. (2004) A gene atlas of the mouse and human protein-encoding transcriptomes. Proceedings of the National Academy of Sciences of the United States of America 101: 6062–6067.

9. Barrett T, Troup DB, Wilhite SE, Ledoux P, Evangelista C, et al. (2011) NCBI GEO: archive for functional genomics data sets—10 years on. Nucleic Acids Research 39: D1005-D1010.

10. Celniker SE, Dillon LAL, Gerstein MB, Gunsalus KC, Henikoff S, et al. (2009) Unlocking the secrets of the genome. Nature 459: 927-930.

11. Tweedie S, Ashburner H, Falls K, Leyland P, McQuilton P, et al. (2009) FlyBase: enhancing Drosophila Gene Ontology annotations. Nucleic Acids Research 37: D555-D559.

12. Gelbart WM, Emmert DB (2010) FlyBase High Throughput Expression Pattern Data Beta Version. FlyBase analysis FBrf0212041 (2010.10.13) at `http://flybase.org/reports/FBrf0212041.html`.

13. Schmid M, Davison TS, Henz SR, et al. (2005) A gene expression map of Arabidopsis development. Nature Genetics 37: 501–506.

14. Kilian J, Whitehead D, Horak J, et al. (2007) The AtGenExpress global stress expression data set: protocols, evaluation and model data analysis of UV-B light, drought and cold stress responses. Plant Journal 50: 347–363.

15. Sangurdekar DP, Srienc F, Khodursky AB (2006) A classification based framework for quantitative description of large-scale microarray data. Genome Biology 7: R32.

16. Faith JJ, Hayete B, Thaden JT, Mogno I, et al. (2007) Large-scale mapping and validation of Escherichia coli transcriptional regulation from a compendium of expression profiles. PLoS Biology 5: e8.

17. Jiang P, Singh M (2010) SPICi: a fast clustering algorithm for large biological networks. Bioinformatics 26: 1105–1111.

18. Newman MEJ (2006) Modularity and community structure in networks. Proceedings of the National Academy of Sciences 103: 8577–8582.
